# Investigations of the Evolved Molecular Basis for Terpenoid Biosynthesis in Marine Sponges

**DOI:** 10.1002/advs.77243

**Published:** 2026-08-18

**Authors:** Fangyan Chen, Bao Chen, Wenhui Zhang, Guifang Sun, Yao Zhao, Jianpeng Zhang, Baofu Xu

**Affiliations:** ^1^ Shandong Laboratory of Yantai Drug Discovery Bohai Rim Advanced Research Institute for Drug Discovery Yantai Shandong People's Republic of China; ^2^ Shanghai Institute of Materia Medica Chinese Academy of Sciences Shanghai People's Republic of China; ^3^ Smartgenomics Technology Institute Tianjin People's Republic of China

**Keywords:** biosynthesis, evolution, genome, sponge, terpenoid

## Abstract

Ancestral marine sponges produce terpenoid‐based compounds, and recent studies have begun to reveal the genetic basis in some species, including the first report of type I terpene synthases (T1TSs) in a Bubarida sponge *Obruta collector*. Here, we present a further genomic and functional characterization of terpenoid biosynthesis in the isonitrile terpenoid‐rich sponge *Acanthella cavernosa* by integrating multi‐omics with comparative genomic, phylogenetic, and biochemical analyses. We found that *A. cavernosa* retains the full eukaryotic ancestral mevalonate pathway for terpene precursor biosynthesis. Their single α‐domain classical T1TSs form a basal, independently evolved lineage related to plant T1TS α‐domains, while UbiA‐type TSs drive lineage‐specific structural diversification. Notably, in contrast to the clustering of *T1TSs* with other biosynthetic genes observed in some corals, we did not observe such clustering in the sponge we sequenced, which is consistent with the observation in *O. collector*. Our findings provide additional evidence for the ancestral origin of metazoan terpenoid biosynthesis and further our understanding of early metazoan adaptive evolution, and facilitate biotechnological exploitation of sponge‐derived terpenoids.

## Graphical abstract

Confirming and extending a previous observation in another Bubarida sponge, genomic and functional analyses of *A. cavernosa* reveal that sponges retain the mevalonate pathway and employ single α‐domain T1TSs and UbiA‐type TSs for terpenoid biosynthesis. The absence of *T1TSs* clustering with other biosynthetic genes tentatively suggests, based on limited examples, a distinct genomic organization for terpenoid metabolism in early animals.

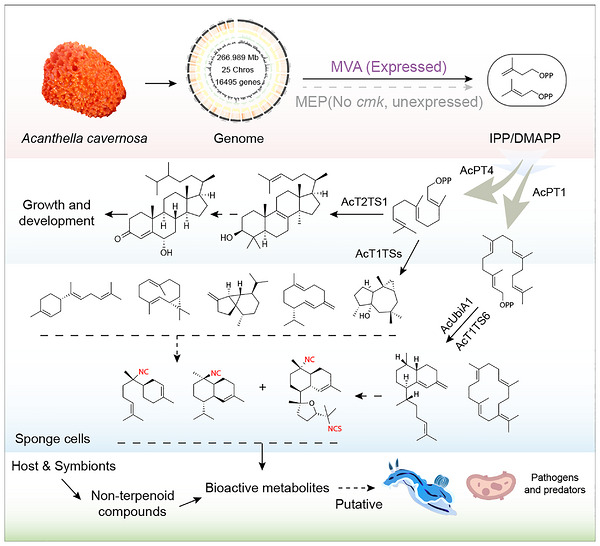

## 1 Introduction

The marine ecosystem constitutes a vast and largely unexplored reservoir of chemical diversity, with marine sponges standing out as premier sources of bioactive natural products. To date, over 9000 unique compounds have been characterized from marine sponges, accounting for approximately 30% of all known marine natural products and encompassing nearly all biosynthetic classes including polyketides, peptides, alkaloids, and terpenoids [1, 2]. As sessile benthic organisms, sponges face significant predation pressure from diverse marine species such as sea slugs, barnacles, starfish, sea turtles, and various fish [3, 4]. Notably, lacking innate immunity, sponges primarily employ bioactive chemicals for potential ecological roles against microbial infections and harsh environmental conditions [5, 6, 7, 8]. These compounds serve crucial ecological functions including feeding deterrence, antioxidation, and antifouling, while simultaneously exhibiting significant pharmacological activities (Figure S1), exemplified by the antimalarial effects of 7,20‐diisocyanoadociane [9] and cytotoxicity properties of Stellettin D [10].

Among all specialized metabolites from sponges, terpenoids are particularly prominent, with proposed roles in mediating spatial competition, deterring predators, and protecting against microbial pathogens and parasites [5, 11]. As the largest class of secondary metabolites, terpenoids account for approximately 4100 of the 9400 known sponge natural products [2, 12]. They are biosynthesized from linear polyprenyl diphosphate precursors, which are first assembled by polyprenyl diphosphate synthases (PTs) and then further cyclized by terpene synthases (TSs) through cationic cascade reactions initiated by diphosphate elimination (type I) or protonation (type II) [13]. Type II TSs (T2TSs), involved in producing essential molecules like sterol triterpenoids for membrane stabilization and hormonal signaling, likely dominate sponge terpenoid biosynthesis, accounting for over 3000 sponge terpenoids, mainly including steroids, carotenoids, and meroterpenoids [12]. Type I TSs (T1TSs) contribute approximately 6% of sponge natural products, whereas roughly 67% of the sponge T1TSs‐derived terpenoids contain characteristic isonitrile, isothiocyanate, or other nitrogen‐containing functional moieties [12, 14, 15].

However, until recently, how marine sessile animals biosynthesize these terpenoids remain largely unclear. In the pre‐genomic era, it was widely hypothesized that terpenoids in sessile animals were synthesized by their symbiotic microorganisms [16]. However, with the development of sequencing technologies, researchers have gradually discovered that corals and sponges themselves possess terpenoid biosynthesis‐related genes, enabling them to synthesize terpenoids de novo [12, 17, 18, 19, 20, 21, 22]. For corals, research groups led by Eric W. Schmidt and Bradley S. Moore independently accomplished the identification and characterization of coral TSs [17, 18]. Our team also successfully identified several terpene synthases derived from a sea‐whip coral and conducted in‐depth mechanistic investigations [19]. Bradley S. Moore et al. reported on the evolutionary trajectory of coral TSs [20]. Beyond terpene synthases, downstream tailoring enzymes have been functionally characterized in recent studies. For instance, a P450 enzyme embedded in the genome of *Heliopora coerulea* can modify the biflorane scaffold to generate aromatic derivatives [21]. Additionally, biosynthetic gene clusters have also been identified in corals [22]. In contrast to terpenoid biosynthesis research in marine corals, analogous studies in sponges have been relatively limited. A notable study by Bradley S. Moore's group previously identified several classical T1TSs that produce typical sponge‐specific sesquiterpene scaffolds [12], which sheds light on the role of sponges in secondary metabolite production and supports the possibility that other sponge‐specific molecules originate from the animal host. On the other hand, the widespread detection of terpenoid metabolites in sponge‐associated fungi across all sponge orders suggests that terpenoid biosynthesis may not be exclusive to the sponge host, highlighting the critical need to resolve the true origins of these compounds [23, 24, 25].

Elucidating the genetic and biosynthetic basis of sponge terpenoid production urgently requires high‐quality genomic and transcriptomic data. However, generating such data from marine sponges has proven exceptionally challenging due to their complex biology: as multicellular organisms with intricate genomic architectures, sponges form symbioses with diverse microorganisms and algae, have genome sizes ranging from hundreds of megabases to several gigabases, and contain abundant repetitive sequences. Additionally, contamination from microbial DNA and DNA‐bound compounds severely impede sequencing efforts [26]. To overcome these obstacles, several strategies have been employed, including isolating DNA from larvae with lower microbial loads [27] and whole‐genome amplification from single cells [28], although these often yield highly fragmented assemblies. Thanks to recent advances in sequencing technologies, including long‐read, synthetic long‐read, and Hi‐C approaches, chromosome‐level assemblies are now achievable, as demonstrated for *Ephydatia muelleri* [29], *Petrosia ficiformis*, and *Chondrosia reniformis* [30]. Despite these advances, only 12 sponge genomes have been sequenced out of over 9500 described species, 13 years after the first genome was published [31]. In addition, chromosome‐level studies of terpenoid biosynthesis in sponges remain unexplored; most terpenoid synthase identification rely on metagenomic and meta‐transcriptomic data [12], limiting our understanding of host‐encoded biosynthetic pathways.

Inspired by the identification of terpene synthase genes from the nitrogenous terpenoid‐containing Bubarida sponge by Bradley S. Moore's group [12], we extend these findings by leveraging high‐quality genomic and transcriptomic data from the isonitrile terpenoid‐rich marine sponge *Acanthella cavernosa*, and conducting comparative analyses across four sponge classes to elucidate the molecular mechanisms underpinning terpene biosynthesis in these ancient metazoans. Our results establish that sponges, as the earliest‐branching metazoans, evolutionarily retained the complete mevalonate (MVA) pathway for C_5_ terpene precursor production. Synthetic biology‐driven functional characterization identified several PTs, classical T1TSs, non‐canonical UbiA‐type TSs, and T2TSs, which are responsible for synthesizing the terpene precursors and scaffolds essential for ecological interactions and growth. Phylogenetic and structural analyses further revealed that sponge T1TSs contain only the α domain, evolve independently, and show closer affinity to the α domains of plant TSs. Notably, unlike the situation in some corals, where *T1TSs* are genomically clustered with other biosynthetic genes, such clustering was not observed for the *T1TSs* in *A. cavernosa—*a pattern consistent with the previous observation in *O. collector*. Our results demonstrate that integrative genomic‐transcriptomic analyses can effectively disentangling the genetic basis of terpenoid biosynthesis in marine sessile organisms.

## 2 Results

### 2.1 Chromosome‐Level Genome Assemblies for *A. cavernosa*

**FIGURE 1:**
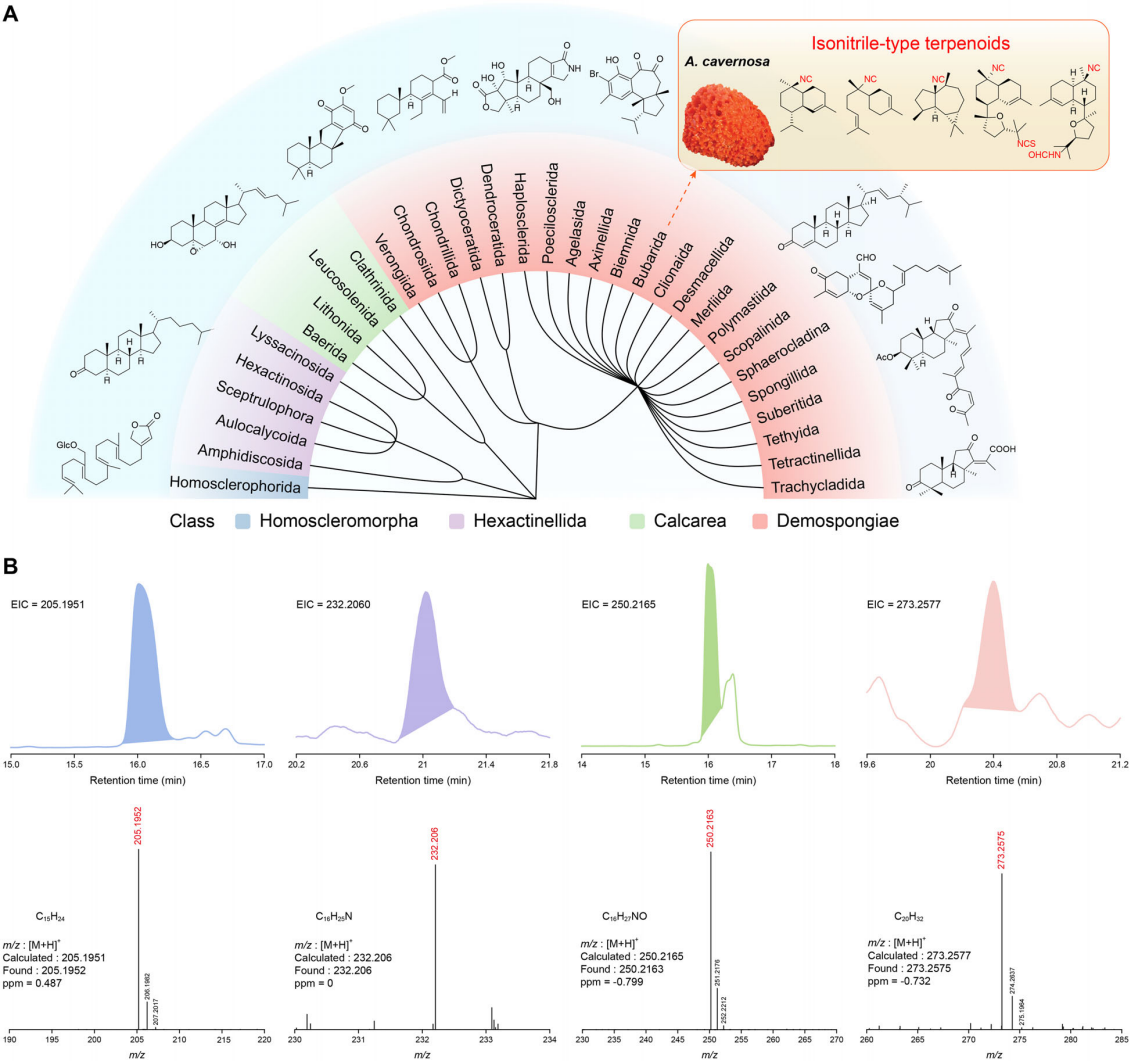
Isonitrile‐type compounds profiling of *A. cavernosa* sponge. (A) Sponges of the species *A. cavernosa* within the order *Bubarida* produce a special class of toxic isonitrile‐containing compounds. (B) UPLC‐HRMS analysis of total compounds from *A. cavernosa*. Extracted ion chromatograms (EIC, up) and corresponding mass spectra (MS, down) for the molecular formulas C_15_H_24_ (*m/z* 205.1951), C_16_H_25_N (*m/z* 232.2060), C_16_H_27_NO (*m/z* 250.2165), and C_20_H_32_ (*m/z* 273.2577) were shown (n  =  3 biological replicates and one representative data were shown). The chemical structures of previously reported compounds isolated from *A. cavernosa* corresponding to these molecular masses are presented in Figure S3.

To elucidate the evolutionary molecular basis of terpenoid biosynthesis in marine sponges, we selected *A. cavernosa* (Figure S2), a member of the order *Bubarida* (class Demospongiae) and a well‐documented producer of nitrogen‐containing terpenoids, including nitriles (‐CN), isonitriles (‐NC), isothiocyanates (‐NCS), isocyanates (‐NCO), and formamides (‐NHCHO) [32, 33, 34, 35]. Many of these compounds exhibit potent cytotoxicity and substantial therapeutic potential (Figure 1A) [15, 36, 37]. After sample collection, we analyzed total metabolites extracted from *A. cavernosa* via ultra‐performance liquid chromatography‐tandem high‐resolution mass spectrometry (UPLC‐HRMS) (Figure 1B). The extracted ion chromatograms and corresponding mass spectra, matched to the molecular formulas of previously reported compounds isolated from *A. cavernosa* (Figure S3) [34, 38, 39, 40], confirmed the sponge's capacity to biosynthesize these characteristic nitrogenous terpenoids. It should be noted that the mass spectrometric data indicate the presence of compounds with molecular formulas matching previously reported terpenoids, although full structural confirmation by NMR awaits further analysis due to limited biomass.

**FIGURE 2:**
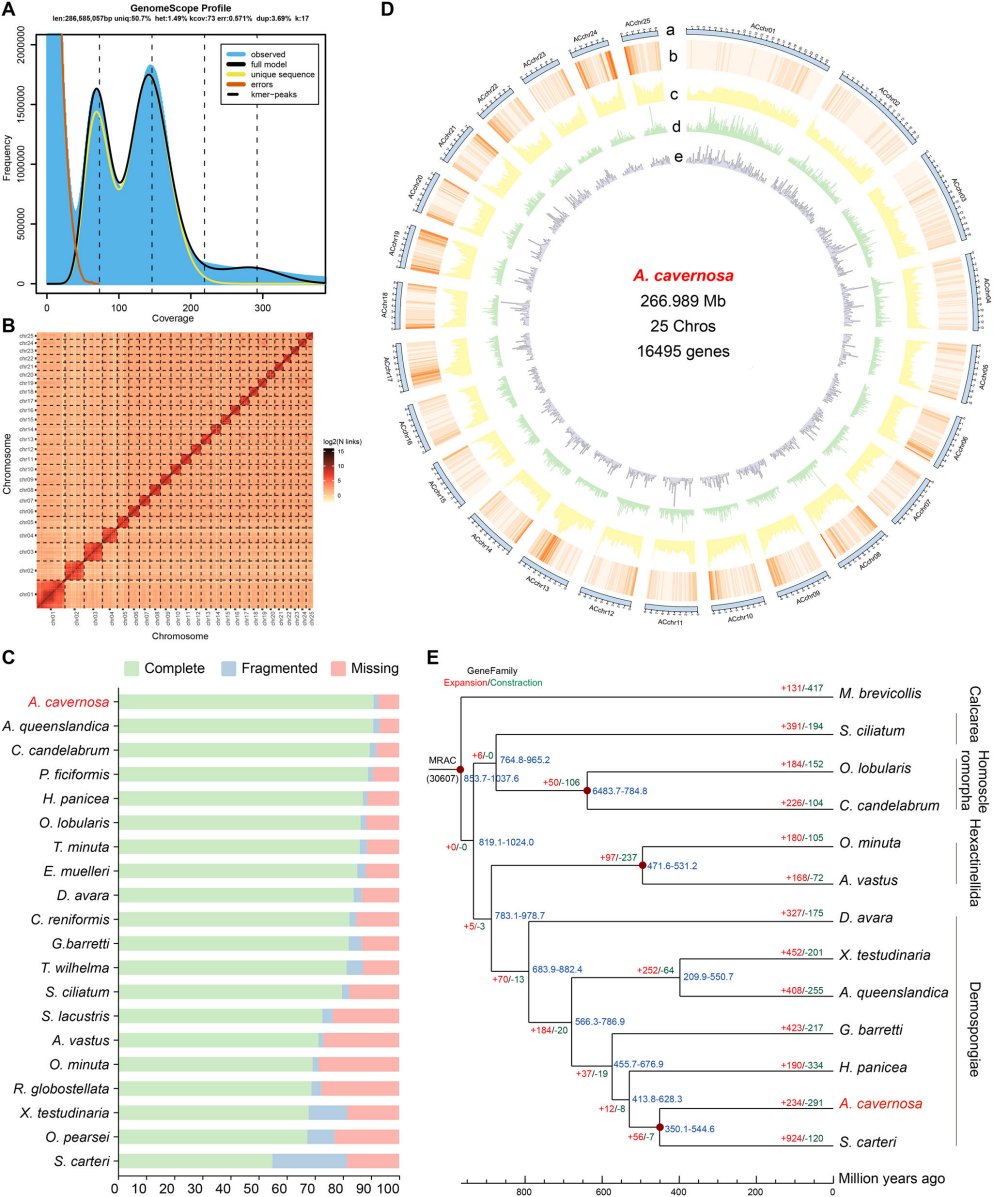
Genome assembly and genomic evolution of the *A. cavernosa* sponge. (A) 17 K‐mer frequency distribution of *A. cavernosa*. (B) Hi‐C interaction heatmap of the *A. cavernosa* genome. Colors represent log_2_‐transformed normalized interaction frequency (log_2_(N links)), ranging from low (yellow) to high (dark red). (C) The comparison of genome BUSCO of *A. cavernosa* with other published sponge genomes. Complete, fragmented and missing Buscos were shown. (D) Genomic landscape of the 25 assembled pseudochromosomes. Track a represents the length of the pseudochromosomes (Mb), b‐e represent gene density, GC content, Gypsy retrotransposon and Copia retrotransposon density respectively, these metrics are calculated in 200 kb windows. (E) Genomic evolution of *A. cavernosa* with other 11 selected sponges. *M. brevicollis* was set as an outgroup. The red (blue) numbers on the branches of the phylogenetic tree indicate the number of expanded (contracted) gene families. The purple numbers indicate divergence time. Branch lengths are proportional to divergence time as estimated by MCMCtree (scale bar at bottom).

To unravel the genetic and enzymatic underpinnings of this specialized metabolic pathway, we generated a high‐quality chromosome‐scale genome assembly of *A. cavernosa* using PacBio HiFi sequencing combined with Hi‐C scaffolding. K‐mer analysis with Illumina short reads estimated the genome size of *A. cavernosa* to be approximately 286 Mb with a heterozygosity of 1.49% (Figure 2A). A total of 43.14 Gb of PacBio Revio HiFi reads (∼162× coverage) and 57.8 Gb of Hi‐C data were generated (Table 1 and Table S1). A total of 43.14 Gb of RNA‐seq data were generated (Tables S1 and S2). De novo assembly of third‐generation long‐read data initially produced an assembly of 267,387,833 bp comprising 355 contigs. To remove potential non‐sponge sequences, contigs were further evaluated using taxonomic classification and transcriptomic evidence. After retaining only contigs assigned to eukaryotes and supported by transcriptome read alignments, the curated assembly was refined to 266,866,407 bp across 347 contigs. Incorporation of Hi‐C data enabled chromosome‐scale scaffolding of the filtered assembly, anchoring 271 contigs onto 25 pseudochromosomes, which collectively accounted for 94.13% (251,200,346 bp) of the total non‐gap sequences (Figure 2B; Tables S3 and S4). During Hi‐C‐based scaffolding, approximately 123 kb of gap sequences (Ns) were introduced between adjacent contigs, resulting in a final scaffolded assembly size of 266,989,407 bp (Table 1). In comparison with 19 other sponge genomes (Tables S5–S7), the *A. cavernosa* assembly exhibited improved contig continuity, as reflected by a higher contig N50, and achieved the highest BUSCO completeness (91%) score among the datasets examined (Figure 2C).

**TABLE 1:** Summary of *A. cavernosa* chromosome level assembly and annotation.

Sequencing	
Raw bases from WGS‐DNBSEQ‐T7(Gb)	20(74.9x)
Raw bases from WGS‐PacBio Revio (Gb)	43.14(162.2x)
Raw bases from Hi‐C (Gb)	57.8(216.6x)
HiFi Read Length (mean, bp)	14,837
HiFi Read Length (median, bp)	14,088
HiFi Read Length N50 (bp)	14,609
HiFi Read Quality (median)	Q37

Assembly	
Assembled genome size (bp)	266,989,407
Number of contigs	347
Number of Scaffolds	101
Number of chromosomes	25
Contig N50 (bp)	2,695,861
Scaffold N50 (bp)	9,154,159
Average Scaffold length (bp)	2,102,278
GC content (%)	42.84
Anchorage to chromosomes (%)	94.13
Complete genome BUSCOs (%)	91

Annotation	
Percentage of repeat sequences (%)	54.75
LTR rate (%)	38.23
Number of predicted genes	16,495
Average gene length (bp)	8,745.47
Average CDS length (bp)	1,400.04
Average exon length (bp)	202.92
Average intron length (bp)	1,245.08

Repeat sequences account for 54.75% of the *A. cavernosa* genome, of which long terminal repeats (LTRs) represented 38.23%, long interspersed nuclear elements (LINEs) 4.75%, and DNA transposons 2.57% (Figure S4 and Table S8). Using a combination of *de novo* prediction, homology‐based annotation, and transcriptome evidence, a total of 16,495 protein‐coding genes were generated, with an average gene length of 8,745 bp and an average coding sequence (CDS) length of 1,400 bp (Table 1 and Table S9). Gene and repeat densities were inversely correlated in the *A. cavernosa* genome, with higher gene densities at the distal regions of the chromosomes (Figure 2D). Functional annotation revealed that 15,322 predicted genes (92.89%) were annotated in at least one major database, including NR, SwissProt, KEGG, TrEMBL, InterPro and GO (Table S10). BUSCO analysis indicated high gene completeness in *A. cavernosa*, with 839 of 954 core genes (87.9%) complete, reflecting relatively good annotation among sponge genomes (Tables S7 and S11).

To conduct genomic evolutionary analysis, we selected 11 additional Porifera species, including 1 Calcarea (*Sycon ciliatum*), 2 Homoscleromorpha (*Corticium candelabrum*, *Oscarella lobularis*), 2 Hexactinellida (*Oopsacas minuta* and *Aphrocallistes vastus*), and 6 Demospongiae (*Amphimedon queenslandica*, *Halichondria panicea*, *Dysidea avara*, *Geodia barretti*, *Xestospongia testudinaria*, and *Stylissa carteri*), along with 1 choanoflagellate (*Monosiga brevicollis*) (Table S12). Phylogenomic analyses revealed a close evolutionary relationship between *A. cavernosa* and *S. carteri* (Figure 2E). The earliest divergence of sponges was estimated to have occurred 1,037.6 million years ago. In terms of gene family dynamics, *A. cavernosa* displayed more contracted gene families than expanded ones (Figure 2E).

### 2.2 Sponges Evolutionarily Retained the Complete MVA Pathway for C_5_ Terpene Precursor Production

Terpenoids, which are derived from the common five‐carbon isoprene precursors dimethylallyl diphosphate (DMAPP) and its isomer isopentenyl diphosphate (IPP), are naturally synthesized through two distinct metabolic pathways: the MVA pathway and the MEP pathway. The MVA pathway initiates with acetyl‐CoA and proceeds through seven enzymatic steps, sequentially catalyzed by acetyl‐CoA C‐acetyltransferase (AACT), 3‐hydroxy‐3‐methylglutaryl‐CoA synthase (HMGS), HMG‐CoA reductase (HMGR), mevalonate kinase (MVK), phosphomevalonate kinase (PMK), mevalonate‐5‐diphosphate decarboxylase (MVD), and IPP isomerase (IDI), ultimately yielding both IPP and DMAPP. In contrast, the MEP pathway converts pyruvate and D‐glyceraldehyde 3‐phosphate (GAP) into IPP and DMAPP via a series of seven enzymes: 1‐deoxy‐D‐xylulose 5‐phosphate synthase (DXS), DXP reductoisomerase (DXR), 2‐C‐methyl‐D‐erythritol 4‐phosphate cytidylyltransferase (CMS), 4‐(cytidine 5′‐diphospho)‐2‐C‐methyl‐D‐erythritol kinase (CMK), 2‐C‐methyl‐D‐erythritol 2,4‐cyclodiphosphate synthase (MDS), 4‐hydroxy‐3‐methylbut‐2‐enyldiphosphate synthase (HDS), and HMBPP reductase (HDR). In organisms that possess both pathways, such as plants, subcellular localization differs: most MVA enzymes are cytosolic, whereas all MEP enzymes are plastid‐associated.

**FIGURE 3:**
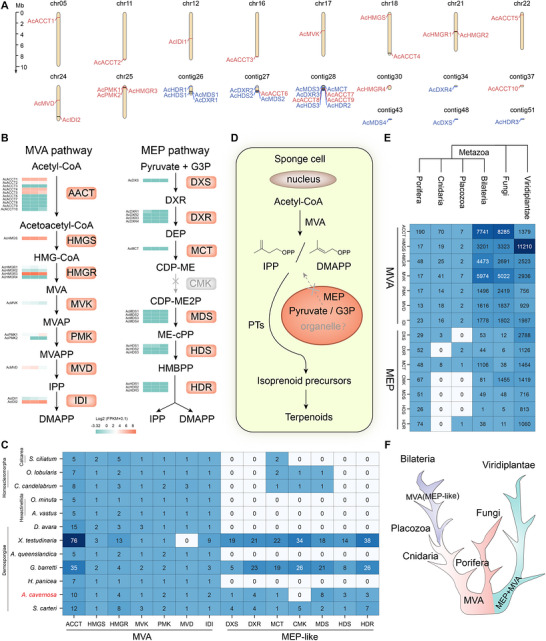
The formation patterns of five‐carbon isoprene precursors in sponges. (A) Chromosomal localization of precursor synthesis genes in *A. cavernosa*. Genes involved in the MVA pathway are shown in red, and MEP‑like sequences are shown in blue. (B) The presence and expression levels of MVA pathway genes and MEP‑like sequences in the *A*. cavernosa sponge using three biological replicates (n = 3). The *cmk*, which was not found among the MEP‑like sequences, is denoted by a gray block. Colors represent log_2_(FPKM+0.1) expression levels, ranging from cyan (low) to red (high). (C) Counts of MVA pathway genes and MEP‑like sequences in the 12 sponges analyzed for genomic evolution. Color shading from light to dark indicates an increasing number of genes. (D) A model of terpenoid precursor IPP and DMAPP formation in sponge cells. (E) Counts of MVA pathway genes and MEP‑like sequences in porifera, cnidaria, placozoa, bilateria, fungi and viridiplantae. (F) Schematic representation of the differences in the utilization of the MVA or MEP pathways for forming terpenoid precursors among metazoa, plants, and fungi.

Genomic mining of *A. cavernosa* revealed complete MVA pathway gene sets but no clear evidence for host‑encoded MEP pathway components; while sequences with similarity to MEP genes (hereafter referred to as MEP‑like sequences) were detected, the critical *cmk* was absent from the assembled genome (Figure 3A). Examination of genomic localization showed that MVA pathway genes were chromosomally anchored and expressed, while the MEP‑like sequences were distributed solely on contigs and exhibited minimal transcriptomic activity (Figure 3A,B). Notably, subcellular localization predictions indicated mitochondrial targeting signals only in AACT genes; all other MVA pathway components and the MEP‐like sequences lacked targeting sequences (Table S13).

A broader survey across 11 additional sponge species (spanning Homoscleromorpha, Calcarea, Hexactinellida, and Demospongiae) confirmed consistent presence of MVA pathway genes, except for the absence of *mvd* in *X. testudinaria*, whereas most sponges lacked a complete set of MEP‑like sequences (Figure 3C). These findings collectively support the conclusion that sponges predominantly rely on the MVA pathway for terpenoid isoprenoid precursor synthesis (Figure 3D). Comparative profiling of MVA and MEP pathway genes across Metazoa (including sponges, corals, placozoans, and bilaterians), Fungi, and Viridiplantae in UniProt further revealed a predominance of MVA pathway genes in Cnidaria, Placozoa, Bilateria, and Fungi, with Viridiplantae retaining both pathways (Figure 3E). This underscores the evolutionary conservation of MVA‐mediated C_5_ precursor biosynthesis in fungi and metazoans, while highlighting the lineage‐specific retention of dual pathways in plants (Figure 3F).

### 2.3 AcPT4 and AcPT1 Convert C_5_ Precursors into FPP and GGPP, Respectively

**FIGURE 4:**
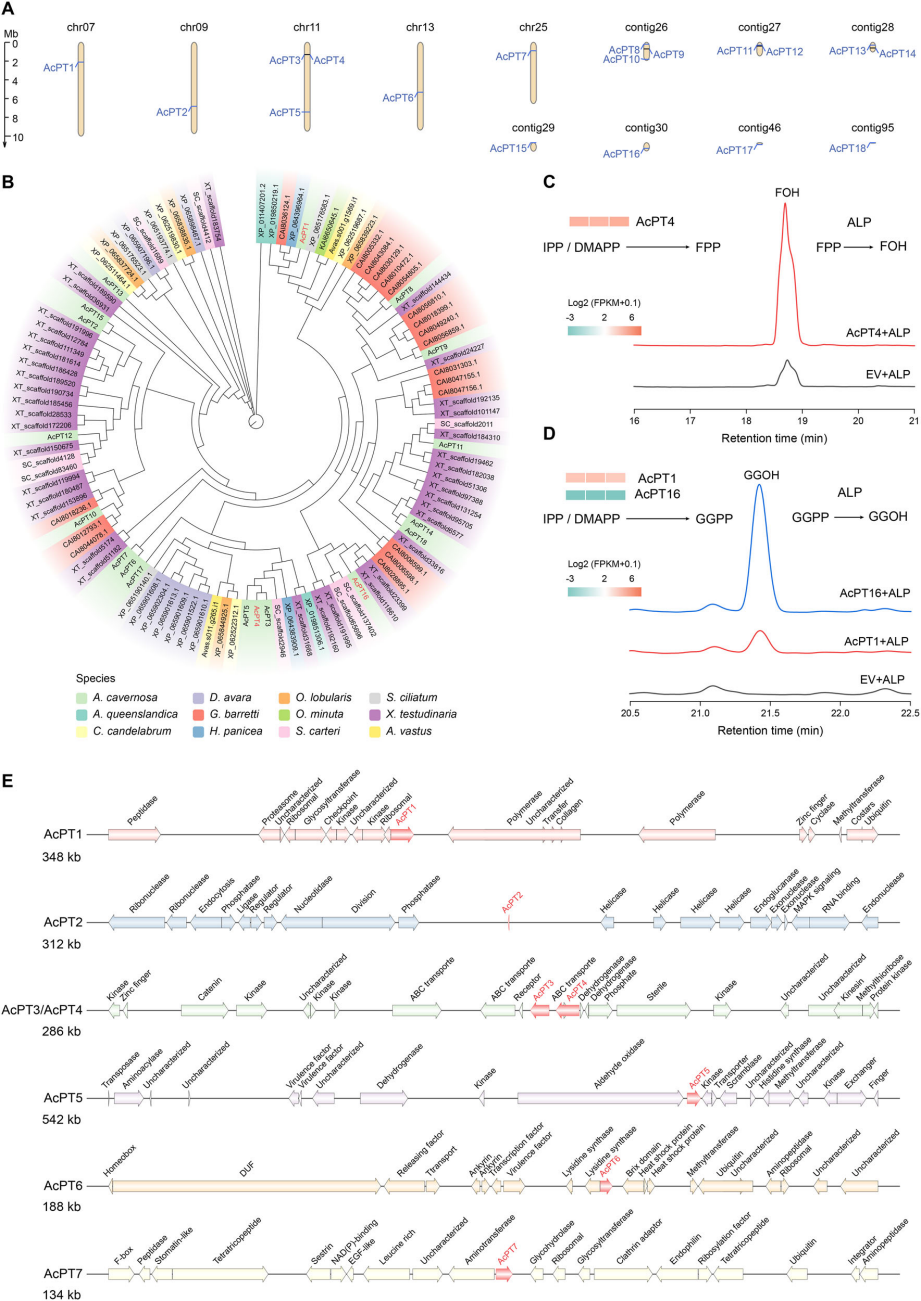
Analysis and functional characterization of the PT gene family. (A) Chromosomal localization of *PTs*. (B) Maximum‐Likelihood phylogeny of PTs (Dataset1) identified from 12 sponges analyzed for genomic evolution. Bootstrap values (1,000 replicates) were calculated but are not shown on the tree. Functionally characterized PTs in *A. cavernosa* are indicated in red. (C) AcPT4 catalyzes the formation of FPP from the terpenoid precursors IPP and DMAPP. AcPT4 was co‐expressed with alkaline phosphatase in an *E. coli* terpene precursor chassis, and the fermentation products were analyzed by HPLC. The co‐expression of an empty vector with alkaline phosphatase served as the negative control. Alkaline phosphatase (ALP) catalyzes the conversion of FPP to farnesol (FOH). The expression levels of *AcPT4* were examined in three biological replicates (n = 3) of the *A. cavernosa* sponge. Colors represent log_2_(FPKM+0.1) expression levels, ranging from cyan (low) to red (high). (D) AcPT1 and AcPT16 catalyze the formation of GGPP from IPP and DMAPP. The co‐expression with alkaline phosphatase, fermentation, and detection methods were the same as described in (C). The expression levels of *AcPT1* and *AcPT16* were examined in three biological replicates (n = 3) of the *A. cavernosa* sponge. Colors represent log_2_(FPKM+0.1) expression levels, ranging from cyan (low) to red (high). (E) *PTs* in *A. cavernosa* are located on the chromosomes, with no terpenoid‐modifying genes present in their vicinity. The *PTs* are indicated by red arrows. The genomic context shown represents the 10 genes upstream and downstream of each *PT* locus.

Prenyltransferases (PTs) are essential enzymes that synthesize isoprenoid precursors of varying chain lengths, including geranyl diphosphate (GPP; C_10_), farnesyl diphosphate (FPP; C_15_), and geranylgeranyl diphosphate (GGPP; C_20_), by catalyzing the sequential condensation of IPP and DMAPP [41]. In the genome of *A. cavernosa*, we identified 18 PTs, with seven located on assembled chromosomes (AcPTs1–7) and the remaining 11 on unassembled contigs (Figure 4A). Systematic mining of PT genes across 11 additional sponge genomes confirmed their ubiquitous presence in this phylum. Phylogenetic analysis revealed that these PT genes cluster into multiple clades, with *A. cavernosa* PTs distributed across several clades, indicating a high degree of functional conservation among sponges (Figure 4B).

To validate the function of PTs in *A. cavernosa*, we co‐expressed them with alkaline phosphatase in an *E. coli* chassis engineered for elevated production of C_5_ terpene precursors. HPLC analysis demonstrated that AcPT4 specifically catalyzes FPP formation, while both AcPT1 and AcPT16 produce GGPP. Notably, AcPT16, despite its activity in the heterologous expression system, was not detected in *A. cavernosa* transcriptomes, suggesting that AcPT1 is the primary mediator of GGPP synthesis in vivo (Figure 4C,D).

Terpenoid biosynthetic genes are frequently organized into genomic clusters to facilitate efficient substrate channeling and coordinate regulation. However, detailed examination of the genomic loci flanking PT genes in *A. cavernosa* revealed no associated terpene backbone or modification enzymes. This observation suggests that, unlike many other organisms, terpenoid biosynthetic genes in sponges may not typically be organized into biosynthesis gene clusters (Figure 4E).

### 2.4 Ancient AcT1TSs and AcUbiA1 Generate Terpene Scaffolds

**FIGURE 5:**
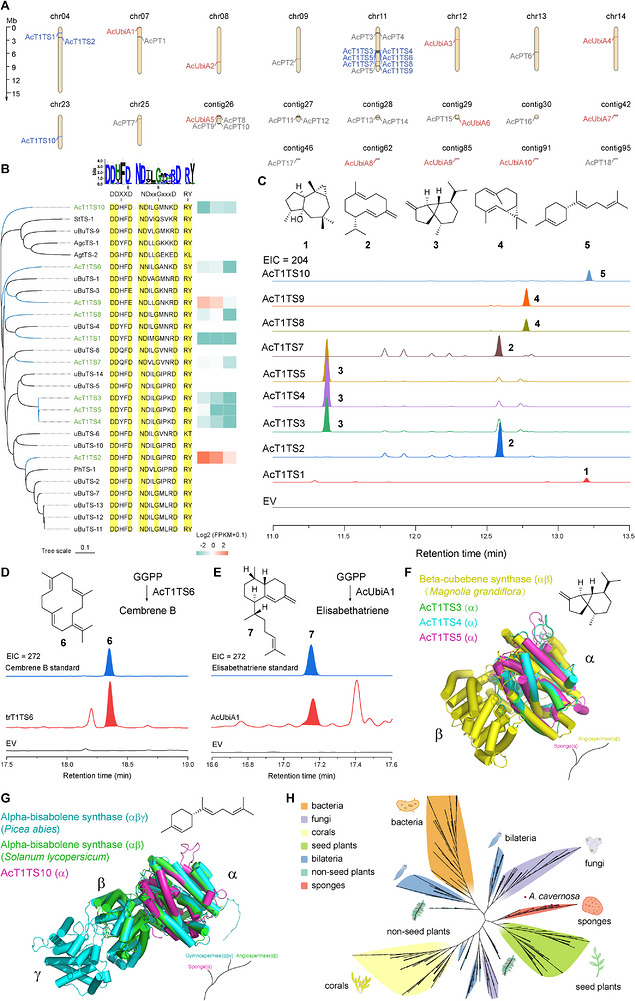
Functional and phylogenetic analysis of T1TSs. (A) Chromosomal localization of *PTs*, *UbiAs*, and *T1TSs* genes in *A. cavernosa*. *PTs* are shown in gray, *UbiAs* in red, and *T1TSs* in blue. (B) Neighbor‐Joining phylogeny of T1TSs in *A. cavernosa* and previously reported sponge T1TSs by Bradley S. Moore's group [12]. T1TSs in *A. cavernosa* are marked in green, and their expression levels were examined in three biological replicates (n = 3). Colors represent log_2_(FPKM+0.1) expression levels, ranging from cyan (low) to red (high). The conserved motifs (DDxxD, NDxxGxxxD, and RY) of these T1TSs, along with the expression levels of T1TSs in *A. cavernosa*, are shown on the right. (C) Extracted ion chromatograms (*m/z* = 204) of GC‐MS analysis of metabolites produced by *E. coli* chassis engineered to overexpress FPP along with T1TSs or empty vector (EV) control. The corresponding chemical structures of product **1** (generated by AcT1TS1), product **2** (by AcT1TS2 and AcT1TS7), product **3** (by AcT1TS3, AcT1TS4, and AcT1TS5), product **4** (by AcT1TS8 and AcT1TS9), and product **5** (by AcT1TS10) are displayed above the chromatograms. Notably, AcT1TS2 and AcT1TS7 produce the same product as PhTS‐1 and uBuTS‐2, whereas AcT1TS8 and AcT1TS9 yield the enantiomer of the product from uBuTS‐1 and uBuTS‐3 [12]. (D) Extracted ion chromatograms (*m/z* = 272) of GC‐MS‐analyzed extracted metabolites from truncated AcT1TS6 or empty vector (EV) overexpressed together with tHMGR in yeast YPH499. Standard compound cembrene B was used as a positive control, and its chemical structure is displayed on the top. (E) Extracted ion chromatograms (*m/z* = 272) of GC‐MS analysis of metabolites produced by *E. coli* chassis engineered to overexpress GGPP along with AcUbiA1 or empty vector (EV) control. Standard compound elisabethatriene was used as a positive control, and its chemical structure is displayed on the top. (F) Structural alignment between the SWISS‐MODEL predicted structures of AcT1TS3, AcT1TS4, AcT1TS5 (produce (+)‐*β*‐cubebene) and the *β*‑cubebene synthase from *M. grandiflora*. An evolutionary schematic of the *β*‑cubebene synthase structure from sponges to angiosperms is shown in the lower right. (G) Structural alignment between the SWISS‐MODEL predicted structures of AcT1TS10 (produce (‐)‐(*E*)‐*α*‐bisabolene) and the *α*‐bisabolene synthase from *Picea abies* and *Solanum lycopersicum*. An evolutionary schematic of the *α*‐bisabolene synthase structure from sponges to gymnospermae and angiospermae is shown in the lower right. (H) Maximum‑likelihood phylogeny of T1TSs (Dataset2) from sponges (n = 30), corals (n = 100), bilateria (n = 100), bacteria (n = 100), fungi (n = 100), non‐seed plants (n = 35), and seed plants (n = 100). Bootstrap values (1,000 replicates) were calculated but are not shown on the tree.

Terpene synthases catalyze the cyclization of linear isoprenoid precursors into diverse terpene scaffolds via cationic cascade reactions. These reactions are initiated by either diphosphate dissociation (T1TSs) or double bond protonation (T2TSs) [42]. To identify T1TSs in *A. cavernosa*, we performed HMMER searches against its proteome using the canonical T1TS Pfam domain (PF19086) and subsequent homology alignment with a curated database of marine animal T1TSs (Table S14). This analysis led to the identification of 10 *T1TSs*, seven of which (*AcT1TSs3*–*9*) form a cluster on chromosome 11 (Figure 5A). Among these 10 *T1TSs*, only *AcT1TS6* contains an intron; notably, genes flanking all *T1TSs* loci are predominantly intron‐containing and eukaryote‐specific (e.g., translation initiation factors, sterile alpha motif (SAM) domain‐containing proteins, and formins) (Figure S5). This confirms that the identified *T1TSs* are endogenous to the sponge.

Multiple sequence alignment of key active site residues across sponge T1TSs revealed conservation of canonical Class I TS motifs, including the aspartate‐rich DDxxD motif, (N/D)Dxx(S/T)xxx(D/E) (NSD/DTE) motif, and C‐terminal RY dimer. Notably, we found that the NSE/DTE motif in sponge T1TSs predominantly assumes an NDxxGxxxD sequence, while the RY dimer incorporates distinctive residue pairs such as KL, SY, and KT (Figures 5B and S6). These alterations suggest a divergent evolutionary trajectory that has shaped lineage‐specific conserved signatures in marine sponge T1TSs. Nine of the 10 T1TSs share identical conserved active site motifs: NDxxGxxxD and RY. In contrast, AcT1TS6 harbors variant motifs (NNxxGxxxD and SY), suggesting functional divergence from the other AcT1TSs (Figure 5B). Transcriptomic analysis confirmed the expression of all T1TSs in *A. cavernosa* (Figure 5B).

To validate T1TS functions, we first heterologously expressed each T1TS in an *E. coli* chassis engineered for high‐level production of distinct isoprenoid precursors (GPP, FPP, GGPP, geranylfarnesyl diphosphate (GFPP), or hexaprenyl diphosphate (HexPP)). We analyzed the metabolite profiles of these engineered strains via HPLC; novel compounds were further subjected to large‐scale fermentation, in vitro isolation/purification, and structural elucidation by NMR spectroscopy. The results showed: AcT1TS1 produces (+)‐isoafricanol (**1**); AcT1TS2 and AcT1TS7 produce (‐)‐germacrene D (**2**), the same product as previously reported terpene synthases PhTS‐1 and uBuTS‐2 [12]; AcT1TS3, AcT1TS4, and AcT1TS5 produce (+)‐*β*‐cubebene (**3**); AcT1TS8 and AcT1TS9 yield (‐)‐bicyclogermacrene (**4**), the enantiomer of the product from the previously reported terpene synthases uBuTS‐1 and uBuTS‐3 [12]; and AcT1TS10 produces (‐)‐(*E*)‐*α*‐bisabolene (**5**) (Figure 5C). All product structures were confirmed by NMR (Figures S7–S21 and Table S15). AcT1TS6 exhibited no catalytic activity in *E. coli*; thus, we expressed a truncated AcT1TS6 variant in *Saccharomyces cerevisiae* YPH499. Fermentation and GC‐MS analysis revealed that AcT1TS6 produces cembrene B (**6**), which was verified by comparison with an authentic cembrene B standard (Figures 5D and S22).

Notably, biflorane, the characteristic diterpenoid scaffold of *A. cavernosa*, was not produced by any T1TS. Given that UbiA family proteins have also been reported to initiate terpene cyclization [43, 44], we examined the UbiA homologs in *A. cavernosa* and confirmed through sequence and structural alignments that they retain the canonical UbiA‐type terpene synthase motifs: NQxxG/AxxED (motif I) and DxxDxxxD (motif II) (Figures S23–S25). We therefore functionally characterized all 10 UbiA genes identified in this sponge (Figure 5A). Heterologous expression of these UbiAs in an *E. coli* terpene precursor chassis, followed by GC‐MS analysis, revealed a product that matched the retention time and mass spectrum of an authentic elisabethatriene standard, which possesses the biflorane scaffold (Figure 5E; Figures S26 and S27).

Sequence alignment of AcT1TSs3–5 with *β*‐cubebene synthase from the angiosperm *Magnolia grandiflora* revealed a moderate degree of similarity (21.5‐21.9% i.d.; Figure S28 and Table S16), indicating a cryptic evolutionary history shared between these distantly related terpene synthases. To dissect structural divergences and conserved features between sponge‐derived and angiosperm *β*‐cubebene synthases, we homology‐modeled three sponge enzymes (AcT1TS3, AcT1TS4, AcT1TS5) and the *M. grandiflora β*‐cubebene synthase using SWISS‐MODEL, followed by structural superposition. This analysis demonstrated a modest degree of overall fold conservation across all four enzymes; however, sponge T1TSs contain only an α‐domain, whereas the angiosperm enzyme possesses both α‐ and β‐domains (Figure 5F and Table S17). *α*‐Bisabolene synthases have also been described in angiosperms and gymnosperms. Structural prediction and alignment of *α*‐bisabolene synthases from sponges and plants similarly showed modest conservation, with sponge AcT1TS10 containing only an α‐domain, angiosperm enzymes harboring α‐ and β‐domains, and gymnosperm synthase possessing three domains (α, β, and γ) (Figures 5G and S29; Tables S17 and S18). These findings indicate that the α‑domain is present in sponge T1TSs as the sole domain, which is consistent with an ancient origin.

In addition, most sponge *T1TSs* are intron‐less, raising the question of whether they are evolutionarily closer to microbial homologs. To resolve the phylogenetic position of sponge T1TSs, we performed phylogenetic analysis of T1TSs across diverse taxonomic kingdoms, including corals, bacteria, fungi, non‐seed plants (lycophytes and ferns), and seed plants. The phylogeny showed that sponge T1TSs form a distinct monophyletic clade sister to seed plant T1TS clades (Figure 5H). This indicates that sponge T1TSs evolved independently and share greater similarity with plant T1TSs than with microbial counterparts.

### 2.5 AcT2TS1 Produces Lanosterol, While AcT2TS2 is a Putative Bacterial‑Like Locus

**FIGURE 6:**
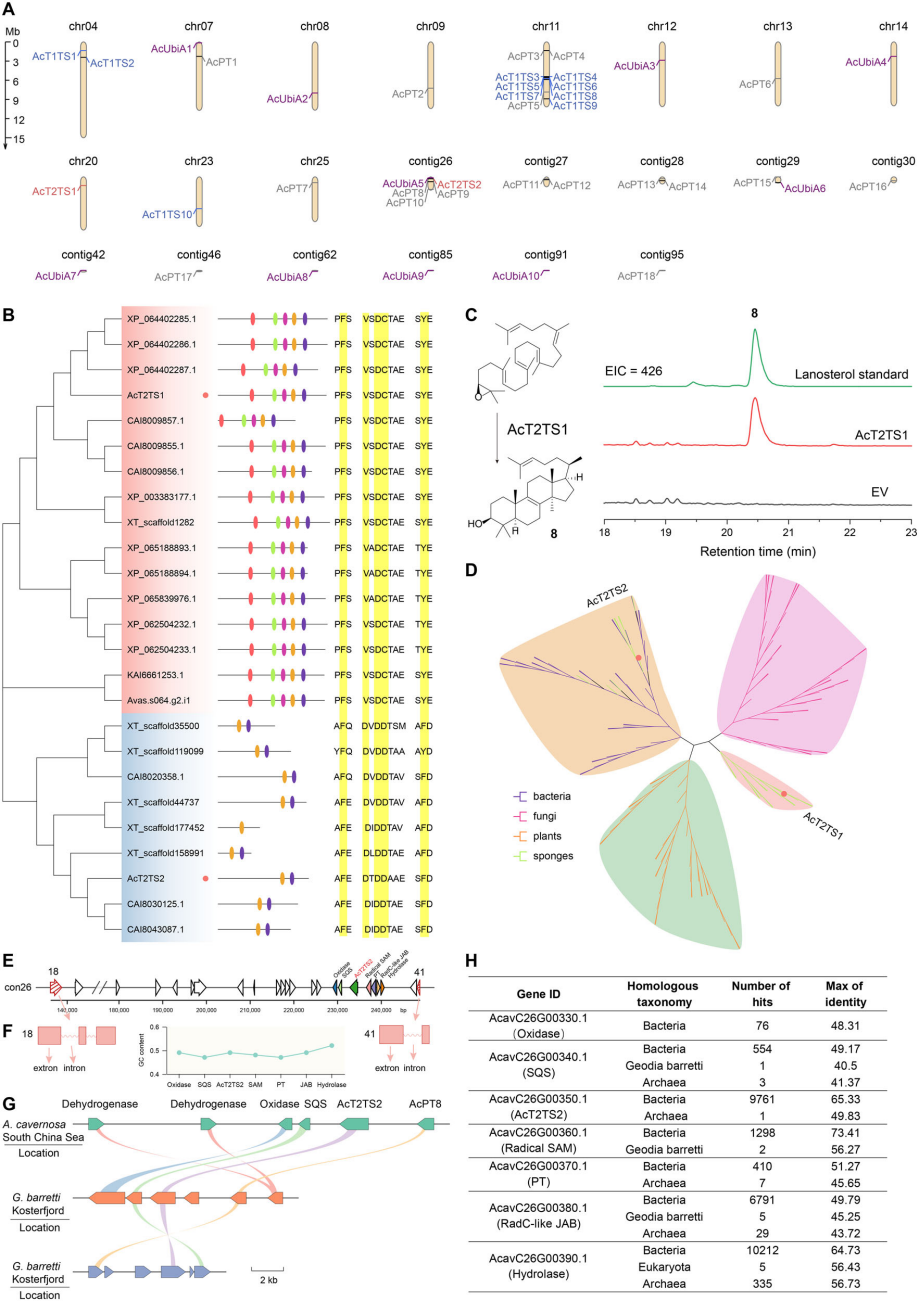
Functional and phylogenetic analysis of AcT2TSs. (A) Chromosomal localization of *PTs*, *UbiAs*, *T1TSs* and *T2TSs*. *T2TSs* in *A. cavernosa* are shown in pink. (B) Neighbor‐Joining phylogeny of T2TSs identified through IPR018333 from 12 sponges. Bootstrap values (1,000 replicates) were calculated but are not shown on the tree. (C) Extracted ion chromatograms (*m/z* = 426) of GC‐MS‐analyzed extracted metabolites from AcT2TS1 or empty vector (EV) overexpressed in yeast GIL77. Compound lanosterol standard was used as a positive control, and the reaction directed by AcT2TS1 from 2,3‐oxidosqualene to lanosterol is displayed on the left. (D) Maximum‑likelihood phylogeny of T2TSs (Dataset4) from bacteria (n = 41), fungi (n = 49), plants (n = 40; all retrieved from UniProt via IPR018333), and sponges (n = 25; identified via IPR018333 from 12 sponge genomes). Bootstrap values (1,000 replicates) were calculated but are not shown on the tree. The leaves of sponges are highlighted in green, with AcT2TSs marked by red dots. (E) Annotation and intron‐containing status of genes neighboring *AcT2TS2*. Intron‐containing genes (*18* and *41*) are marked in red and with a slash, while the remaining genes lack introns. (F) Genomic organizations of genes *18* and *41*, and GC content of genes flanking *AcT2TS2*. (G) Microsynteny between the *AcT2TS* gene cluster and the terpenoid gene cluster in the *G. barretti* sponge (collected in Kosterfjord), both predicted by antiSMASH. Scale bar, 2 kb. (H) Homologous taxonomy and highest similarity of homologous proteins (identity >40%) identified by NCBI blast for proteins flanking AcT2TS2.

Lanosterol‐type compounds have been identified in the sponge *A. cavernosa* [45]. To elucidate their biosynthetic provenance, we characterized the functional roles of T2TS enzymes in *A. cavernosa*. Via IPR018333 domain annotation‐based screening, two *T2TSs* were identified: *AcT2TS1* localizes to a chromosome, whereas *AcT2TS2* maps to contig26 (Figure 6A). Additionally, systematic IPR018333‐based screening enabled the identification of all T2TS‐encoding genes in 11 other sponge species. Phylogenetic and sequence alignment analyses indicated that sponge T2TSs cluster into two major clades, exhibiting distinct divergence in their conserved domain architectures. One clade harbors the DxDD substrate‐binding motif, while the other contains the DCTAE substrate‐binding motif, a signature unique to marine metazoans (Figure 6B; Figures S30 and S31).

For functional validation of T2TSs, we heterologously expressed these enzymes in the lanosterol synthase‐deficient *S. cerevisiae* mutant strain GIL77 [46]. GC‐MS analysis of heterologous fermentation products, benchmarked against a lanosterol standard, identified AcT2TS1 as a functional lanosterol synthase (Figures 6C and S32). In contrast, AcT2TS2 exhibited no detectable lanosterol synthase activity under our experimental conditions. To further resolve the evolutionary divergence between these two T2TS clades, we constructed a phylogenetic tree incorporating representative T2TSs from bacteria, fungi, plants, and all sponge T2TSs identified herein. The results showed that most of the sponge T2TSs form a distinct, monophyletic clade. However, a subset of sponge T2TSs, including AcT2TS2, clustered with bacterial T2TSs (Figure 6D). These results indicate that AcT2TS1 functions as a lanosterol synthase, while AcT2TS2 represents a putative bacterial‐like locus.

To further characterize this locus, we analyzed its genomic context. Within the 15 kb upstream and 96 kb downstream flanking regions of *AcT2TS2*, all adjacent genes are intron‐less, whereas flanking gene *18* contains 2 introns and *41* contains 1 intron, respectively (Figure 6E,F). Notably, a gene immediately upstream of *AcT2TS2* encodes a radical SAM enzyme, a hallmark of bacterial origin, implicated in bacterial membrane biogenesis (Figure 6E; Figures S33 and S34) [47]. GC content analysis of protein‐coding sequences adjacent to *AcT2TS2* yielded values of ∼50% (Figure 6F). AntiSMASH analysis predicted a putative terpenoid biosynthetic gene cluster (BGC) in *A. cavernosa*, encompassing *AcT2TS2* and adjacent modifying enzyme‐encoding genes. Microsynteny analysis of this BGC across the 11 other sponge species revealed that the Kosterfjord Geodia species (*G. barretti*) sponge harbors a homologous gene cluster (Figure 6G). Subsequent BLAST searches of *AcT2TS2* and its flanking genes against the NCBI non‐redundant (nr) database showed that homologous proteins with >40% sequence identity are almost exclusively of bacterial origin; however, some homologous proteins were also found in other sponges (Figure 6H). When we further analyzed the entire contig (contig26) containing *AcT2TS2*, we found that 49 out of the 378 genes on this contig contain 1–4 introns. The homologs of these intron‐containing genes are predominantly from animals, with a smaller fraction from algae and protozoa (Dataset3). GO enrichment analysis of the genes on contig26 showed that they are mainly associated with basal metabolism and membrane (Figure S35). Collectively, these lines of evidence suggest that the genomic region surrounding *AcT2TS2* is of bacterial origin, and that such bacterial‑like loci are not unique to *A. cavernosa*.

### 2.6 Biosynthetic Genes for Terpenoids Produced by T1TSs are not Clustered Into BGCs in *A. cavernosa*

**FIGURE 7:**
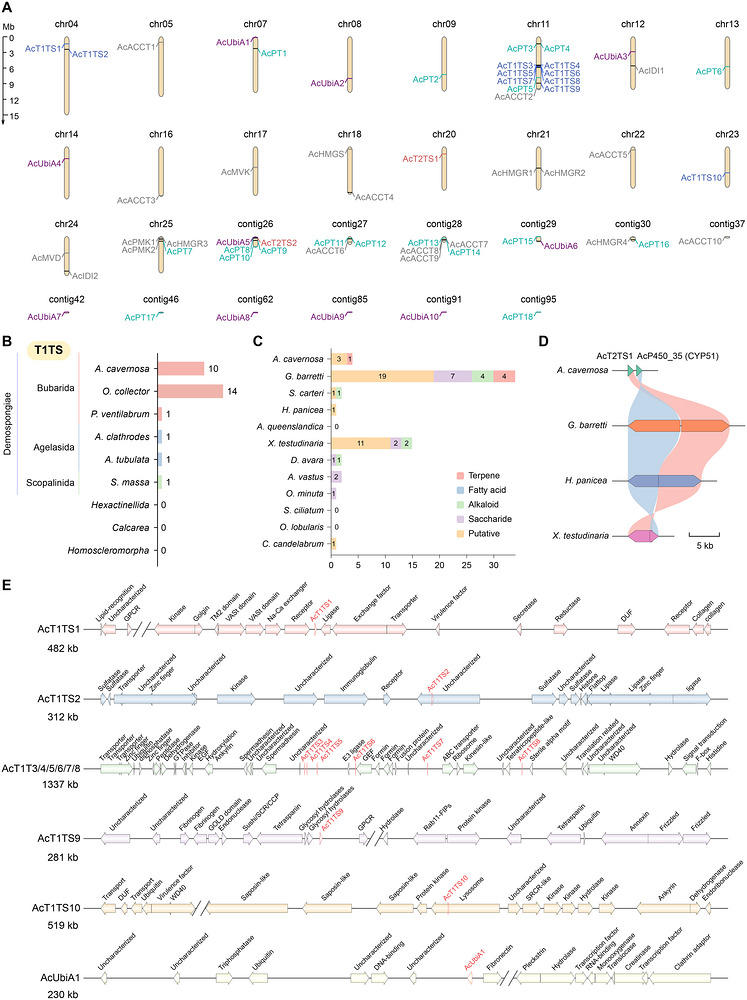
Terpene biosynthetic genes are not clustered in sponges. (A) Chromosomal localization of MEP/MVA pathway genes, *PTs*, *UbiAs*, *T1TSs* and *T2TSs*. (B) Statistics of *T1TSs* reported in sponges to date and those identified in our study. *O. collector*, *P. ventilabrum*, *A. clathrodes*, *A. tubulata*, and *S. massa* stand for *Obruta collector*, *Phakellia ventilabrum*, *Agelas clathrodes*, *Agelas tubulata*, and *Stylissa massa*, respectively. (C) The number of gene clusters in 12 sponges predicted by antiSMASH (Dataset5). (D) Microsynteny analysis of clustered *T2TS* and *P450* (*CYP51*) in *A. cavernosa*, *G. barretti*, *H. panicea*, and *X. testudinaria*. Scale bar, 5 kb. (E) No terpene‐modifying genes were identified in the chromosomal vicinity of either *T1TSs* in *A. cavernosa* or *AUbiA1*. Shown are the 10 genes flanking each terpene synthase.

Gene clusters facilitate the biosynthesis of secondary metabolites, and an increasing number of secondary metabolites from diverse species, including octocorals [17, 22], are produced via BGCs. When analyzing the chromosomal distribution of genes involved in the MVA/MEP pathways, *PTs*, *UbiAs*, *T1TSs*, and *T2TSs* in *A. cavernosa*, we found that aside from a cluster of seven *T1TSs* on one chromosome, no other multi‐gene biosynthetic clusters were identified (Figure 7A). Further examination of the conservation of this *T1TS* cluster across other sponge species revealed no similar clustering in any of these taxa. For instance, *O. collector* harbors 14 *T1TSs* [12], but these are not clustered; other sponge species contain at most one *T1TS*. Notably, *T1TSs* have been exclusively documented in demosponges (Class Demospongiae), with no records in glass sponges (Hexactinellida), calcareous sponges (Calcarea), or homoscleromorph sponges (Homoscleromorpha) (Figure 7B).

We further analyzed BGCs in *A. cavernosa* and the 11 additional sponge species using antiSMASH. While a substantial number of BGCs were predicted across these sponges, only five were associated with terpenoid biosynthesis–‐and all five were linked to *T2TSs*. Among these, the terpenoid BGC in *A. cavernosa* corresponds to the gene cluster containing *AcT2TS2* (Figures 7C and S36).

Beyond antiSMASH‐predicted clusters, we conducted complementary manual curation of terpenoid‐associated BGCs. Adjacent to *AcT2TS1*, functionally validated as a lanosterol synthase, we identified a *CYP51* (Sterol 14α‐demethylase) family *P450* (*AcP450_35*), which catalyzes the conversion of lanosterol to cholesterol. Microsynteny analysis revealed conserved colocalization of these two genes in *G. barretti*, *H. panicea*, and *X. testudinaria* (Figure 7D). In contrast, further analysis of the 10 genes flanking the *T1TSs* in *A. cavernosa* cluster locus revealed no associated modifying genes in their vicinity (Figure 7E). These findings further demonstrate that the biosynthetic genes for terpenoids produced by T1TSs are not organized into clusters in *A. cavernosa*.

## 3 Discussion

**FIGURE 8:**
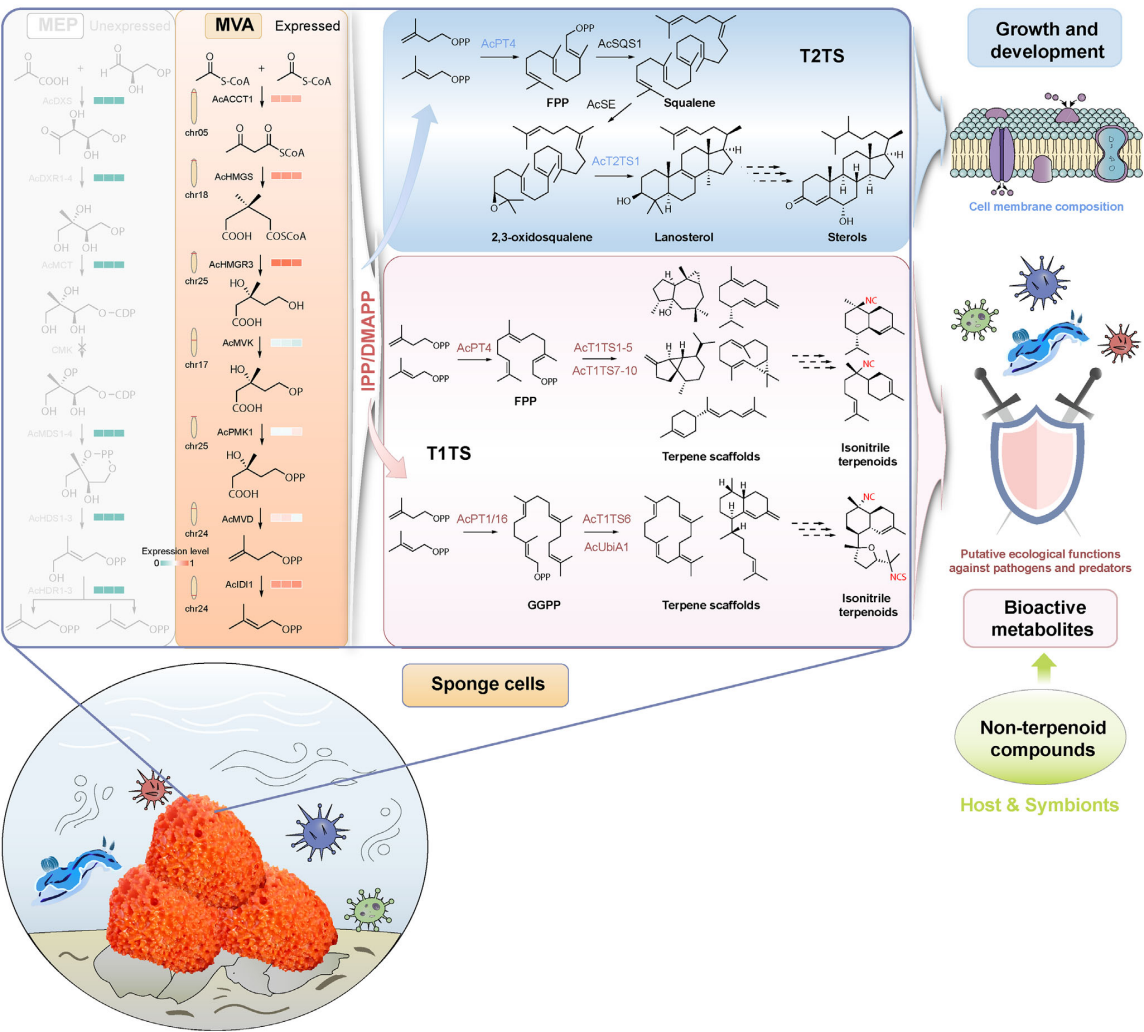
The model of evolved genetic and biosynthetic basis for terpenoid biosynthesis in *A. cavernosa* sponges.

Our findings indicate that marine sponges evolutionarily conserve the MVA pathway and utilize terpene biosynthetic genes, encompassing single α‐domain classical and UbiA‐type noncanonical TSs, to produce terpenoids (Figure 8). These results are consistent with the emerging view that complex metabolic traits may have deep evolutionary origins, and they lend support to the hypothesis that the capacity for chemical‐mediated biotic interactions represents an ancestral feature encoded in early animal genomes. Importantly, we acknowledge that our study constitutes the second report of T1TSs in sponges, building upon the pioneering work by Moore and colleagues on a related Bubarida species [12]. Our high‐quality genome assembly not only corroborates but also extends those earlier findings. Furthermore, our genome‐wide analysis of *A. cavernosa* reveals an absence of clustering among terpenoid biosynthetic genes, providing an additional example of this genomic arrangement in sponges. This observation, while based on a limited number of examples, suggests that early animals may have employed a flexible, decentralized genetic framework for secondary metabolism, one that predates the metabolic specialization and cluster‐based organization characteristic of some later‐diverging lineages.

As sessile filter‐feeders devoid of innate immunity, spongesd rely on secondary metabolites to navigate biotic interactions, which are often assumed to play protective roles. Prior studies have established that sponge‐associated symbionts contribute significantly to the production of bioactive metabolites [48], and the host itself is also known to synthesize certain bioactive compounds such as peptides [49] and sitosterol (Figure S37) [50]. Building on this foundation, our study contributes to the field by providing additional evidence for the host's inherent and autonomous metabolic capacity. Specifically, we show that sponges themselves encode a diverse repertoire of TSs, enabling the synthesis of structurally distinct terpene scaffolds (e.g., germacrene D, *β*‐cubebene) (Figure 8), and that the host genome encodes enzymes that synthesize terpene scaffolds, which may serve as precursors to many signature nitrogen‐containing terpenoids, including isonitrile derivatives central to putative ecological roles in Bubarida sponges [15], rather than these fully elaborated nitrogenous compounds being produced exclusively by symbiotic microbes. The integration of host‐encoded biosynthetic pathways with symbiont‐derived chemistry represents a widespread ecological strategy in metazoans [51, 52, 53]. Our study now extends this paradigm to early‐branching animals, demonstrating that sponges, a basal metazoan lineage, have retained an ancestral capacity for maintaining the ability to leverage symbiotic contributions, while simultaneously autonomously biosynthesizing complex terpenoids with proposed ecological functions (Figure 8). This reframing of sponges as autonomous metabolic agents underscores terpenoids as linchpins of their ecological success, mediating not only predator‑prey interactions and antimicrobial activity but also potential roles in spatial competition and microbiome modulation, processes that shape benthic marine ecosystems.

Our finding that sponge T1TSs possess a single α‐domain, with phylogenetic affinity closer to plant enzymes than to fungal or other animal orthologs, is consistent with an ancient, independent origin of metazoan terpene cyclization activity. This structurally minimal α‐domain form may represent the evolutionary precursor from which more complex, multi‐domain TSs diversified in later animal lineages. The retention of this ancestral architecture in sponges, which are among the earliest‐branching metazoans, suggests that the α‐domain alone is sufficient to catalyze terpene cyclization, and that the acquisition of additional β/γ domains in other eukaryotes occurred later and likely conferred enhanced catalytic efficiency or regulatory complexity rather than enabling a fundamentally new function.

During long‐term evolution, sponges have formed mutualistic symbioses with bacteria, algae, and protozoa, making it challenging to fully disentangle host and symbiont genomes. Our findings show that contig26 is a complex recombinant region containing genes of bacterial, algal, and animal origin (Dataset3). This region may represent a fragment acquired from symbionts and subsequently recombined into a structure with membrane‐associated, organelle‐like properties, or it may exist as a membraneless, single contig entity with basic metabolic functions. Alternatively, we cannot rule out the possibility of symbiont contamination. The high reproducibility of homologous genes in *G. barretti* supports the authenticity of this type of chimeric region. Such a chimeric state might represent an early stage in the evolution of short chromosomes in sponges (a transitional state before the completion of genetic integration) or even a possible origin of animal organelles.

Despite these advances, critical gaps remain. First, we did not identify the genes responsible for nitrogen incorporation (e.g., isonitrile synthase, isothiocyanate synthase, or other nitrogen‐tailoring enzymes). Whether the final nitrogen‑containing compounds are assembled solely by host enzymes, solely by symbionts, or through a combination of both remains unknown. Future studies will need to combine isotope‑feeding experiments, spatial transcriptomics, and native enzyme characterization to resolve this question. Second, the regulatory logic governing unclustered, genomically dispersed terpene metabolism remains elusive: how do sponges coordinate the expression of scattered biosynthetic genes to mount timely and context‑appropriate chemical responses? Third, the full suite of endogenous terpenoids produced by *A. cavernosa* has yet to be completely isolated and structurally characterized. Definitive structural assignment of the sponge's natural products will require further investigation with additional biological material. Furthermore, whether the terpenoids identified in the sponge extract, as well as the products generated by the T1TSs characterized in this study, possess ecological functions requires future investigation. Linking these compounds to ecological activities will require in situ metabolomics and functional ecological assays, such as feeding deterrence experiments and microbiome modulation studies.

## 4 Conclusions

In conclusion, our findings provide additional evidence that terpenoid biosynthesis has deep evolutionary roots in animals, supporting the presence of this metabolic capacity in early‐branching metazoans. Beyond evolutionary biology, this study highlights sponges as valuable sources of biochemical diversity, with implications for natural product discovery, particularly for characterizing enzymes and metabolic strategies that could be harnessed for biotechnological applications. We acknowledge that the endogenous terpenoid structures await direct validation due to limited biomass. Nonetheless, future research addressing the unresolved questions outlined here will continue to refine our understanding of early animal metabolism and further position sponges as key mediators of marine ecosystem function and as potential sources of therapeutic and industrial compounds.

## 5 Methods

### 5.1 Sample Collection

*A. cavernosa* samples were collected at 5 m depth from the South China Sea (18°14′00′′N 109°23′10′′E) in November 2023 (Figure S2). After collection, the samples were washed thoroughly to remove surface impurities, including symbiotic algae and other contaminants, as much as possible. Then the samples were cut into smaller sections and immediately frozen in liquid nitrogen, and subsequently stored at ‐80°C until used for metabolite extraction, DNA or RNA extraction, and subsequently high‐throughput sequencing.

### 5.2 UPLC‐HRMS Analysis of the Total Compounds from *A. cavernosa*

Frozen tissues of *A. cavernosa* were ground into a fine powder under liquid nitrogen using a mortar and pestle. For each sample, 300 mg of the powder was mixed with 300 µL of acetone, vigorously vortexed, and ultrasonicated for 15 min. The mixture was then centrifuged at 13,000 rpm for 10 min, and the supernatant was collected and evaporated to dryness under reduced pressure. The residue was reconstituted in an equal volume of acetonitrile (250 µL) and filtered through a 0.22 µm membrane filter prior to UPLC‐HRMS analysis.

UPLC‐HRMS analysis was performed on an Agilent 6545 Q‐TOF mass spectrometer coupled with an Agilent 1290 Infinity UPLC system. Separation was achieved on a TechMate C18‐ST column (250 × 4.6 mm, 5 µm) maintained at 35°C. The mobile phase consisted of (A) water containing 0.1% formic acid and (B) acetonitrile containing 0.1% formic acid. The gradient program was set as follows: 5% B to 95% B over 15 min, held at 95% B for 15 min, with a total run time of 30 min. The flow rate was 1.0 mL/min, and the injection volume was 20 µL. Diode array detection (DAD) was performed at 210 nm. Mass spectrometry was operated in positive electrospray ionization (ESI) mode with a scan range of *m/z* 100–1,700. The fragmentor voltage was set to 135 V. The drying gas (N_2_) temperature was 300°C at a flow rate of 9 L/min; the nebulizer pressure was 45 psig; the sheath gas temperature was 325°C at a flow rate of 11 L/min; the capillary voltage was 3,500 V.

### 5.3 DNA and RNA Extraction and Genome Sequencing

For DNBSEQ sequencing: 100 mg of sponge tissues were used to extract sufficient DNA using the cetyl trimethylammonium bromide (CTAB) method, following the manufacturer's protocol for subsequent next‐generation sequencing (NGS). Qualified DNA, quantified using Qubit (Invitrogen, Carlsbad, California, USA), was then used to generate the sequencing library, and an index was added to each sample's sequence. After fragmentation, the addition of an A tail, and filtering, a library with insert sizes of 300–500 bp for paired‐end reads was constructed and sequenced using DNBSEQ‐T7 (MGI, Shenzhen, China).

For PacBio Revio sequencing: high‐quality DNA was extracted using QIAGEN Genomic kits (cat. no. 13362). After quality inspection with a NanoDrop 2000 Spectrophotometer (Thermo Fisher Scientific), 20–40 kb fragment DNA was enriched, damage repaired, end‐repaired, and ligated with known adapters to construct an SMRTbell library. Qualified libraries, assessed using an Agilent 2100 Bioanalyzer (Agilent Technologies, USA), were then loaded onto the sequencing chip and sequenced using the Circular Consensus Sequence (CCS) model.

For Chromosome Conformation Capture (Hi‐C) sequencing: nuclei isolated from tissues were fixed in a 1% formaldehyde solution in MS buffer and digested with a restriction enzyme. Biotin labeling was performed, followed by DNA cyclization using T4 DNA ligase (NEB) and DNA purification. Hi‐C libraries were constructed and sequenced using the MGISEQ2000 platform (BGI, Shenzhen, China) with a paired‐end 150 bp read strategy.

For RNA sequencing: total RNA was extracted using TRIzol (Invitrogen, Carlsbad, California, USA) according to the manufacturer's instructions. Library preparation was then performed using the Optimal Dual‐mode mRNA Library Prep Kit (BGI, Shenzhen, China) after RNA quality control passed. DNA nanoballs (DNBs), which contain more than 300 copies of the initial single‐stranded circularized library molecule, were loaded onto the DNBSEQ‐T7 platform chips for sequencing.

### 5.4 Genome Survey

A total of 20 Gb of clean data were retained after filtering with SOAPnuke (v2.2.1) (‐n 0.001 ‐l 10 ‐q 0.5 ‐Q 2) [54]. The genome size, repeat content, and heterozygosity of *A. cavernosa* were estimated using Jellyfish (v2.3.0) [55] and GenomeScope2 [56] with a 17‐kmer.

### 5.5 Genome Assembly

The subreads produced by the PacBio Revio platform were processed using CCS (v6.4.0) [57] (https://github.com/PacificBiosciences/ccs) with the following parameters “–min‐passes 3 –min‐rq 0.99 –min‐length 500”. Nextdenovo (v2.5.2) [58] was performed to produce the contig‐level assembly. To remove potential contamination, the assembled contigs were taxonomically classified using MMseqs2 [59] against the NR database (release 2024‐02‐08) [60], and contigs were retained only if annotated as eukaryotic and supported by alignment of second‐generation transcriptome data mapped with HISAT2 (v2.2.1) [61]. Hi‐C reads were filtered using SOAPnuke (v2.2.1) [54] with the following parameters: ‐l 15 ‐q 0.2 ‐n 0.05 to remove low‐quality sequences, reads containing N bases, and adapter contamination. The cleaned Hi‐C reads were mapped to the contigs, and the valid read pairs were subsequently used to cluster, order, and orient the contigs into pseudochromosomes using Juicer (v1.5) [62] and 3D‐DNA (v180922) [63]. For genome completeness, BUSCO (v5.4.5) [64] with the metazoa_odb10 lineage consisting of 954 genes was employed to investigate the proportion of complete conserved genes present.

### 5.6 Gene Annotation

For TEs annotation: homology‐based strategy and de novo approaches were used approaches. The repetitive elements in the *A. cavernosa* genome were annotated using the homology‐based approach using RepeatMasker (v4.1.4) [65] and RepeatProteinMask (v4.1.4) [65] with the Repbase database [66]. For de novo methods, RepeatModeler (v2.0.4) [67] and LTR_FINDER (v 1.0.6) [68] were used to create a new data library, and RepeatMasker (v4.1.4) [69] was used to predict repeat sequences against the library.

For gene structure analysis: three methods–‐de novo prediction, homology‐based prediction, and RNA sequencing‐based prediction, were integrated to achieve a comprehensive annotation. For the transcriptome‐based annotation, a total of 6 Gb of raw data per sample were generated and filtered using SOAPnuke (v2.2.1) with the following parameters: (‐l 15 ‐q 0.2 ‐n 0.05) [57] to obtain clean data for further gene differential expression analysis. The data were aligned to the *A. cavernosa* assembly with Hisat2 (v2.2.1) [61], and the transcript sequences were assembled using Stringtie (v2.2.1) [69] with the aligned reads. For the homolog‐based annotation, the protein sequences of seven species (*Amphimedon queenslandica*, *Geodia barretti*, *Tethya minuta*, *Ephydatia muelleri*, *Halichondria panicea*, *Tethya wilhelma*, *Xestospongia testudinaria*) were downloaded from each corresponding source (Table S12), and then were mapped to *A. cavernosa* genome using Exonerate (v2.4.0) [70] with the following parameters: “‐model protein2genome –showalignment false ‐showtargetgff true ‐percent 30 ‐bestn 10 –minintron 10 –maxintron 130000”. For de novo prediction, 2000 full‐length genes with intact structure, including start codon, stop codon, and perfect intron‐exon boundary, were selected from homology prediction to train the model parameters for AUGUSTUS (v3.5.0) [71] and SNAP (2006‐07‐28) [72]. Finally, the protein‐coding genes of *A. cavernosa* were predicted using MAKER‐P (v2.31.11) [73], which integrates ab initio prediction with transcriptome and homologous evidence. The completeness of the *A. cavernosa* genome annotation was also evaluated using BUSCO (v5.4.5) [64] with the metazoa_odb10.

The protein‐coding genes of *A. cavernosa* were aligned to Swissprot (release 2024‐03‐27), Trembl (release 2024‐03‐27) and NR (release 2024‐02‐08) using diamond (v2.0.14) [74] with an E‐value threshold of 1e‐5, while EggNOG‐mapper (v2.0) [75] was used to perform KEGG annotation with emapperdb (v5.0.2). InterProScan (v5.71‐102.0) [76] was employed to identify the conservation domain based on the public databases SUPERFAMILY, NCBIFAM, PRINTS, Pfam, SMART, ProSiteProfiles, and ProSitePatterns.

### 5.7 Comparative Genomics Analyses

Protein sequences of 12 sponges were downloaded from online database (Table S12), excluding the one genome assembled in this study. Gene families were clustered using OrthoFinder (v2.3.3). Single‐copy orthologous genes were aligned with MUSCLE (v3.8.31), and conserved blocks were selected with Gblock (v0.91b). A concatenated alignment was then generated. A maximum‐likelihood phylogenetic tree was constructed using RAxML (v8.2.1) under the PROTGAMMAJTT model with 1000 bootstrap replicates. Divergence times were estimated using the Codeml and MCMCtree programs in the PAML (v4.9) package, with time calibrations obtained from the TimeTree database. Based on the gene family clustering results from OrthoFinder and the species phylogeny, gene family expansions and contractions were inferred using CAFÉ (v4.2.1). A random birth and death process was used to model gene loss and gain along every lineage in the phylogenetic tree. Gene families with a *p*‐value less than 0.05 were considered as significantly expanded or contracted families.

### 5.8 Terpenoids Related Genes Discovery

InterProScan and Pfam annotation information were used to find terpenoid biosynthesis‐related genes. For MEP pathway genes, IPR005477 and PF13292 were used to find 1‐deoxy‐D‐xylulose 5‐phosphate synthase. IPR003821, PF08436, PF02670, and PF13288 were used to find DXP‐reductoisomerase. PF01128 was used to find 2‐C‐methyl‐D‐erythritol 4‐phosphate cytidylyltransferase. IPR004424, PF08544 and PF00288 were used to find 4‐diphosphocytidyl‐2‐C‐methyl‐D‐erythritol kinase. IPR036571 and PF02542 were used to find 2‐C‐methyl‐D‐erythritol 2,4‐cyclodiphosphate synthase. IPR016425, IPR004588 and PF04551 were used to find 4‐ hydroxy‐3‐methylbut‐2‐enyldiphosphate synthase. IPR003451 and PF02401 were used to find 4‐hydroxy‐3‐methylbut‐2‐enyl diphosphate reductase. For MVA pathway genes, IPR002155, PF00108 and PF02803 were used to find acetyl‐coenzyme A (CoA) C‐acetyltransferase. IPR013746, IPR013528, PF01154 and PF08540 were used to find 3‐hydroxy‐3‐methylglutaryl‐CoA (HMG‐CoA) synthase. IPR002202 and PF00368 were used to find HMG‐CoA reductase. PF08544 and PF00288 were used to find mevalonic acid kinase. PF04275 was used to find phosphomevalonate kinase. PF18376 was used to find diphosphomevalonate decarboxylase. IPR011876 and PF00293 were used to find Isopentenyl‐diphosphate Delta‐isomerase. IPR000092, PF00348, IPR036424 and PF01255 were used to find isoprenyl diphosphate synthase. IPR008949, IPR008930, IPR018333, PF19086, PF00494, PF13243 and PF08491 were used to find terpene synthase. IPR036396 and PF00067 were used to find Cytochrome P450.

### 5.9 Instruments and Materials

Gas chromatography‐mass spectrometry (GC‐MS) analyses were conducted using an Agilent 8890 GC system (Agilent Technologies, Shanghai, China) coupled with a 5977C mass spectrometric detector (Agilent Technologies, DE, USA) and a 7650 autosampler (Agilent Technologies, China). Reversed‐phase high‐performance liquid chromatography (RP‐HPLC) was performed on an Agilent 1260 Infinity LC system equipped with an Agilent Zorbax SB‐C18 analytical column (4.6 × 150 mm, 5 µm particle size). Preparative HPLC was carried out using the same system with an Agilent Zorbax SB‐C18 preparative column (21.2 × 250 mm, 7 µm). Nuclear magnetic resonance (NMR) spectra were recorded on a Bruker 600 MHz spectrometer (Bruker Biospin AG, Fällanden, Germany). Column chromatography (CC) was performed using commercial silica gel (200–300 and 300–400 mesh; Yantai Xinnuo Chemical Co., Ltd., Yantai, China). Solvents for CC, HPLC, and GC‐MS were of analytical grade (Shanghai Chemical Reagents Co., Ltd.) and chromatographic grade (Dikma Technologies Inc., CA, USA), respectively. Isoprenol and isopropyl‐*β*‐D‐thiogalactopyranoside (IPTG) were obtained from Sigma‐Aldrich and BBI Life Sciences Corporation, respectively. The TransScript One‐Step gDNA Removal and cDNA Synthesis SuperMix (AT311) and enzymes for cloning were sourced from TransGen Biotech (Shanghai, China).

### 5.10 Functional Characterizations of TSs

The total RNAs of *A. cavernosa* were extracted from the tissues using the TRIzol isolation kit (Invitrogen, Carlsbad, California, USA). A total of 1.5 µg of the total RNAs was used for complementary DNA (cDNA) synthesis with an oligo (dT) primer using a TransScript One‐Step gDNA Removal and cDNA Synthesis SuperMix. The open reading frame (ORF) sequences for all TSs were PCR amplified from the cDNAs of *A. cavernosa* as templates. The amplification primers are listed in Table S19. The PCR products were ligated into the BamHI/HindIII sites of the pET‐28a vector for *E. coli*, or the BamHI/XhoI sites of the PESC‐ura vector for *S. cerevisiae*. For functional characterization in *E. coli*, the resulting pET‐28a recombinant plasmids were co‐transformed with either the CDF‐MKI3 or CDF‐MKI4 vector (two‐step kinase‐based FPP or GGPP overproduction systems, respectively) into BL21Gold (DE3) cells. In *S. cerevisiae*, selected PESC‐ura recombinant plasmids were either: (i) co‐transformed with the PESC‐his‐tHMGR vector into YPH499 cells, or (ii) transformed alone into GIL77 cells. Following fermentation of the recombinant strains, products were analyzed by HPLC and GC‐MS. Fractions containing new peaks were scaled up, purified, and structurally characterized by GC‐MS, 1D NMR, and 2D NMR.

### 5.11 GC‐MS Analysis

GC‐MS analysis was performed using an Agilent 8890 GC system equipped with a 7650 autosampler and coupled to a 5977C mass spectrometer. Separation was achieved using an HP‐5 ms capillary column (30 m × 0.25 mm × 0.25 µm; Agilent Technologies, USA) with helium as the carrier gas (constant flow: 1.0 mL/min). 1 µL sample was injected with a 3:1 split ratio for analysis using the following temperature program: an initial temperature of 50°C, followed by a gradual increase to 300°C at a rate of 10°C/min with a 5‐min hold (total runtime: 30.0 min). The mass spectrometer operated in electron ionization positive mode (70 eV) with ion source, quadrupole, and transfer line temperatures set at 300°C, 150°C, and 300°C, respectively, scanning a mass range of 30–550 *m/z* in full‐scan mode. Data acquisition and analysis were performed using MassHunter software (v10.2, Agilent Technologies) with compound identification against the NIST 23 mass spectral database.

### 5.12 Isolation of Compounds 1–5

The recombinant *E. coli* BL21Gold (DE3) strain harboring both CDF‐MKI3 and pET28a‐AcT1TS plasmids was cultured in 500 mL LB medium supplemented with kanamycin (50 µg/mL) and streptomycin (50 µg/mL) overnight. The overnight culture was subcultured at a 1:100 ratio into fifteen 1‐liter flasks of fresh LB medium. When the culture reached OD_600_ ≈ 2, 0.5 mM IPTG and 1.0 mM isoprenol were added, followed by incubation at 28°C with shaking (220 rpm) for 18 h to induce compound production. Cells were harvested by centrifugation (4,000 rpm, 15 min) and extracted with methanol. The methanol extract was then partitioned with petroleum ether (PE), concentrated under reduced pressure, and subjected to silica gel column chromatography (200‐300 mesh) eluted with PE to obtain compound **1**. Compounds **2**–**5** were isolated following similar procedures with plasmid variations. Final purification of compounds **3**–**5** was achieved by preparative HPLC using an Agilent Zorbax SB‐C18 column (21.2 × 250 mm, 7 µm) with a gradient of acetonitrile/water (5% to 95% CH_3_CN over 18 min, maintained at 95% for 42 min) at 20 mL/min flow rate.

### 5.13 Bioinformatics and Phylogenetic Analysis

BGC prediction was performed using the online platform plantiSMASH version 1.0 with default parameters. BLAST searches were conducted using the NCBI BLASTp online service against the non‐redundant (nr) database, with an e‐value threshold of < 1e^−^^5^, query coverage > 50%, and sequence identity > 40%. Subcellular localization was predicted using the TargetP‐2.0 online tool with the organism group set to “Non‐plant”.

Neighbor‐joining (NJ) phylogenetic trees were constructed using MEGA 7. Sequence alignments were generated with MUSCLE, and branch support was assessed with 1,000 bootstrap replicates. Maximum‐likelihood (ML) trees were built using IQ‐TREE multicore version 2.4.0. Prior to ML tree inference, sequence alignments were performed with MAFFT. The best‐fit substitution models were automatically selected by IQ‐TREE: the Q.pfam+R6 model was used for PTs, Q.pfam+R9 for T1TSs, and Q.pfam+R7 for T2TSs. All ML analyses were conducted with 1000 bootstrap replicates. For seed plant T1TSs, the α‐domain was manually extracted from full‐length sequences before alignment and tree reconstruction.

Gene cluster visualization and final tree rendering were performed using ChiPlot, with subsequent modifications made in Adobe Illustrator.

### 5.14 Compound Structure Elucidation


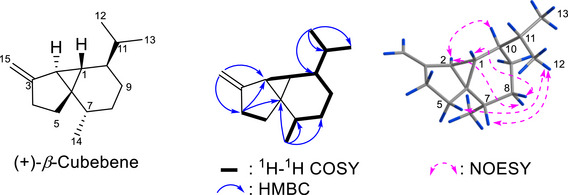


(+)‐*β*‐Cubebene was readily identified by comparing its NMR spectroscopic data with those reported in the literature [77] further confirmed by 2D NMR correlations.

(+)‐*β*‐Cubebene: colorless oil; [*α*]20 D+15.5 (*c* 0.40, CHCl_3_); For ^1^H NMR (CDCl_3_, 600 MHz) and ^13^C NMR (CDCl_3_, 150 MHz) spectral data, see Table S15.


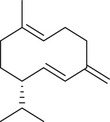


(‐)‐Germacrene D was also identified by comparing its ^1^H and ^13^C NMR spectroscopic data with those reported in the literature [12].

(‐)‐Germacrene D: colorless oil; [*α*]20 D‐134.1 (*c* 0.50, CHCl_3_); ^1^H NMR (CDCl_3_, 600 MHz) *δ* 5.78 (1H, d, *J* = 15.8 Hz, vinyl H), 5.25 (1H, dd, *J* = 15.8, 9.9 Hz, vinyl H), 5.14 (1H, dd, *J* = 11.1, 4.6 Hz, vinyl H), 4.79 (1H, s, vinyl H), 4.75 (1H, s, vinyl H), 0.87 (3H, d, *J* = 6.7 Hz, CH_3_), 0.81 (3H, d, *J* = 6.8 Hz, CH_3_); ^13^C NMR (CDCl_3_, 125 MHz) *δ* 149.04, 135.65, 134.15, 133.71, 129.81, 109.19, 53.07, 40.86, 34.65, 32.90, 29.39, 26.66, 20.91, 19.48, 16.06.


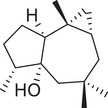


(+)‐isoafricanol was also identified by comparing its ^1^H and ^13^C NMR spectroscopic data with those reported in the literature [78].

(+)‐isoafricanol: colorless oil; [*α*]20 D+16.9 (*c* 0.34, CHCl_3_); ^1^H NMR (C_6_D_6_, 600 MHz) *δ* 1.21 (3H, s, CH_3_), 0.98 (3H, s, CH_3_), 0.81 (3H, d, *J* = 6.3 Hz, CH_3_), 0.80 (3H, s, CH_3_); ^13^C NMR (C_6_D_6_, 125 MHz) *δ* 85.27, 54.58, 46.55, 46.06, 41.27, 34.22, 31.86, 31.68, 31.34, 23.90, 23.03, 22.25, 21.38, 18.85, 12.45.


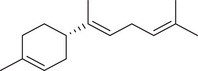


(‐)‐(*E*)‐*α*‐bisabolene was also identified by comparing its ^1^H and ^13^C NMR spectroscopic data with those reported in the literature [79].

(‐)‐(*E*)‐*α*‐bisabolene: colorless oil; [*α*]20 D‐47.5 (*c* 0.40, CHCl_3_); ^1^H NMR (CDCl_3_, 600 MHz) *δ* 5.41 (1H, br s, vinyl H), 5.16 (1H, br t, *J* = 7.2 Hz, vinyl H), 5.12 (1H, br t, *J* = 7.2 Hz, vinyl H), 2.71 (1H, br t, *J* = 7.2 Hz, CH_2_), 1.72 (3H, s, CH_3_), 1.67 (3H, s, CH_3_), 1.65 (3H, s, CH_3_), 1.63 (3H, s, CH_3_); ^13^C NMR (CDCl_3_, 125 MHz) *δ* 139.44, 133.85, 131.43, 123.66, 121.90, 121.06, 42.96, 30.94, 30.91, 28.11, 27.10, 25.86, 23.65, 17.88, 14.29.


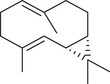


(‐)‐Bicyclogermacrene was also identified by comparing its ^1^H and ^13^C NMR spectroscopic data and optical rotation with those reported in the literature [12].

(‐)‐Bicyclogermacrene: colorless oil; [*α*]20 D‐95.8 (*c* 0.45, CHCl_3_); ^1^H NMR (CDCl_3_, 600 MHz) *δ* 4.86 (1H, dd, *J* = 11.2, 5.2 Hz, vinyl H), 4.37 (1H, d, *J* = 11.5 Hz, vinyl H), 1.69 (3H, s, CH_3_), 1.51 (3H, s, CH_3_), 1.11 (3H, s, CH_3_), 1.05 (3H, s, CH_3_); ^13^C NMR (CDCl_3_, 125 MHz) *δ* 141.01, 128.12, 126.65, 124.96, 41.31, 37.39, 30.20, 29.37, 27.09, 26.97, 26.16, 21.03, 20.02, 16.72, 15.59.

## Author Contributions

**Bao Chen**: software, methodology, formal analysis, funding acquisition, resources, writing – review and editing. **Baofu Xu**: conceptualization, funding acquisition, project administration, supervision, writing – review and editing. **Fangyan Chen**: writing – original draft, investigation, validation, funding acquisition, visualization, writing – review and editing, software. **Guifang Sun**: investigation, validation. **Wenhui Zhang**: investigation, validation. **Jianpeng Zhang**: data curation, methodology. **Yao Zhao**: investigation, validation.

## Funding

This work was supported by the National Key Research and Development Program of China (Young Scientist Project) (2025YFA0924400 to BX, 2025YFA0924000 to BX), the Key R&D Program of Shandong Province, China (2024CXPT029 to BX, 2025CXPT012 to BX), the National Natural Science Foundation of China (82574295 to BX, 82504662 to FC, 22407117 to BC), the Shandong Provincial Natural Science Foundation (ZR2024QB357 to BX, ZR2024QB265 to FC).

## Conflicts of Interest

The authors declare no conflicts of interest.

## Supporting information




**Supporting File 1**: advs77243‐sup‐0001‐SuppMat.xlsx.

**Supporting File 2**: advs77243‐sup‐0002‐SuppMat.pdf.

## Data Availability

The raw sequencing reads and genome assembly are available under NCBI BioProject PRJNA1475267. Transcriptomic data have been deposited under the same BioProject. UPLC‐HRMS, HPLC, and GC‐MS raw files are available from MetaboLights under accession number MTBLS14629. All other data needed to evaluate the conclusions in the paper are present in the paper and/or the Supplementary Materials.
