# Supplementary material for: Investigations of the Evolved Molecular Basis for Terpenoid Biosynthesis in Marine Sponges
Source: Adv Sci (Weinh). 2026 Aug 18:e77243. Online ahead of print. doi: 10.1002/advs.77243 (PMC13483135; doi:10.1002/advs.77243)
Supplement: Supplementary file 1 [file ADVS-9999-e77243-s001.pdf]

Supporting Information

Investigations of the evolved molecular basis for terpenoid biosynthesis in marine sponges

Fangyan Chen[1,2,4], Bao Chen[1,4], Wenhui Zhang[1], Guifang Sun[1,2], Yao Zhao[1], Jianpeng Zhang[3] & Baofu Xu[1,2,]*

[1]Shandong Laboratory of Yantai Drug Discovery, Bohai Rim Advanced Research Institute for Drug Discovery, Yantai, Shandong, 264117, China.

[2]State Key Laboratory of Drug Research, Shanghai Institute of Materia Medica, Chinese Academy of Sciences, 555 Zu Chong Zhi Road, Zhangjiang Hi-Tech Park, Shanghai, 201203, China.

[3]Smartgenomics Technology Institute, Tianjin, 301725, China.

[4]These authors contributed equally: Fangyan Chen, Bao Chen.

E-mail: bfxu@simm.ac.cn (Baofu Xu)

Supplementary Figures

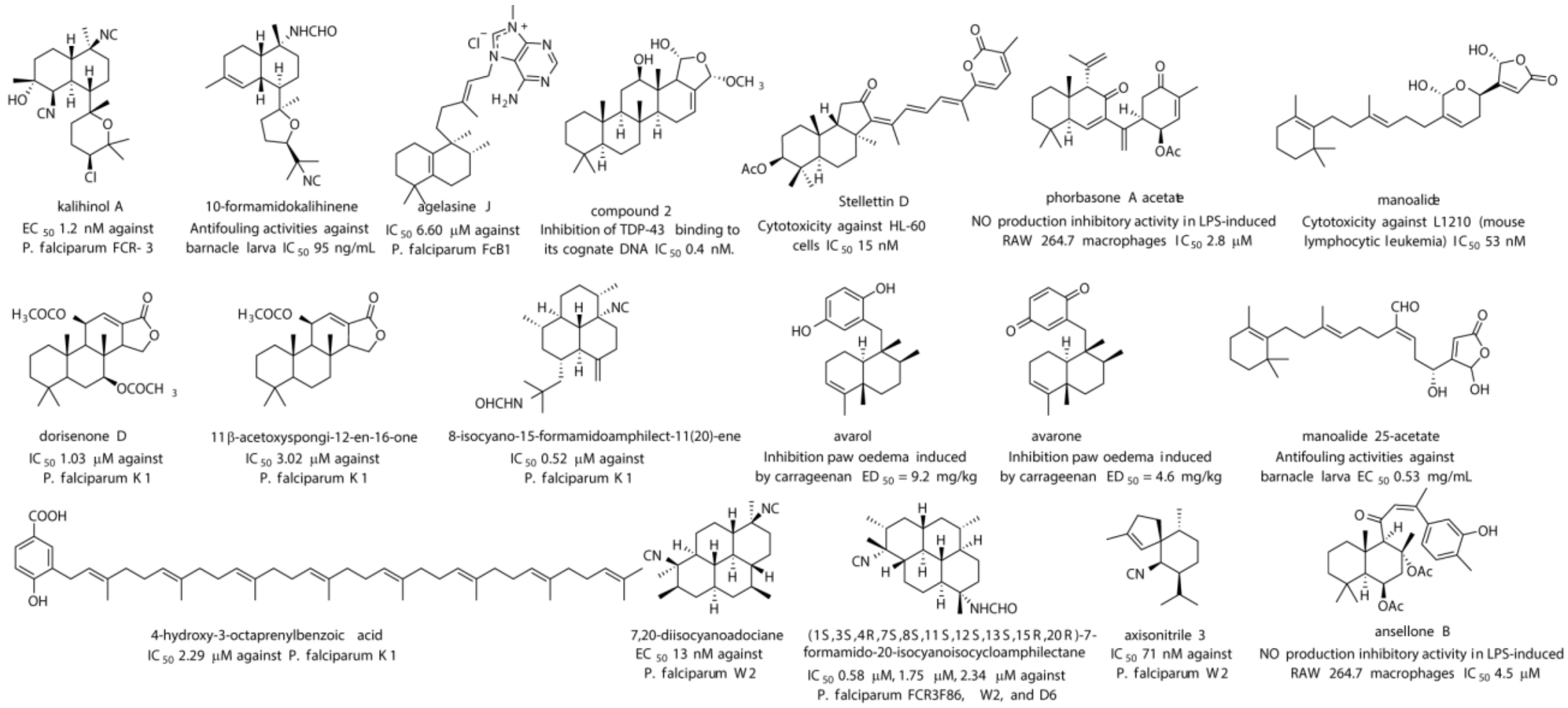

Figure S1. Bioactive terpenoid compounds from sponges.

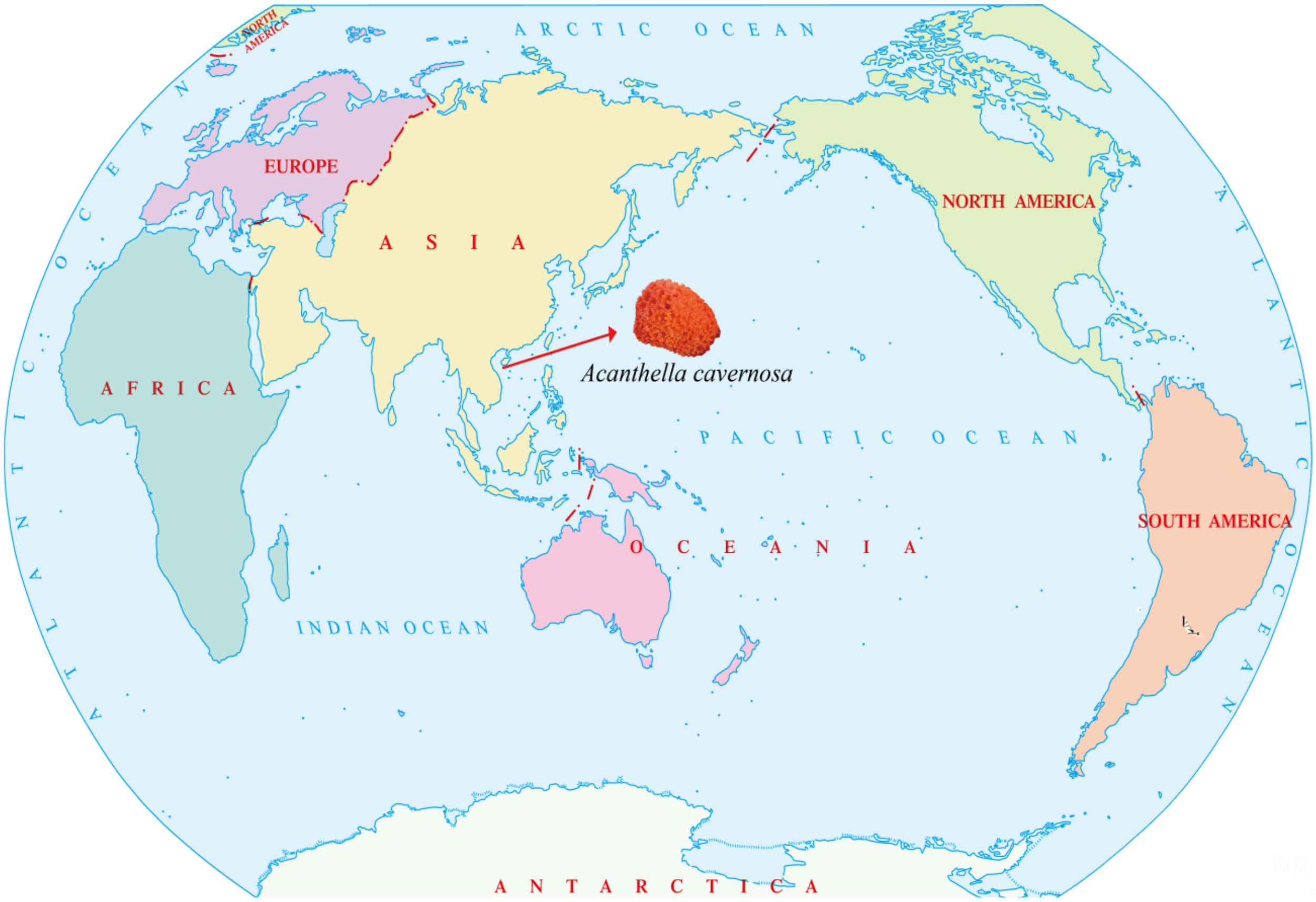

Figure S2. Location of *A. cavernosa* sponge collection (18°14′00″N, 109°23′10″E, 5 m depth, South China Sea, Nov 2023).

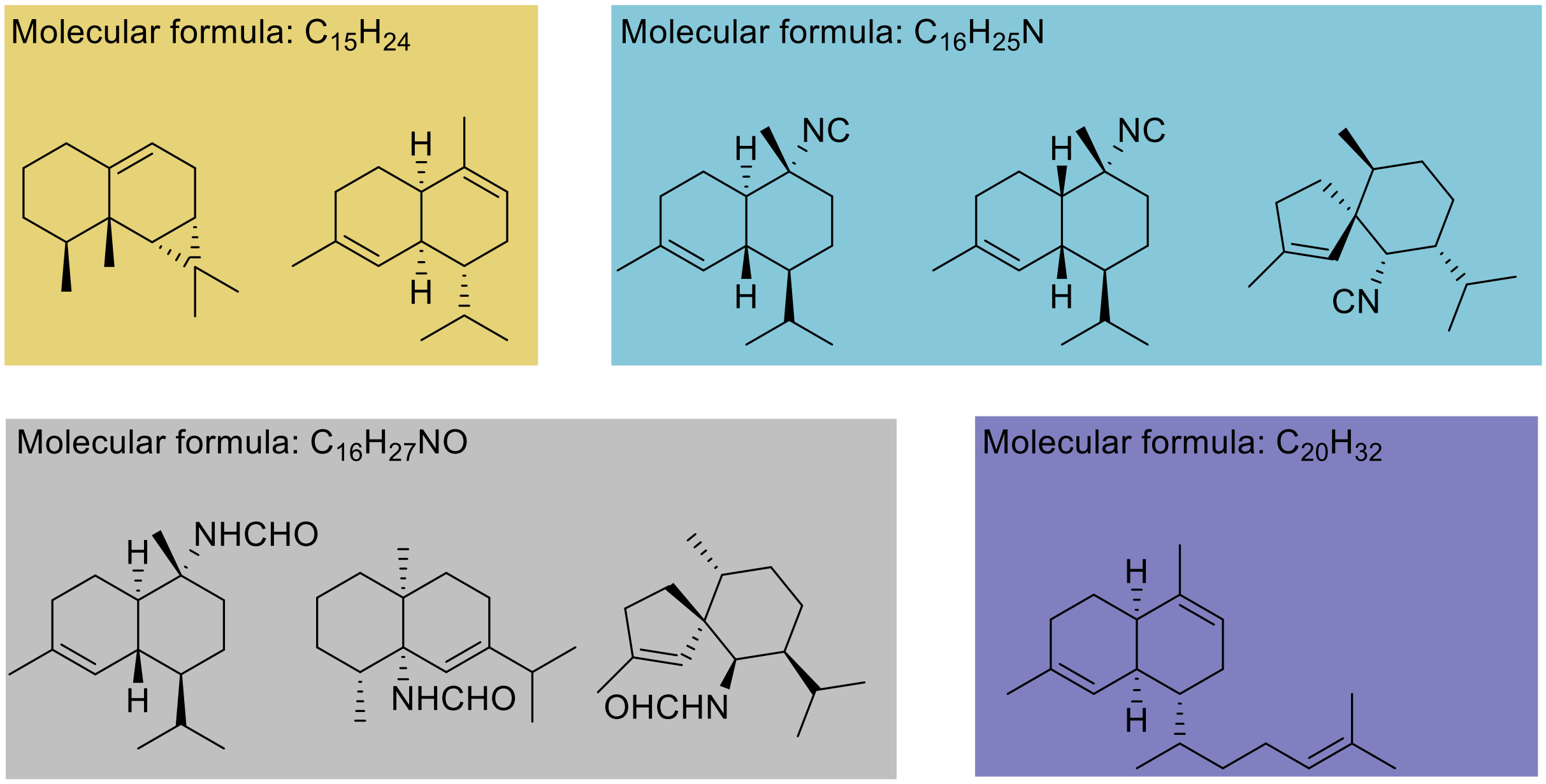

Figure S3. Representative reported compounds isolated from *A. cavernosa* with the molecular formulas $C_{15}H_{24}$ (*m/z* 205.1951), $C_{16}H_{25}N$ (*m/z* 232.2060), $C_{16}H_{27}NO$ (*m/z* 250.2165), and $C_{20}H_{32}$ (*m/z* 273.2577).

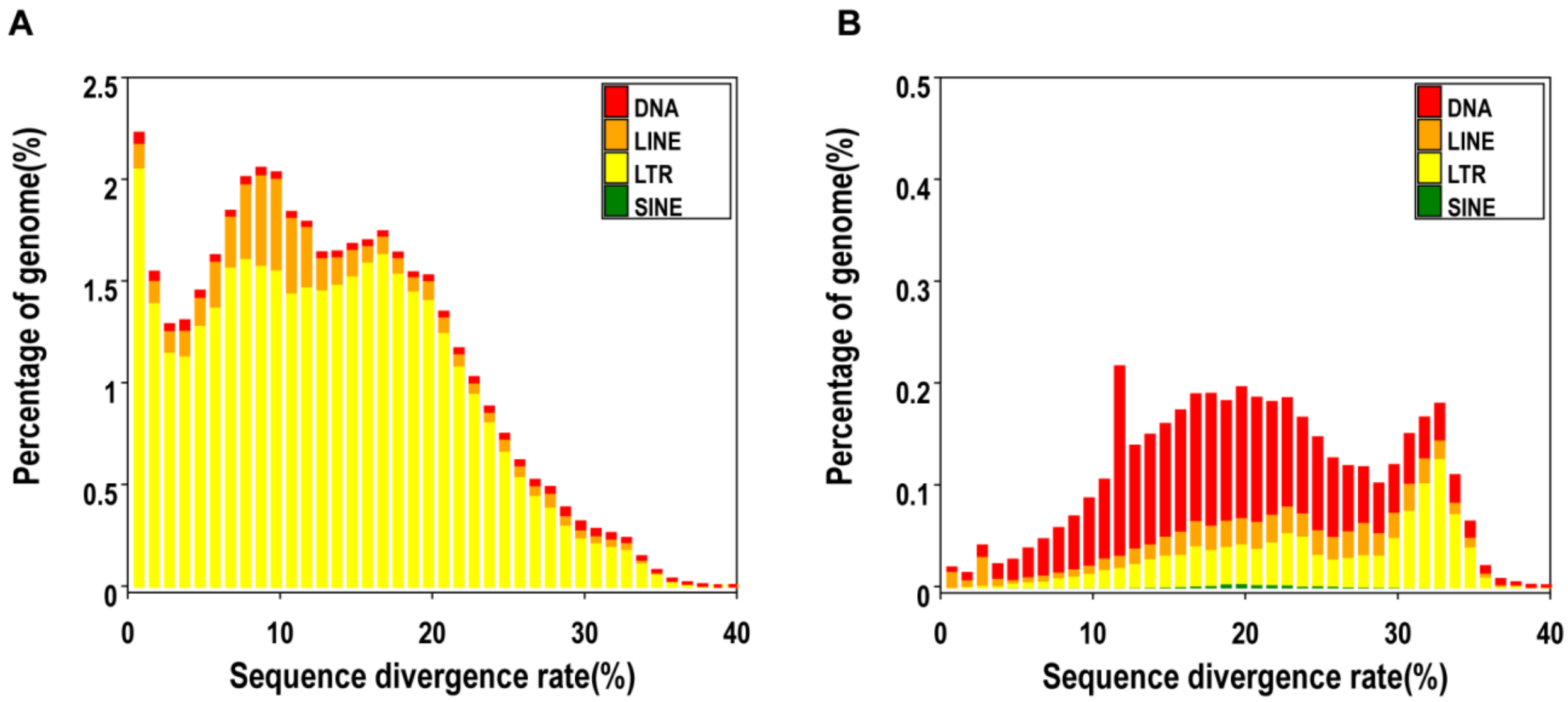

Figure S4. Diverse sets of de novo and known LTR elements in the *A. cavernosa* genomes.

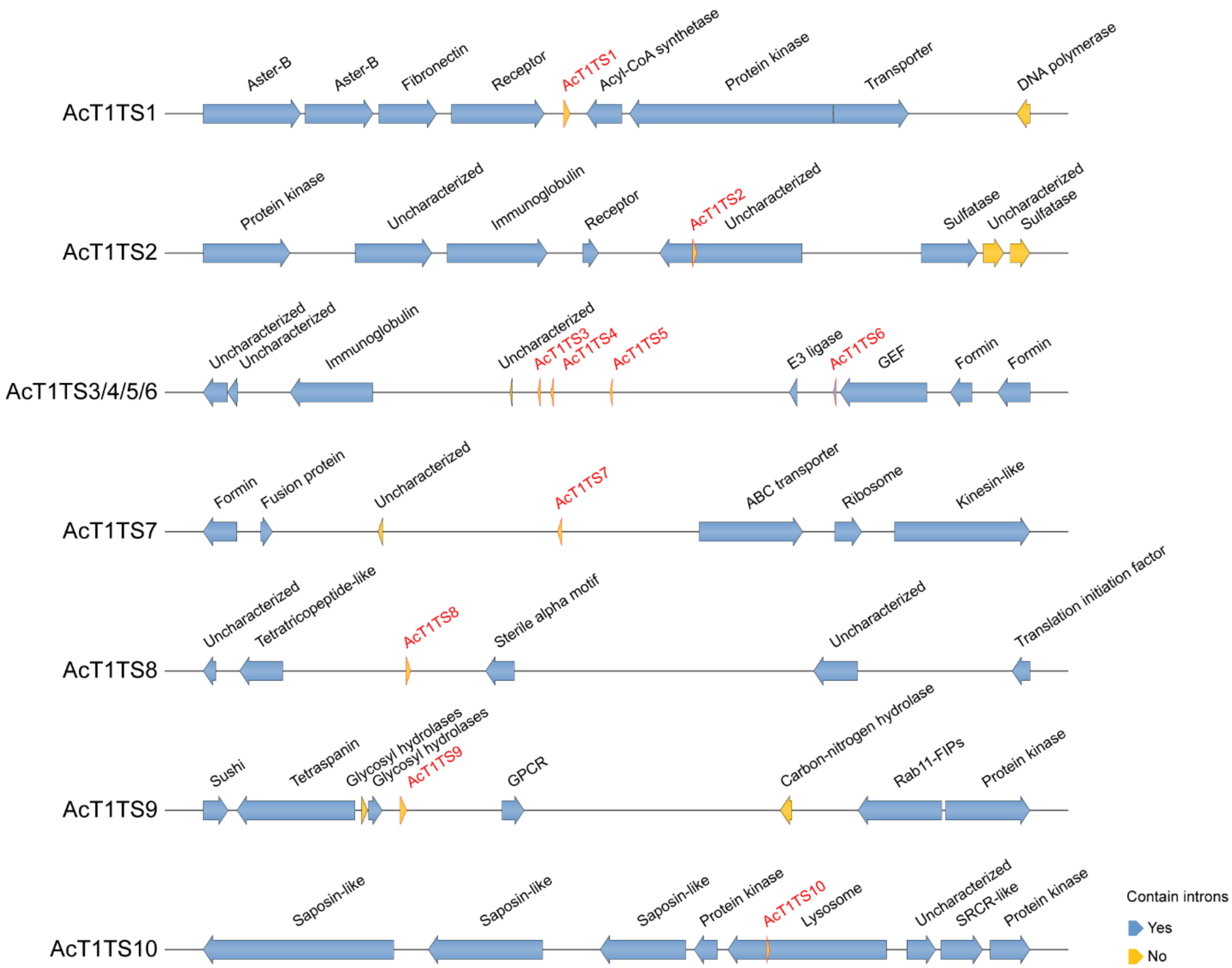

Figure S5. Annotations and intron distributions of genes adjacent to the *ACT1TSs* locus on the chromosome.

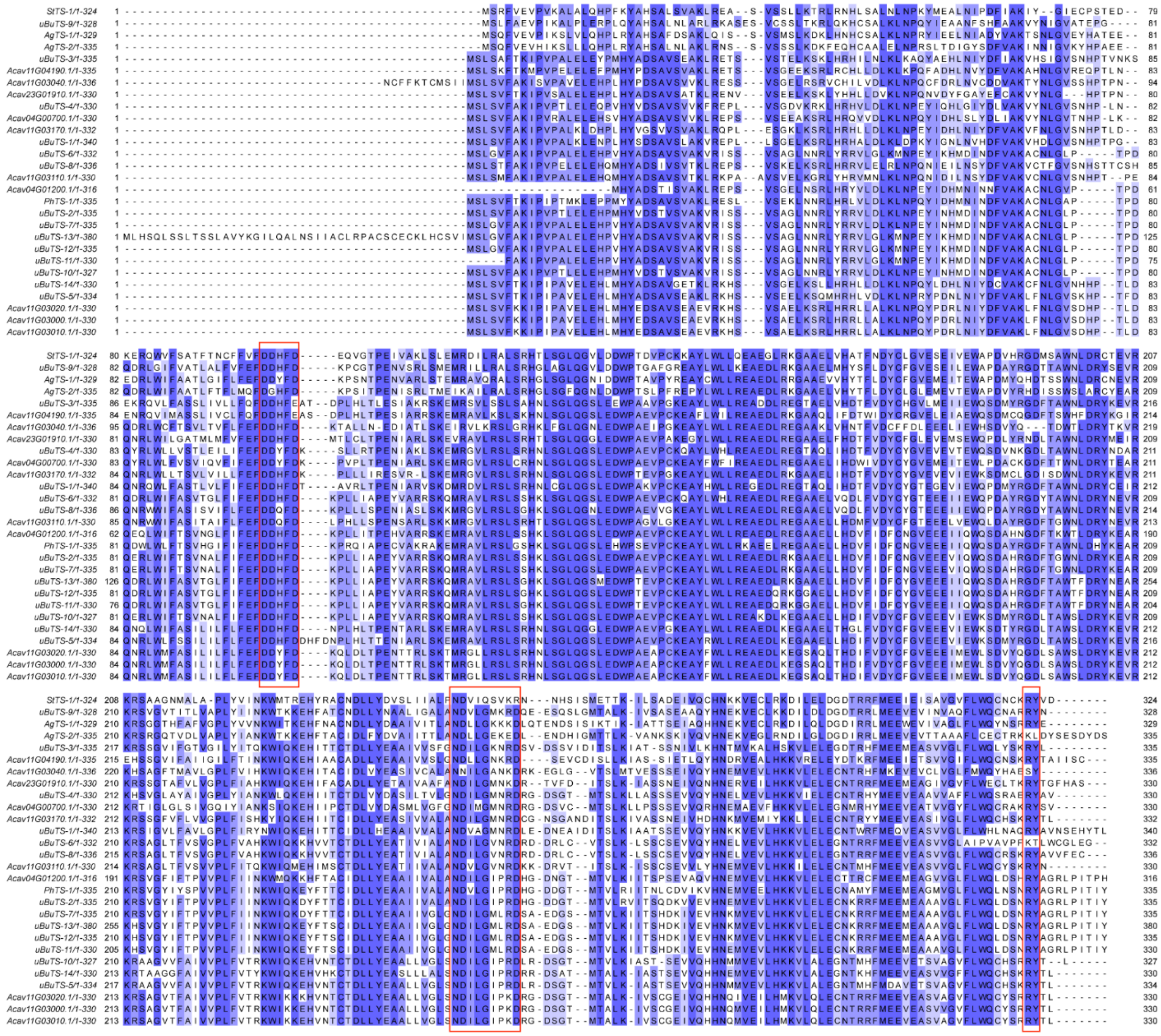

Figure S6. Multiple sequence alignment of sponge T1TSs.

Original experimental spectra of the product of AcT1TS1: (+)-isoafricanol (1).

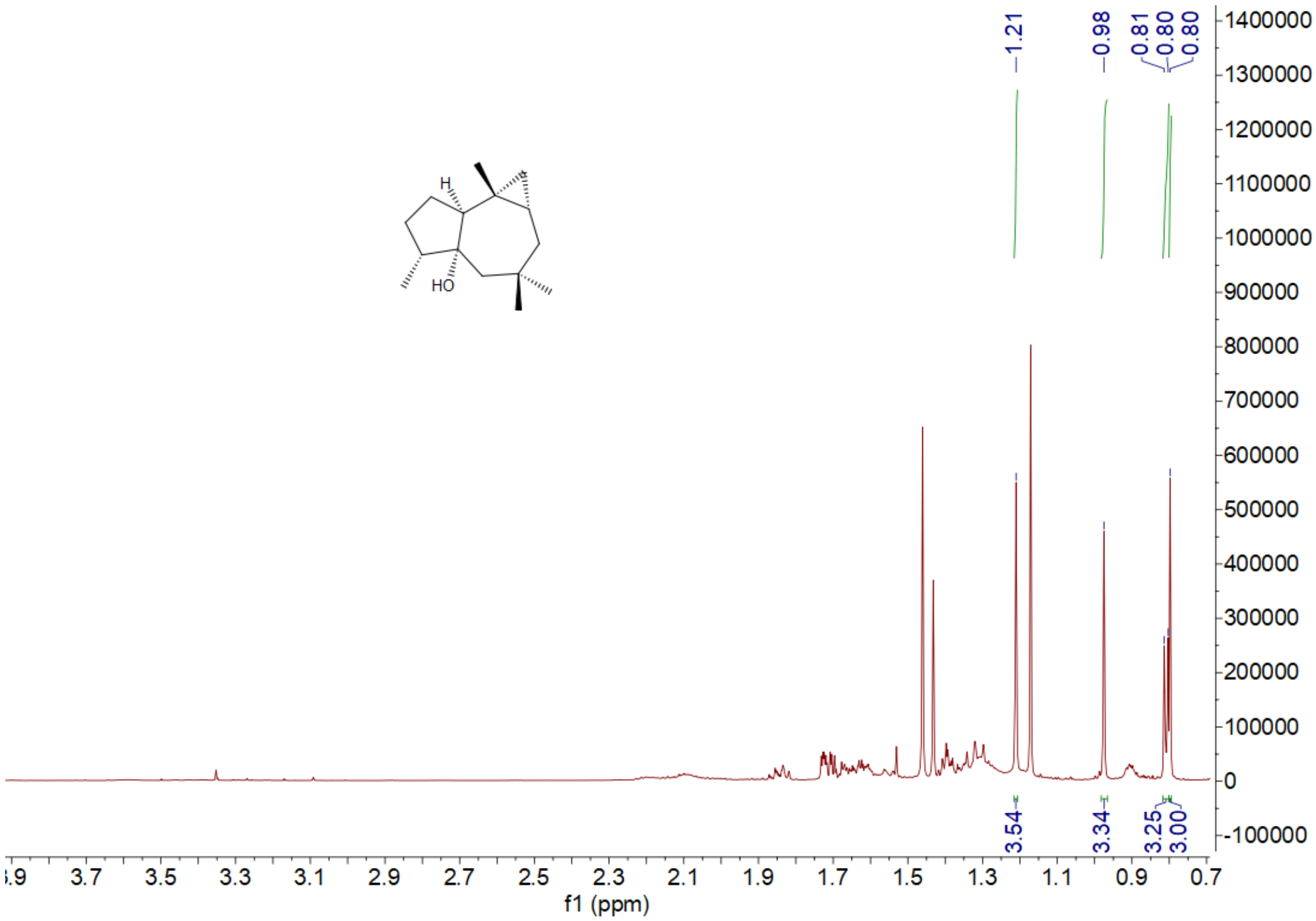

Figure S7. ^{1}H NMR spectrum of (+)-isoafricanol in C_6D_6.

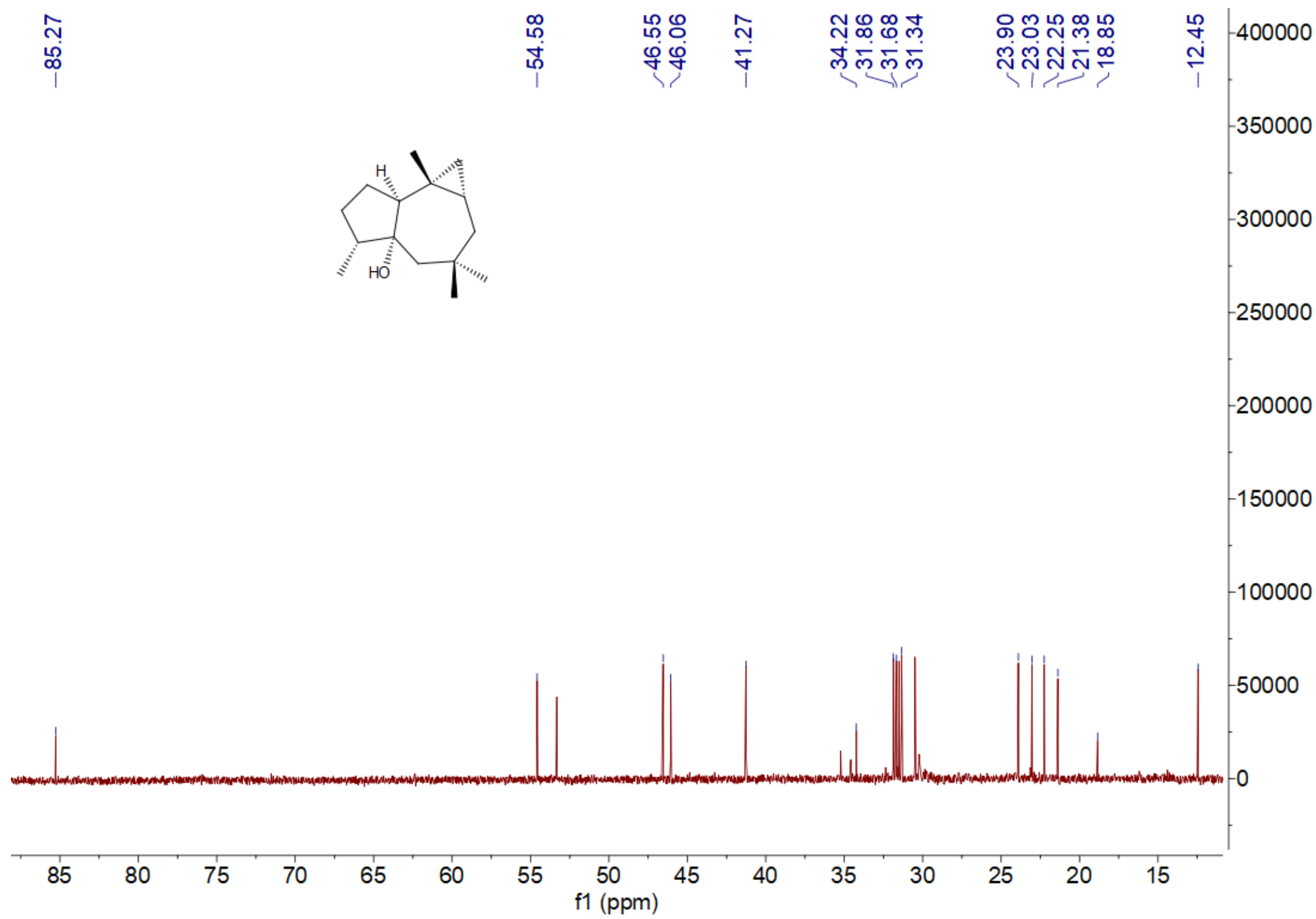

Figure S8. ^{13}C NMR spectrum of (+)-isoafricanol in C_6D_6.

Original experimental spectra of the product of AcT1TS2 and AcT1TS7: (-)-Germacrene D (2).

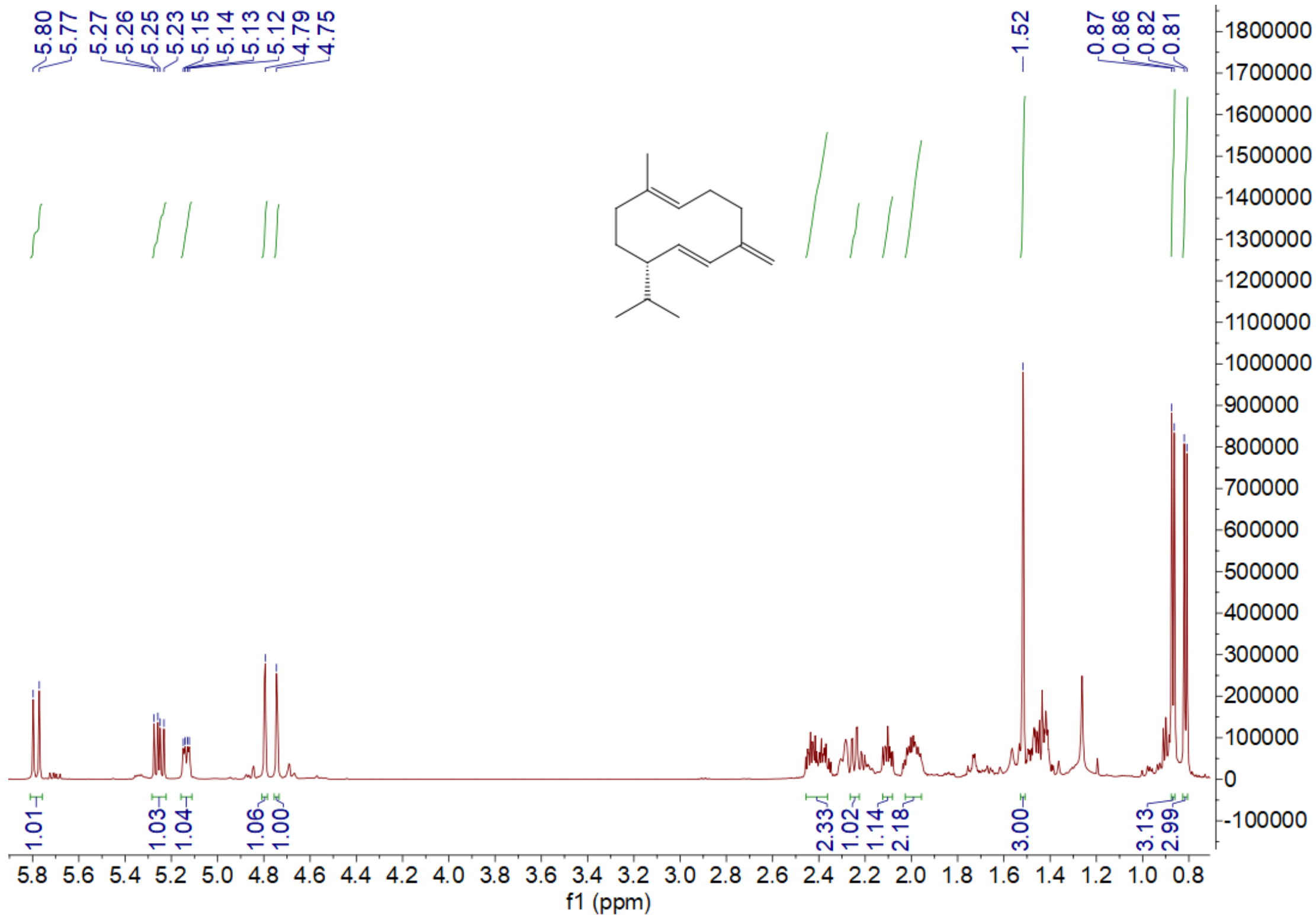

Figure S9. ^{1}H NMR spectrum of (-)-Germacrene D in $CDCl_3$.

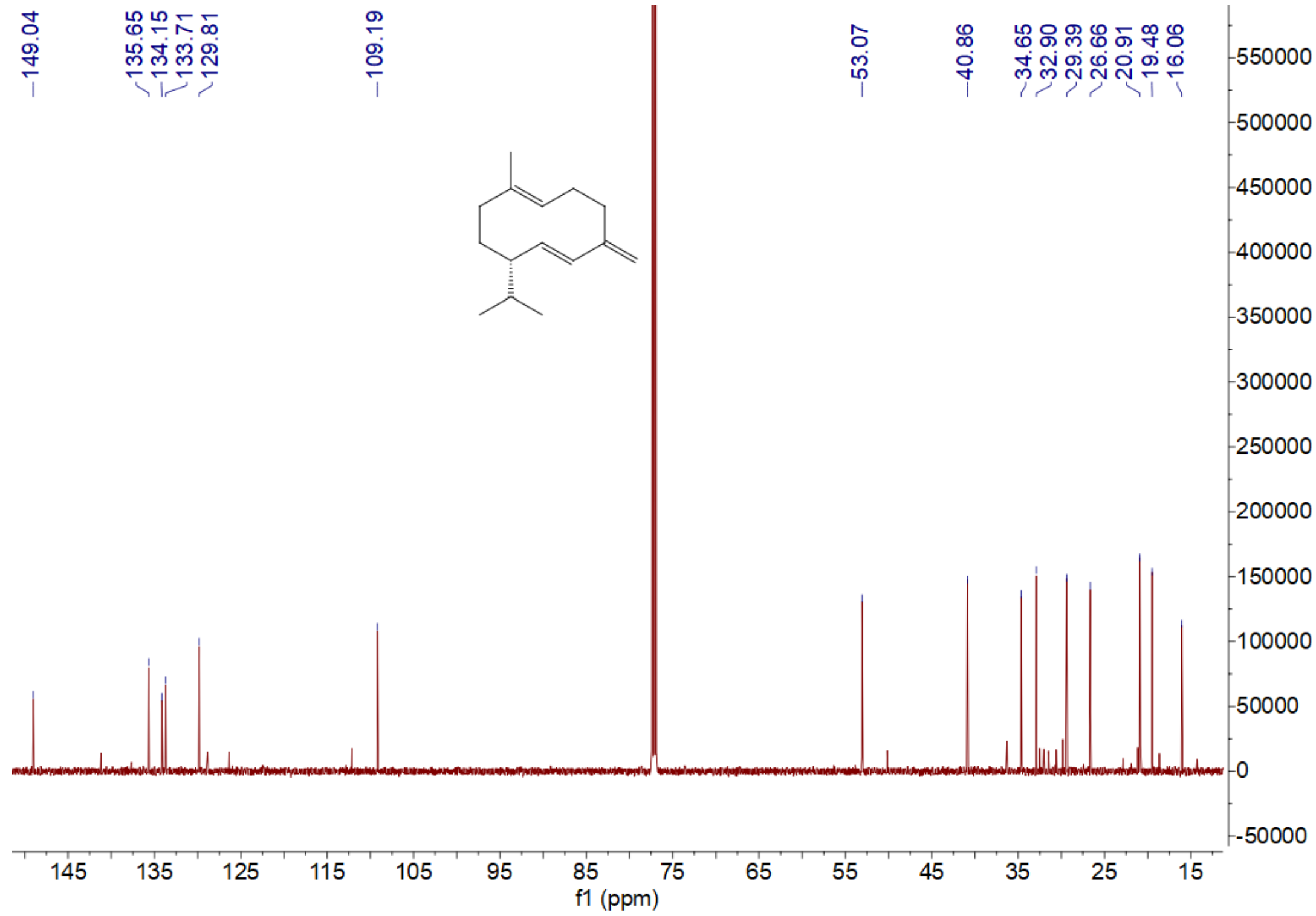

Figure S10. ^{13}C NMR spectrum of (-)-Germacrene D in $CDCl_3$.

Original experimental spectra of the product of AcT1TS3, AcT1TS4 and AcT1TS5: (+)-*β*-cubebene (3).

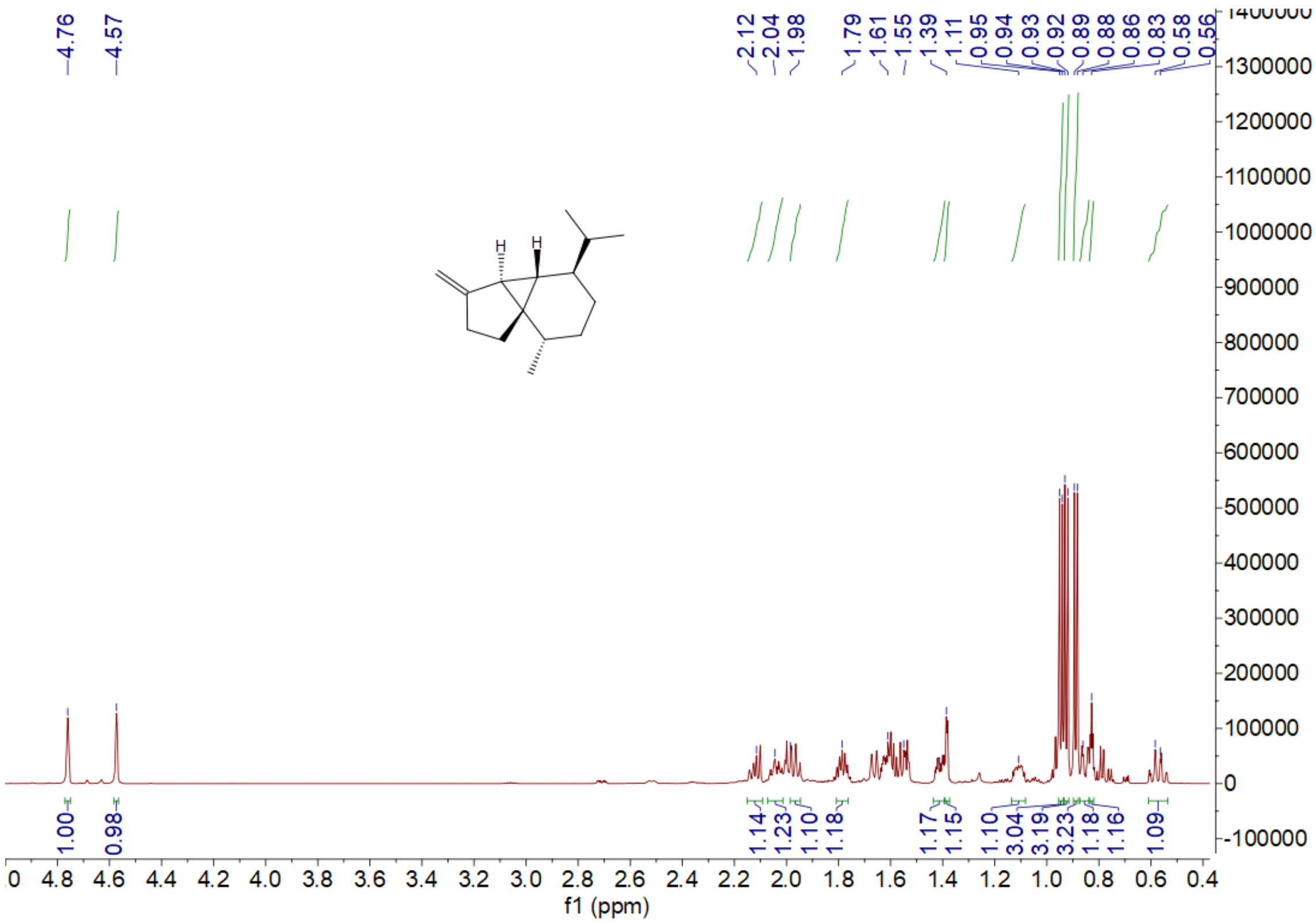

Figure S11. ^{1}H NMR spectrum of (+)-*β*-cubebene in $CDCl_3$.

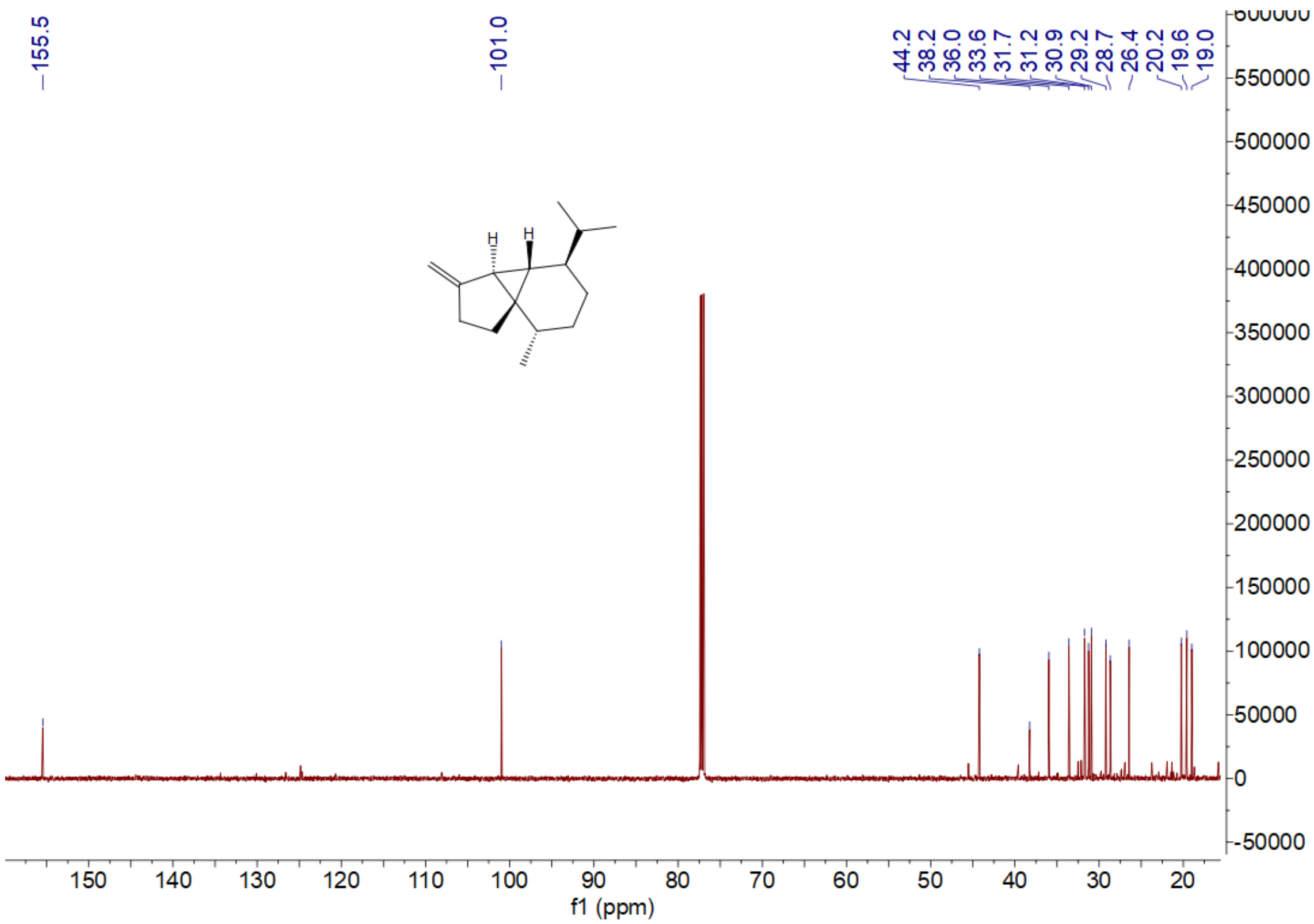

Figure S12. ^{13}C NMR spectrum of (+)-*β*-cubebene in $CDCl_3$.

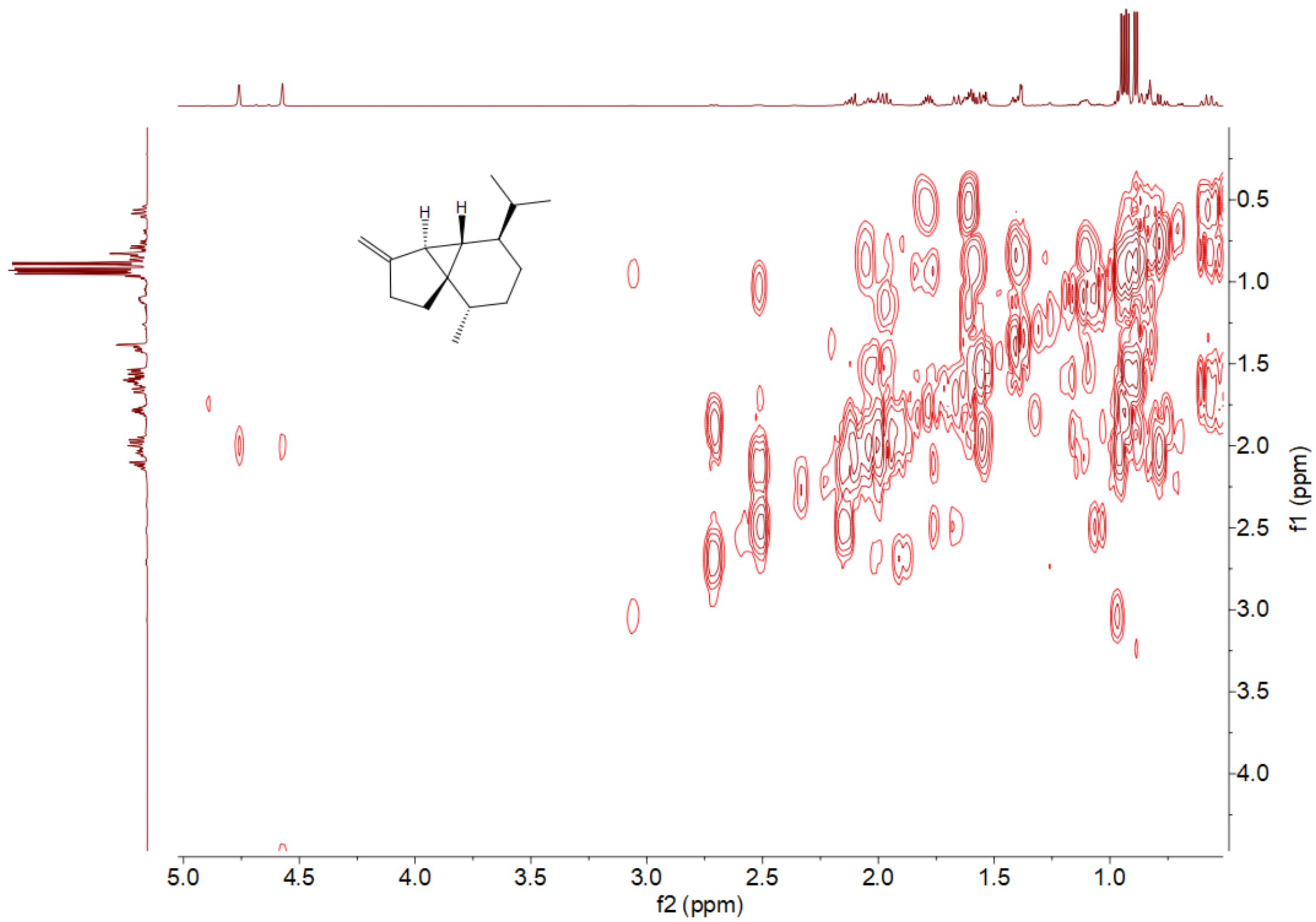

Figure S13. ^{1}H-^{1}H COSY spectrum of (+)-*β*-cubebene in $CDCl_3$.

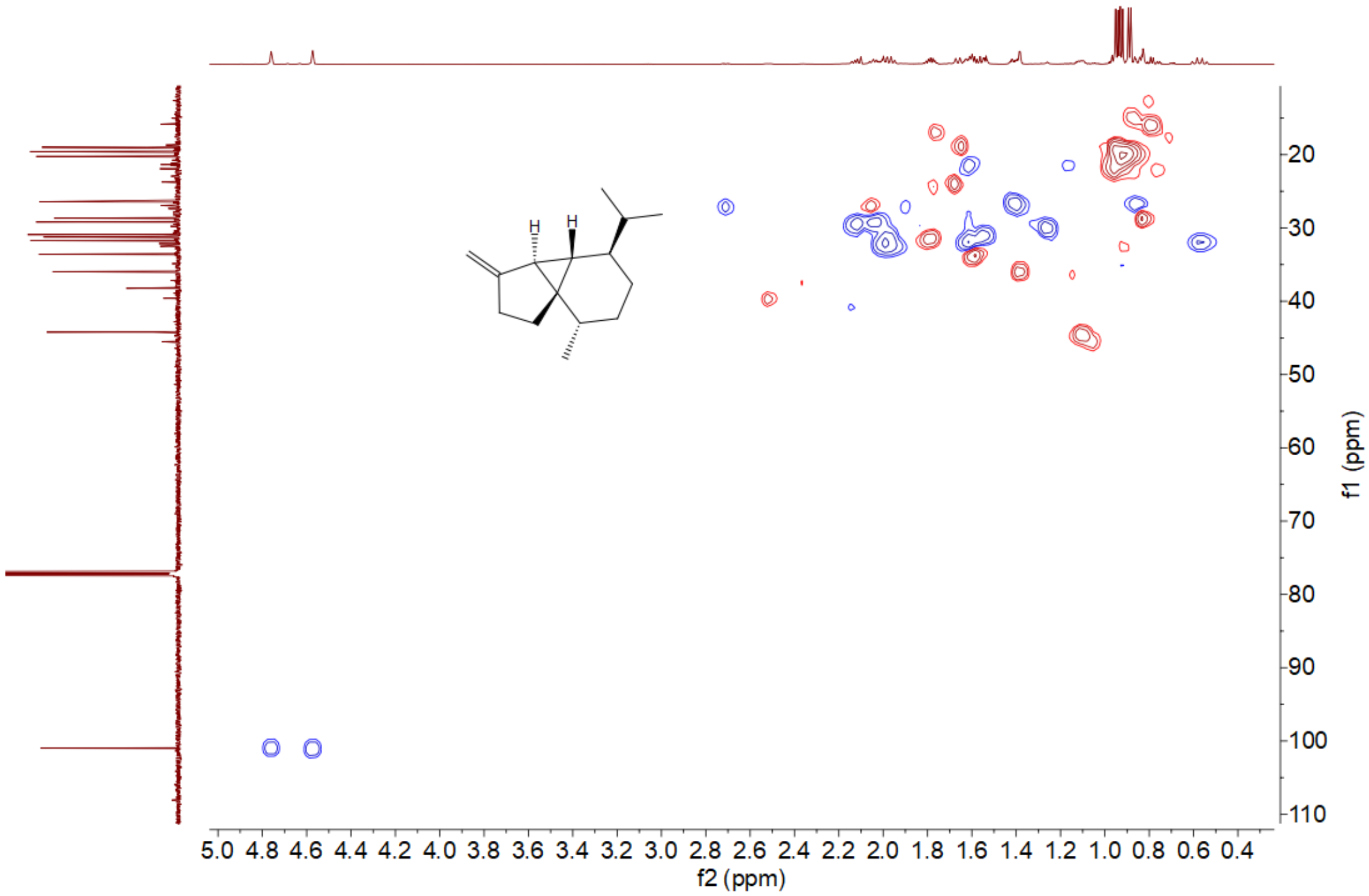

Figure S14. HSQC spectrum of (+)-*β*-cubebene in $CDCl_3$.

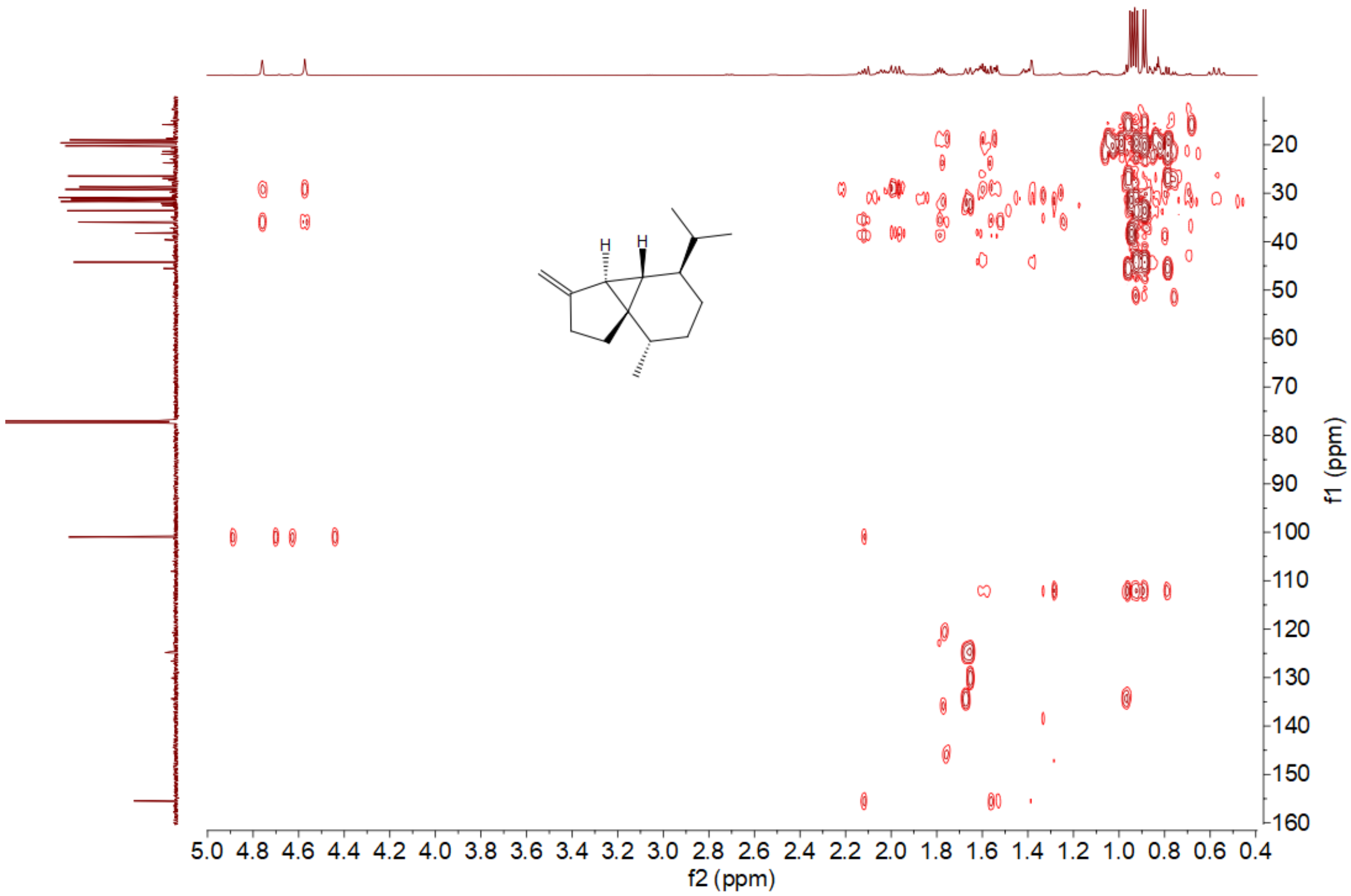

Figure S15. HMBC spectrum of (+)-β-cubebene in $CDCl_3$.

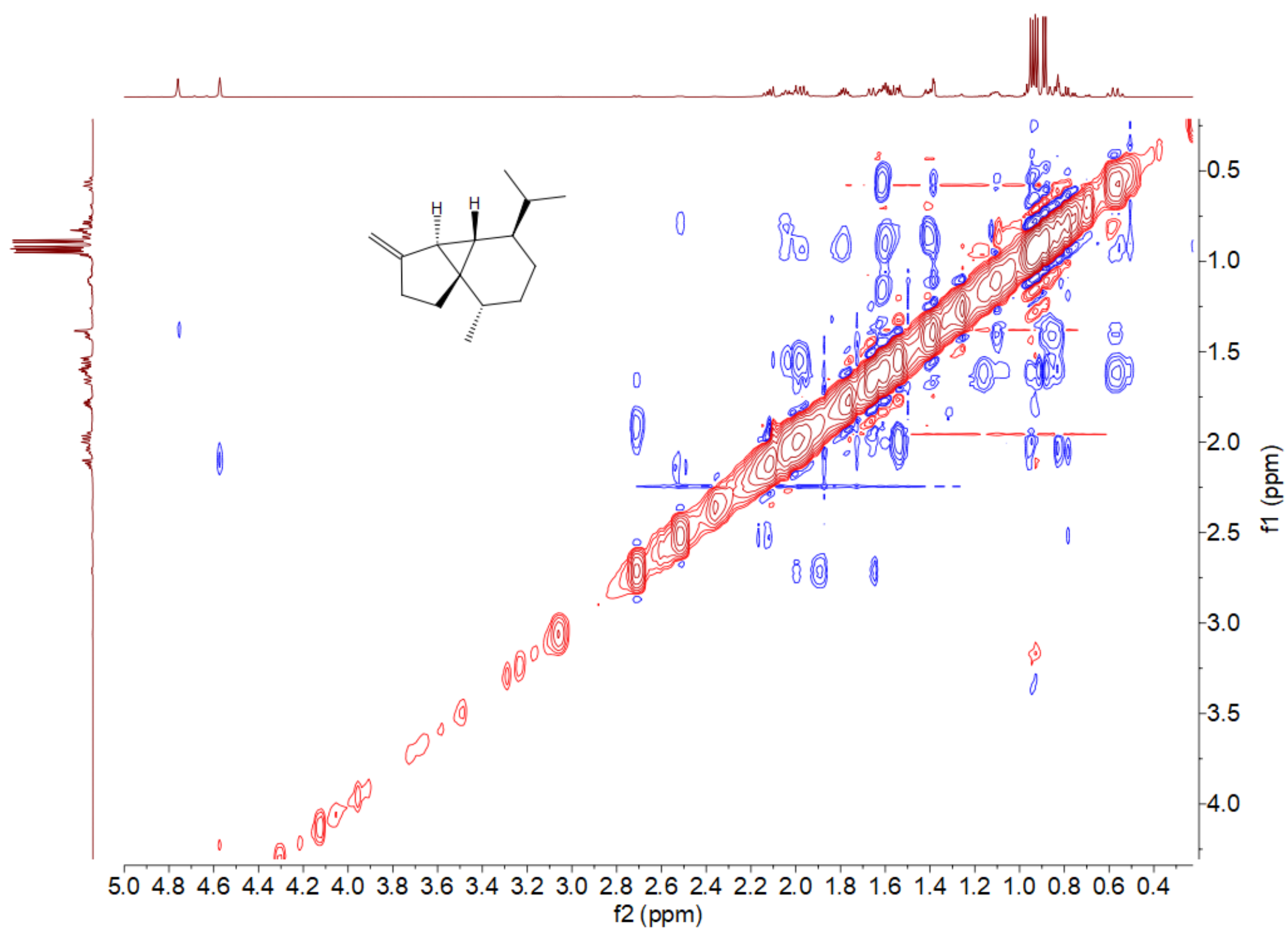

Figure S16. NOESY spectrum of (+)-*β*-cubebene in $CDCl_3$.

Original experimental spectra of the product of AcT1TS8 and AcT1TS9: (-)-Bicyclogermacrene (4).

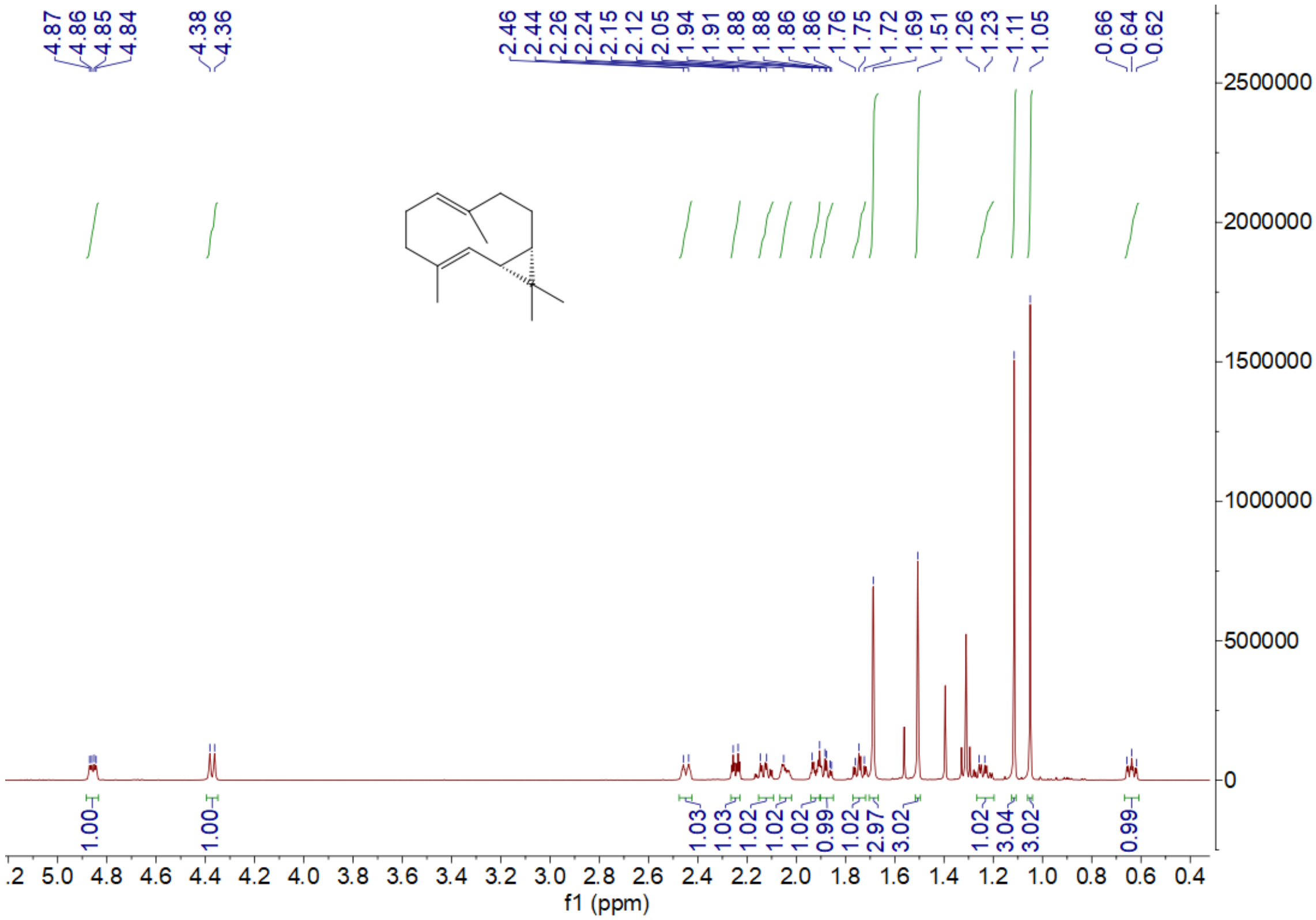

Figure S17. ^{1}H NMR spectrum of (-)-Bicyclogermacrene in $CDCl_3$.

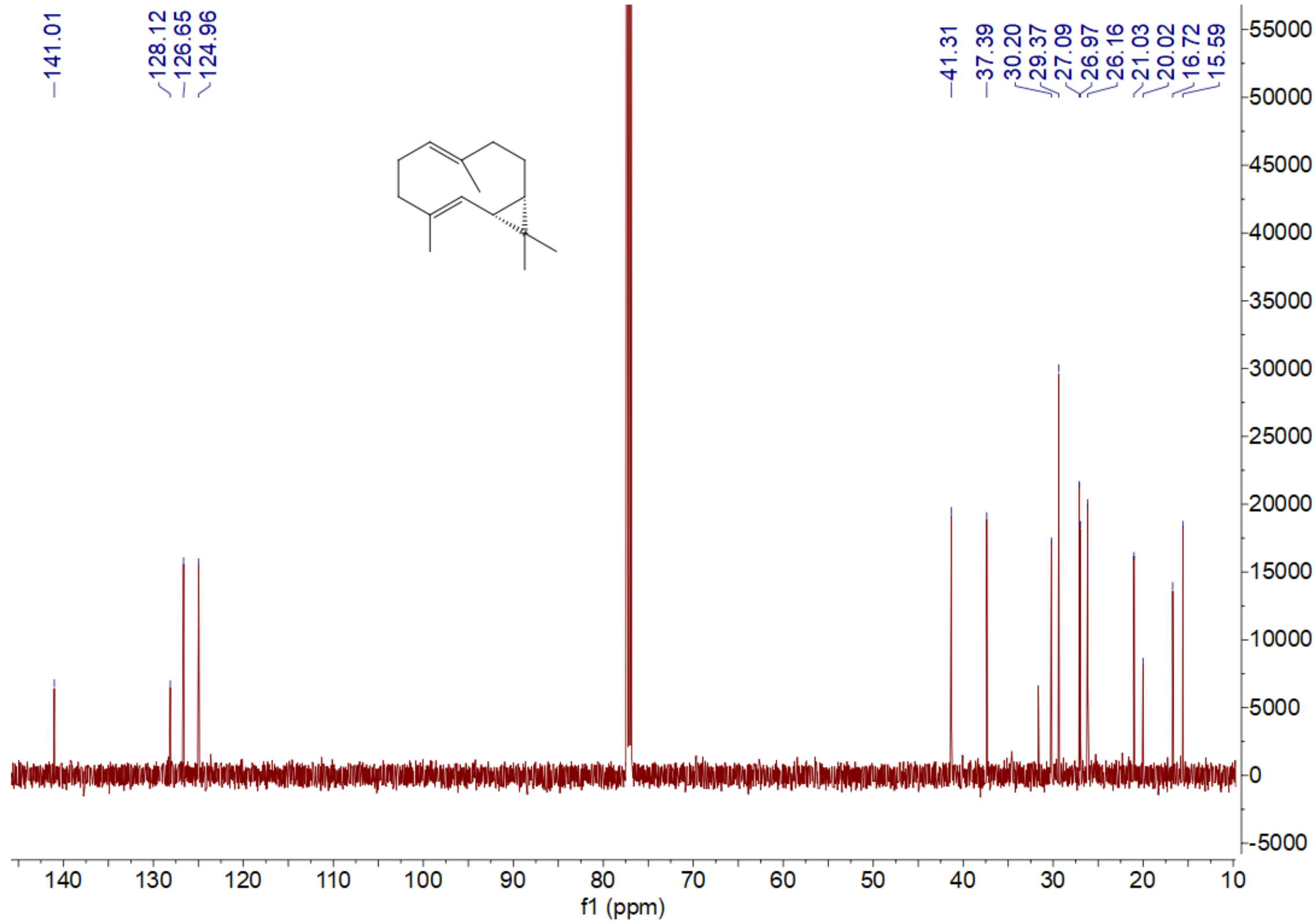

Figure S18. ^{13}C NMR spectrum of (-)-Bicyclogermacrene in $CDCl_3$.

Original experimental spectra of the product of AcT1TS10: (-)-(*E*)-*α*-bisabolene (5).

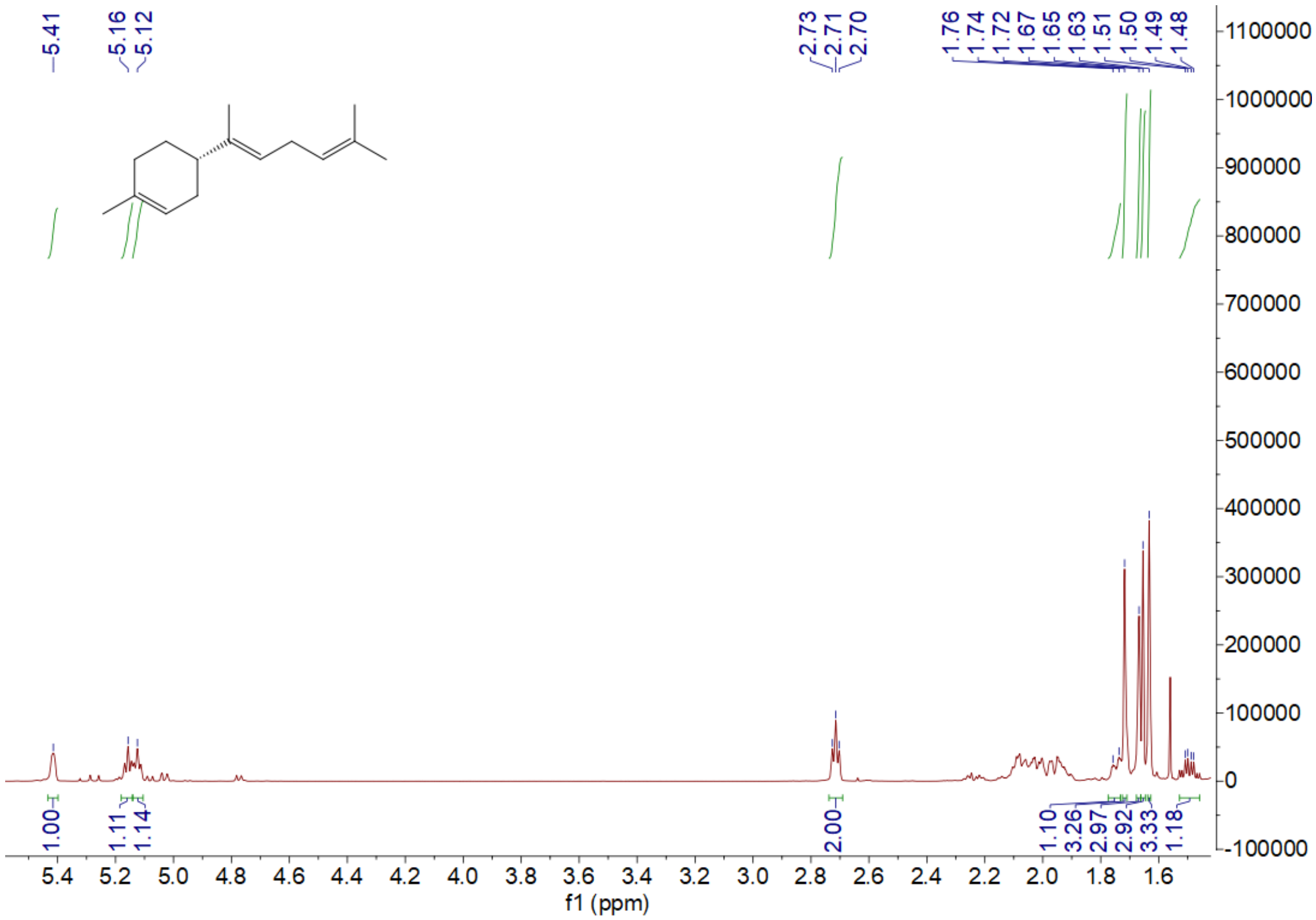

Figure S19. ^{1}H NMR spectrum of (-)-(*E*)-*α*-bisabolene in $CDCl_3$.

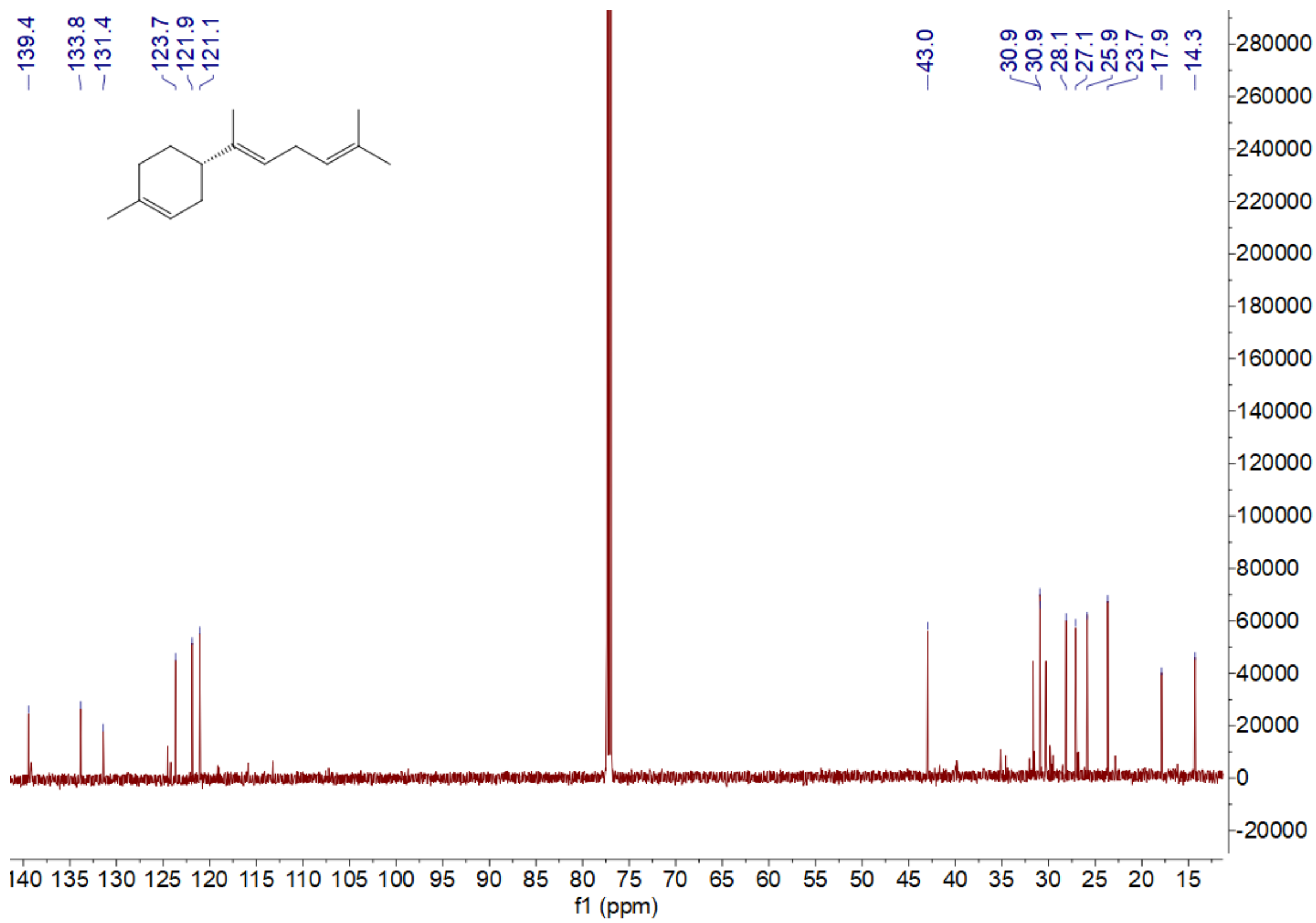

Figure S20. ^{13}C NMR spectrum of (-)-(*E*)-*α*-bisabolene in $CDCl_3$.

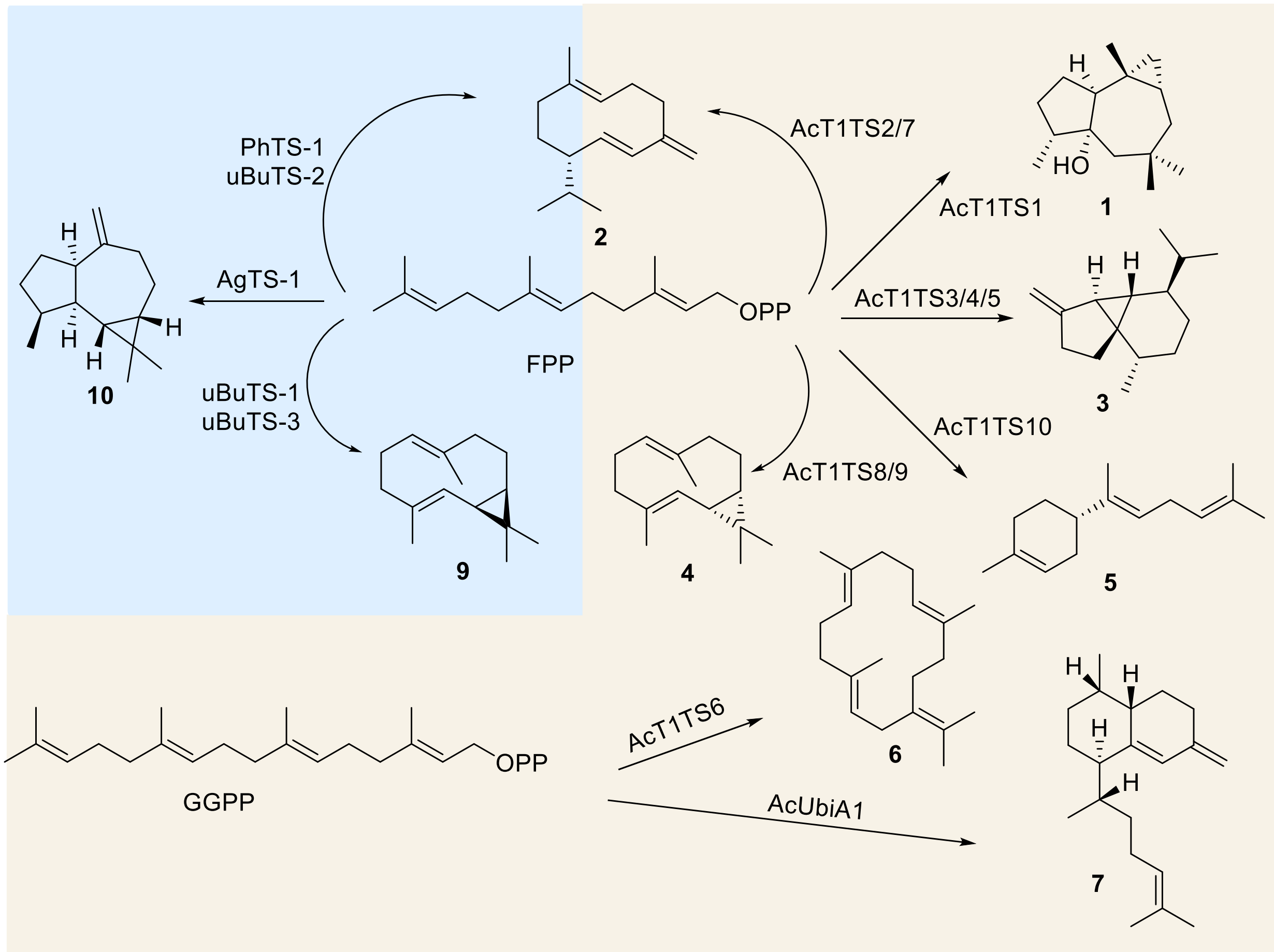

Figure S21. Comparative overview of T1TSs products from sponges. Blue background indicates products previously reported by the Bradley S. Moore group,[13] and yellow background denotes those identified in this study.

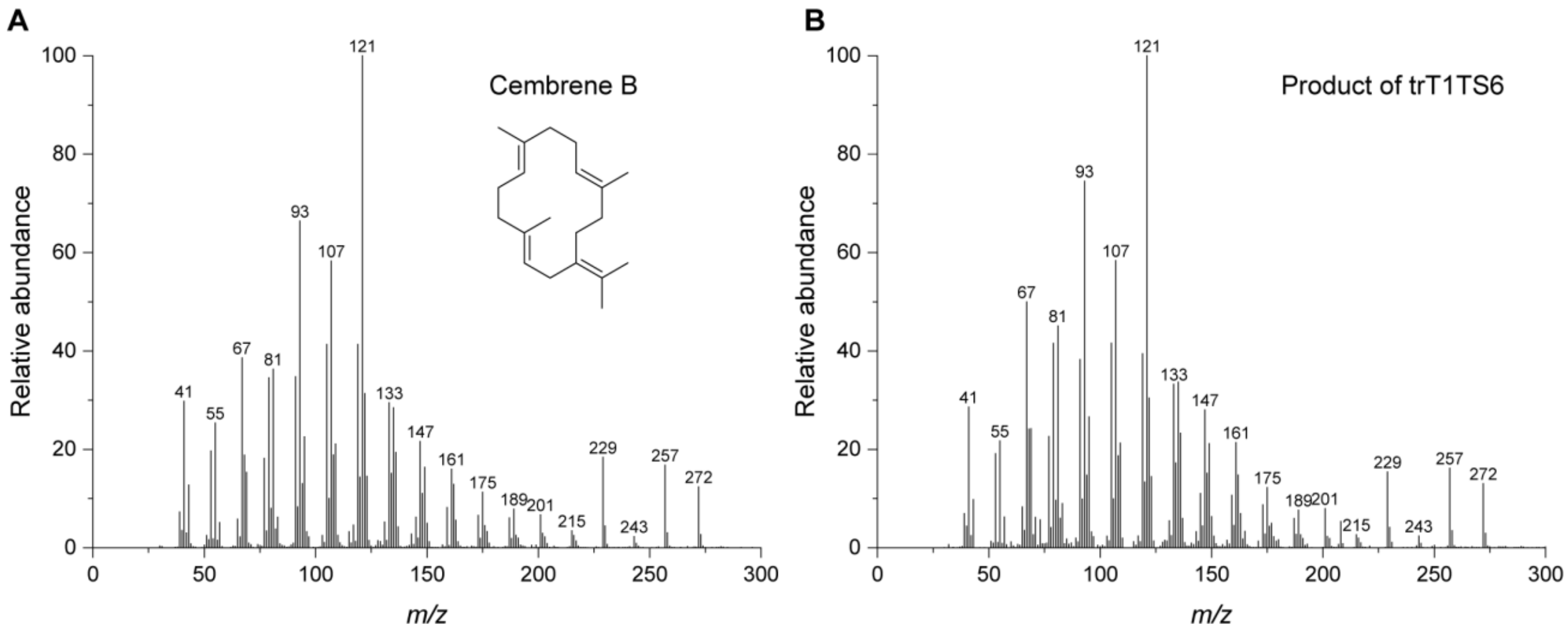

Figure S22. Comparative mass spectrometry analysis of product peaks from trT1TS6 versus cembrene B standard.

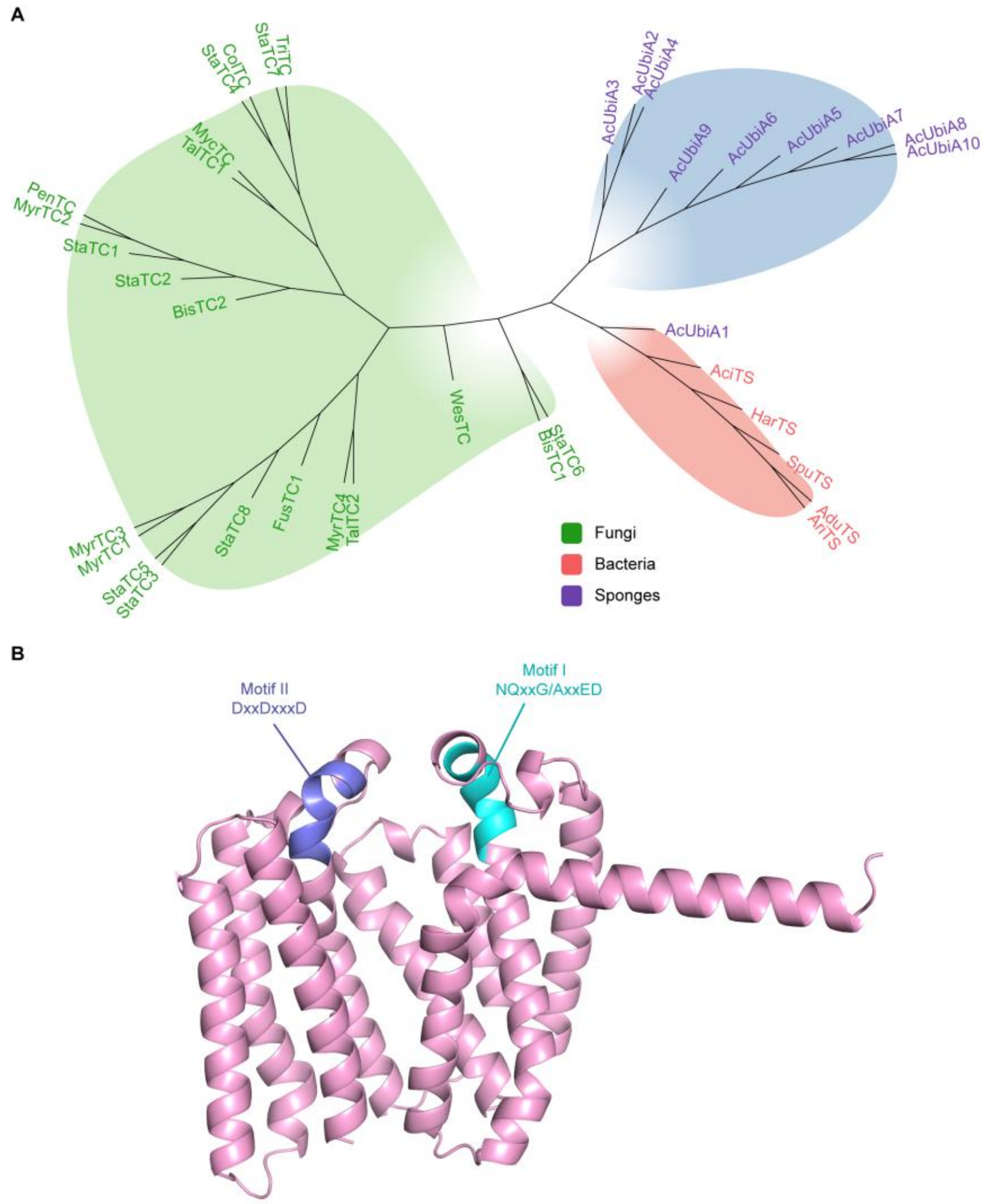

Figure S23. Phylogenetic and structure analysis of UbiA proteins. (**A**) Phylogenetic analysis of UbiA superfamily proteins from bacteria, fungi and A. cavernosa. (**B**) The structure of AcUbiA1 predicted by AlphaFold3. Motif I and motif II are colored by blue and purple, respectively.

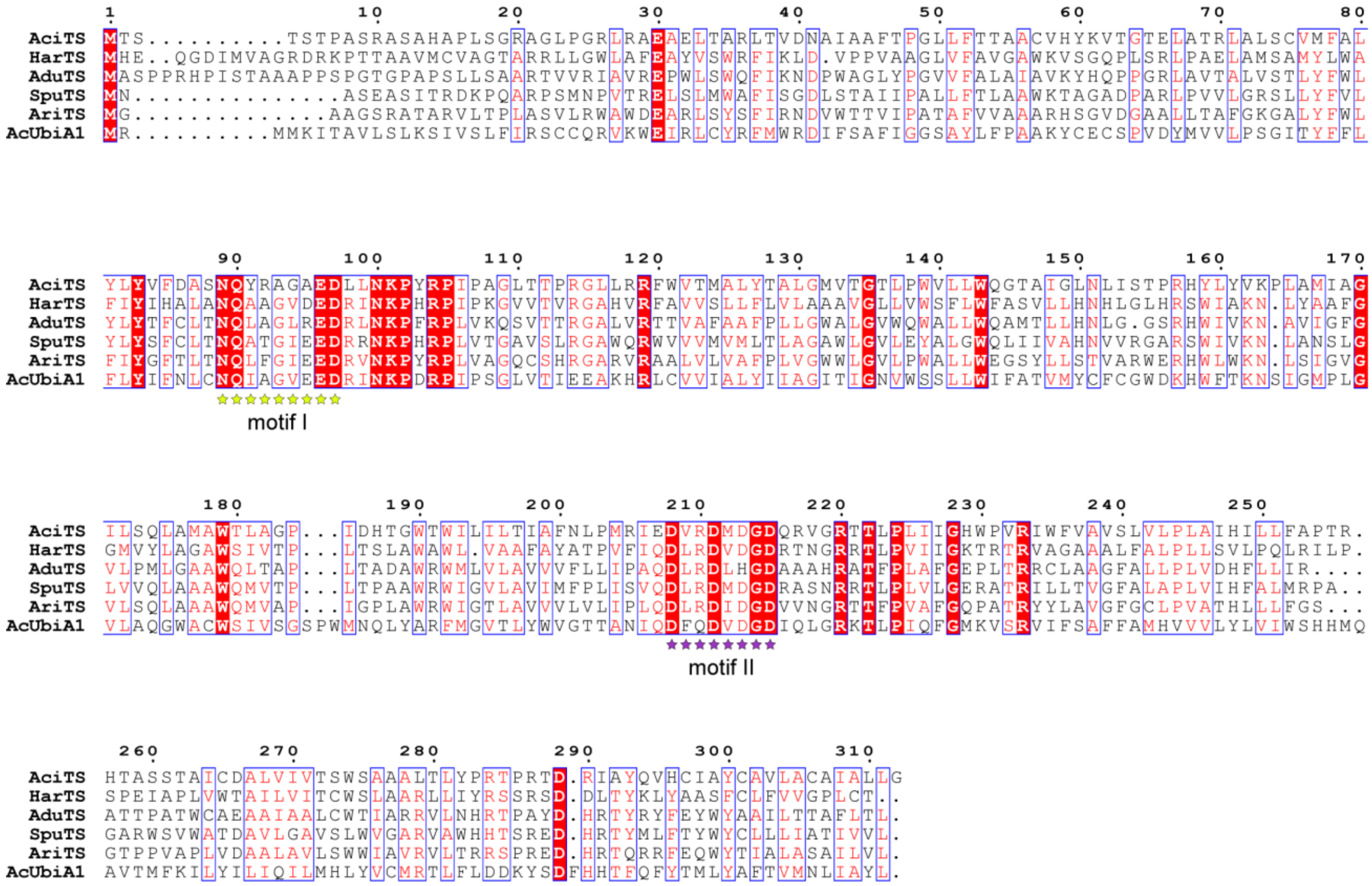

Figure S24. Multiple sequence alignment of AcUbiA1 with bacteria UbiA-type TS. Residues are colored based on conservation (identity: red box with white residue; similarity: red residue; similar across group: blue frame). NQxxG/AxxED (motif I) and DxxDxxxD (motif II) are designated with yelow and purple stars, respectively.

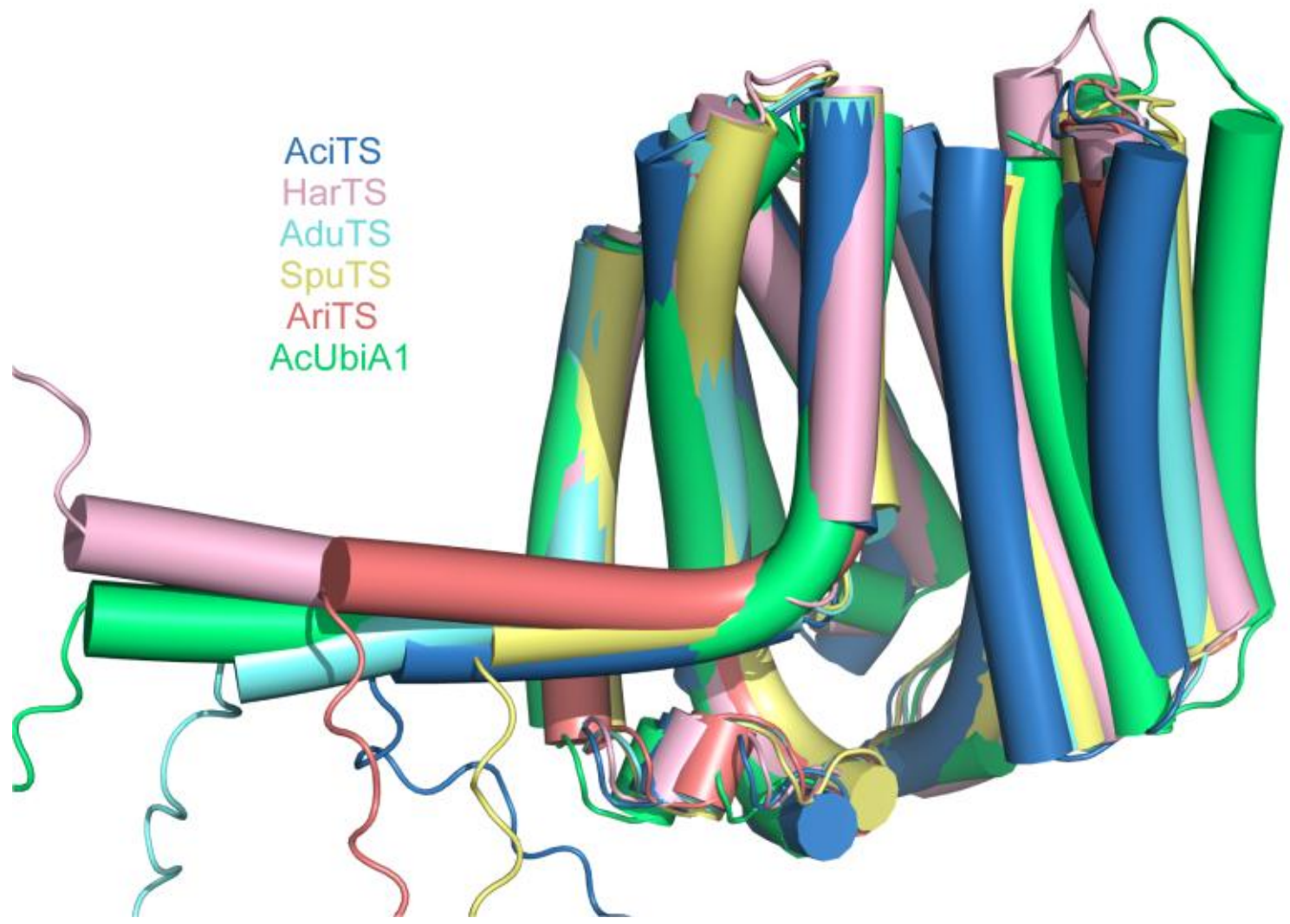

Figure S25. Structural alignment between the AlphaFold3 predicted structures of AcUbiA1 with bacterial UbiA-type TSs.

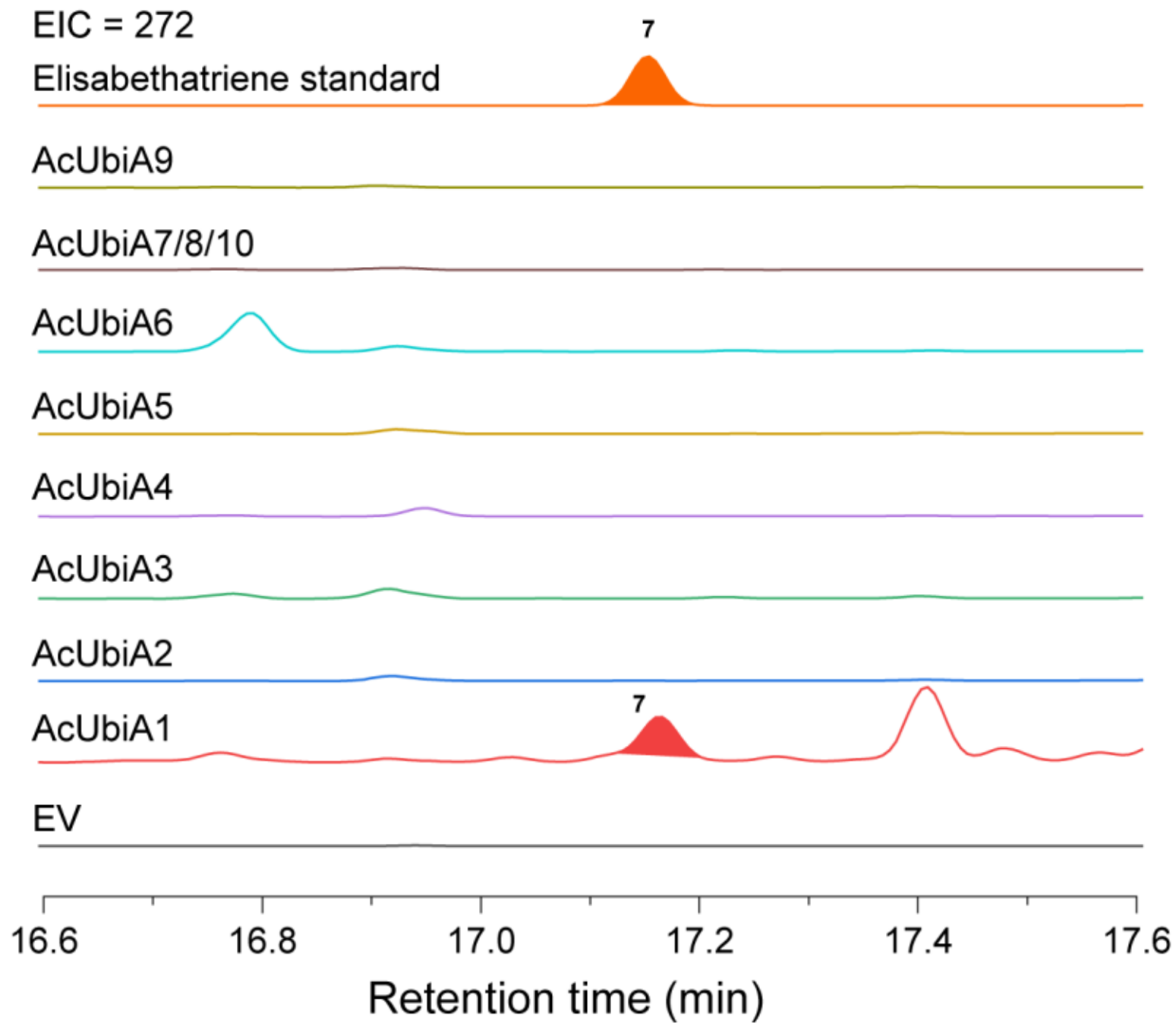

Figure S26. Functional characterizations of AcUbiAs from *A. cavernosa*. Extracted ion chromatograms (m/z = 272) of GC-MS analysis of metabolites produced by *E. coli* chassis engineered to overexpress GGPP along with AcUbiAs or empty vector (EV) control. Standard compound elisabethatriene (**7**) was used as a positive control.

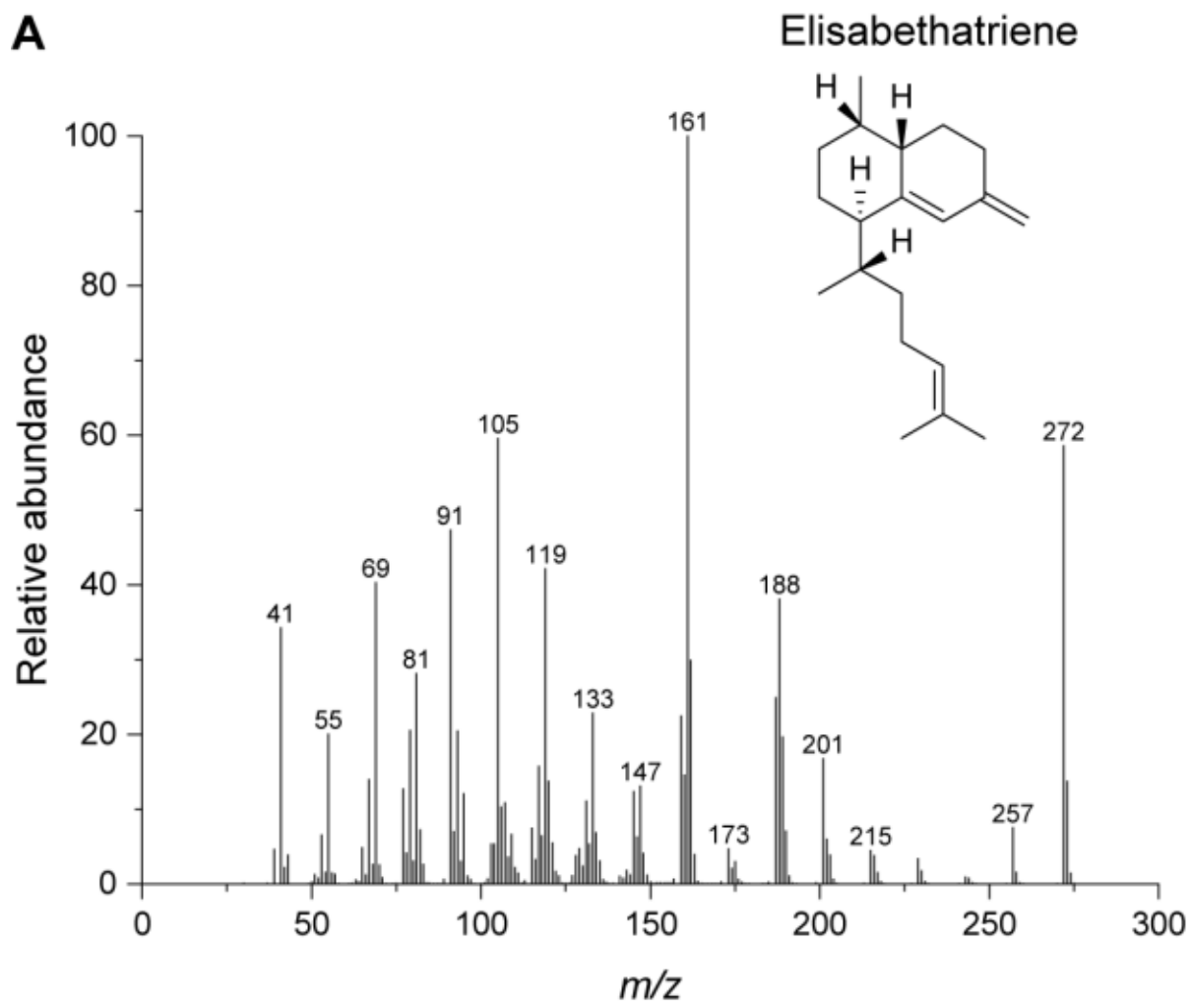

Figure S27. Comparative mass spectrometry analysis of product peaks from AcUbiA1 versus standard compound elisabethatriene.

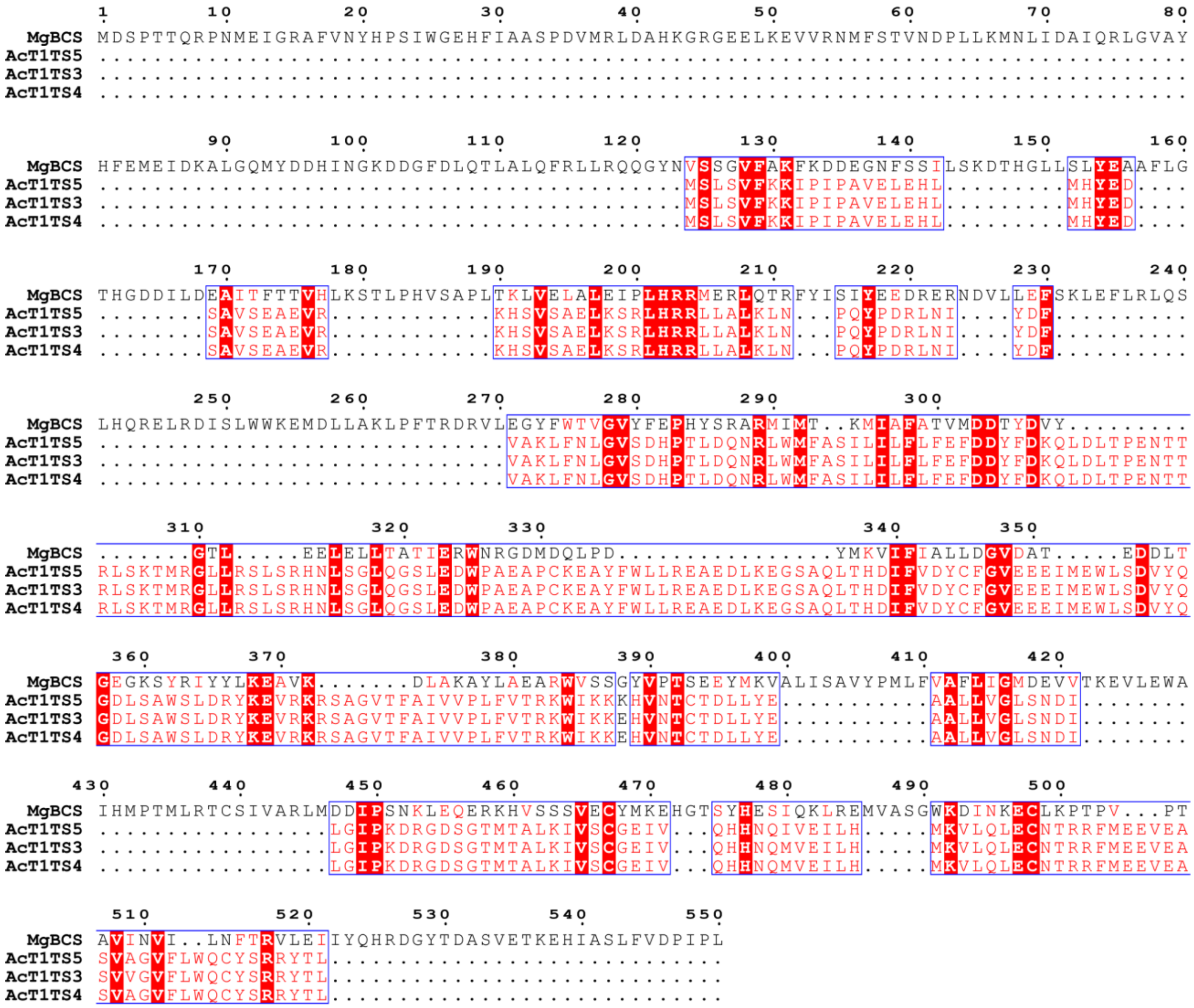

Figure S28. Multiple sequence alignment of AcT1TS3, AcT1TS4, AcT1TS5 with *β*-cubebene synthase from *M. grandiflora* (MgBCS).

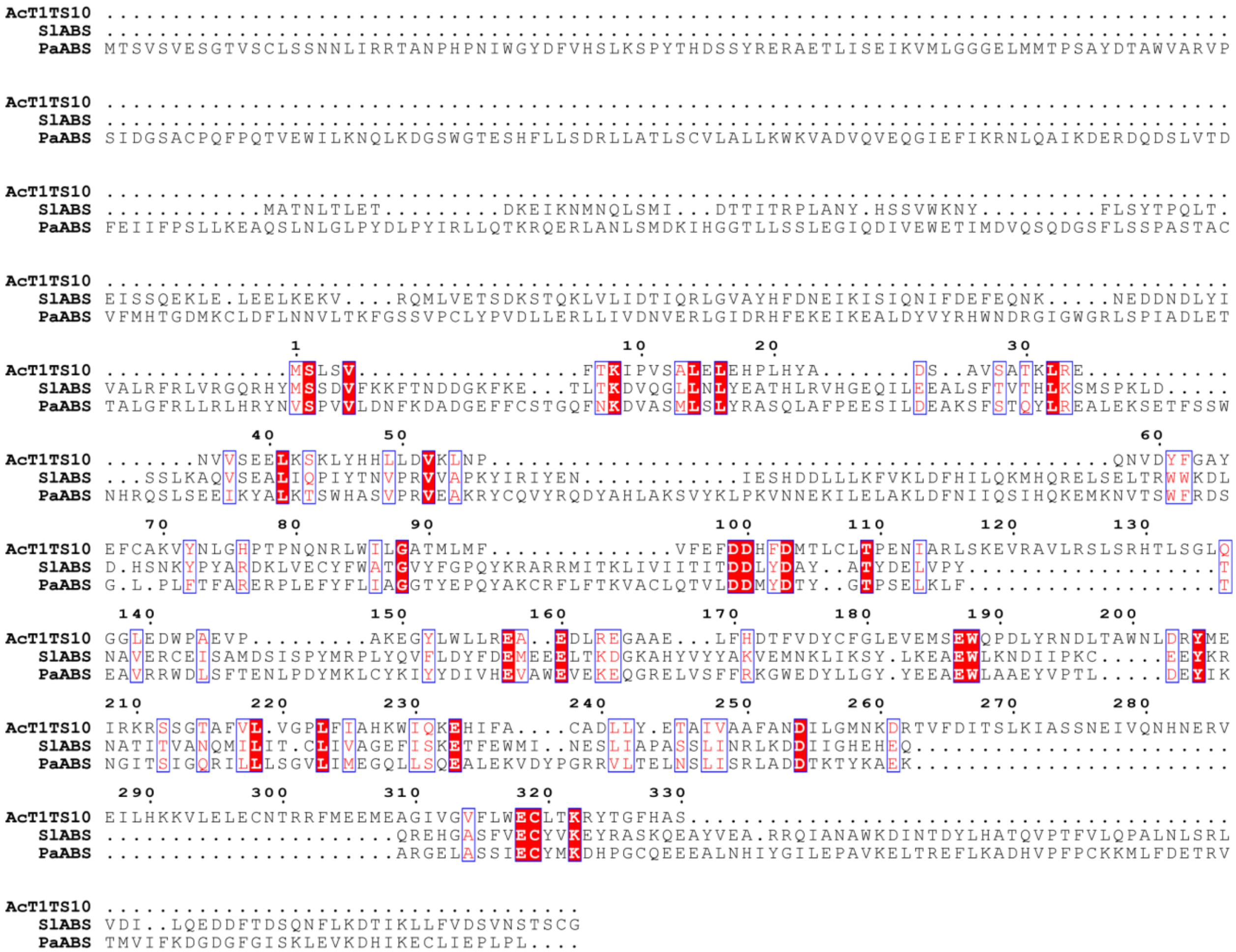

Figure S29. Multiple sequence alignment of AcT1TS10 with *α*-bisabolene synthase from *Picea abies* (PaABS) and *Solanum lycopersicum* (SlABS).

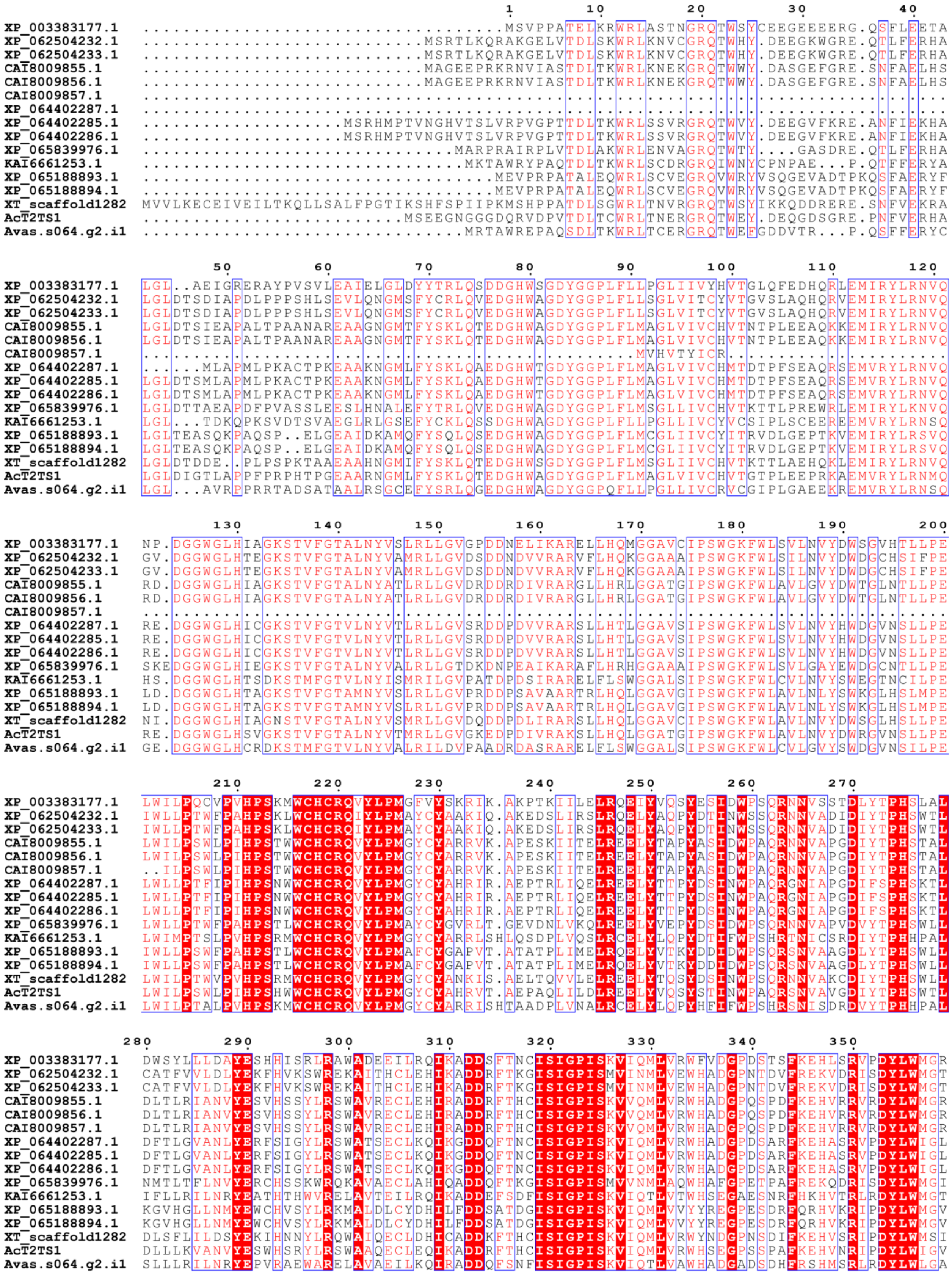

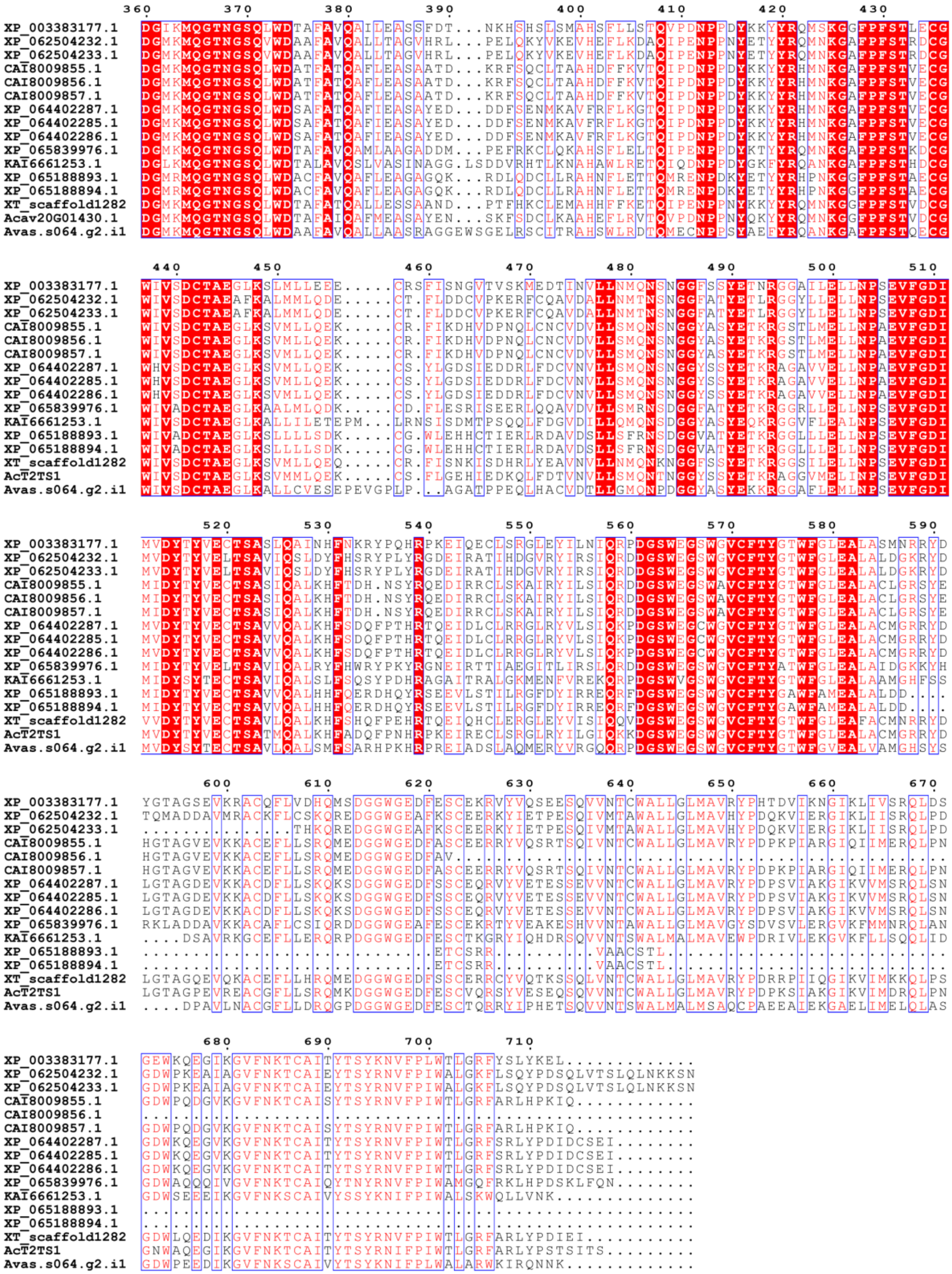

Figure S30. Multiple sequence alignment of AcT2TS1 with other sponges T2TSs.

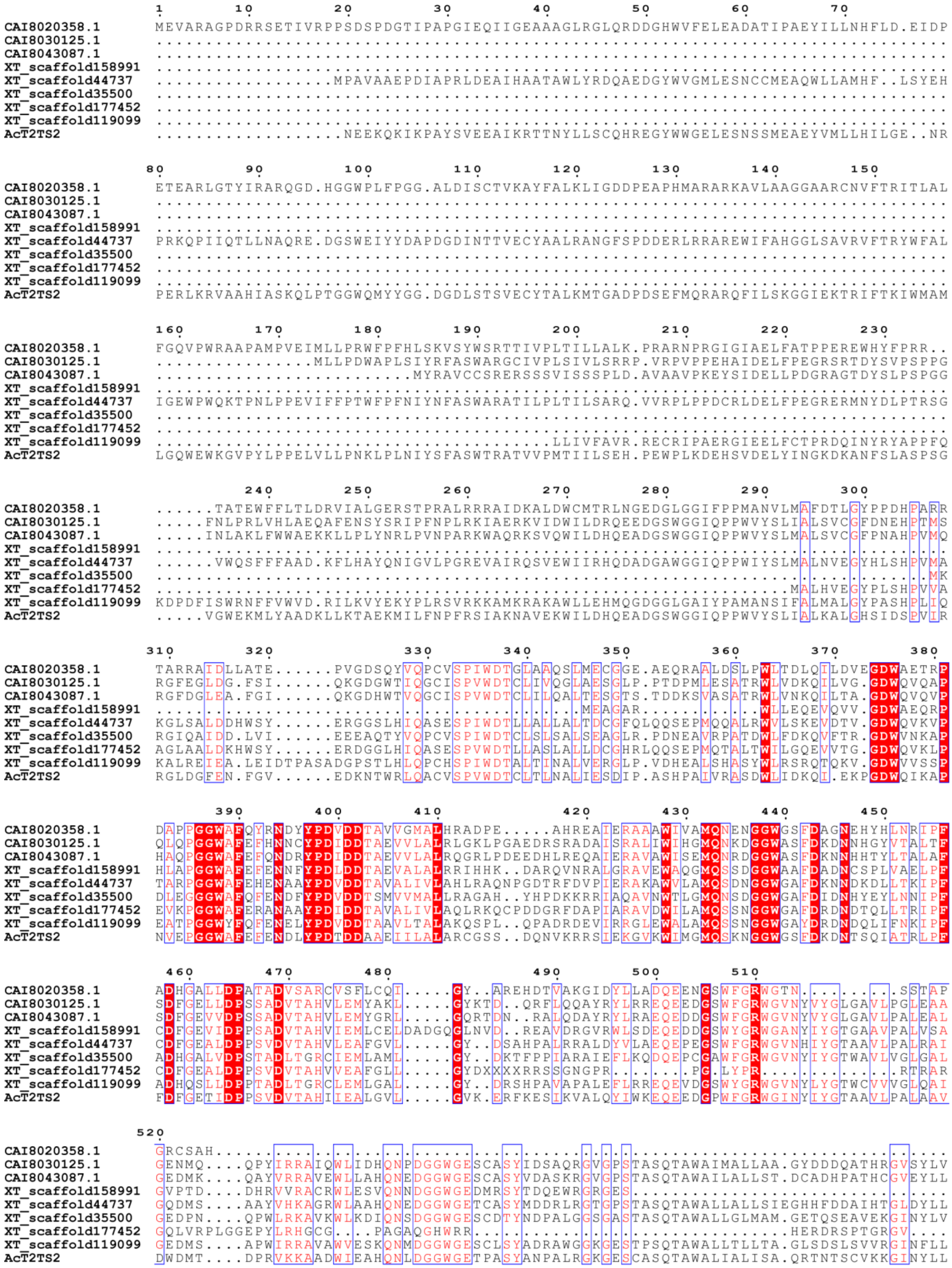
1 10 20 30 40 50 60 70
CAI8020358.1 MEVARAGPDRRSETIVRPPSDSPDGTIPAPGIEQIIGEAAAGLRGLQRDDGHWVFELEADATIPAEYILLNHFLD.EIDP
CAI8030125.1 ....
CAI8043087.1 ....
XT_scaffold158991 ....
XT_scaffold44737 ..................MPAVAAEPDIAPRLDEAIHAATAWLYRDQAEDGYWVGMLESNCCMEAQWLLAMHF..LSYEH
XT_scaffold35500 ....
XT_scaffold177452 ....
XT_scaffold119099 ....
AcT2TS2 ...................NEEKQKIKPAYSVEEAIKRTTNYLLSCQHREGYWWGELESNSSMEAEYVMLLHILGE..NR
80 90 100 110 120 130 140 150
CAI8020358.1 ETEARLGTYIARQGD.HGGWPLFPGG.ALDISCTVKAYFALKLIGDDPEAPHMARARKAVLAAGGAARCNVFTRITLAL
CAI8030125.1 ....
CAI8043087.1 ....
XT_scaffold158991 ....
XT_scaffold44737 PRKQPIIQTLLNAQRE.DGSWEIYYDAPDGDINTTVECYAALRANGFSPDDERLRRAREWIFAHGGLSAVRVFTRYWFAL
XT_scaffold35500 ....
XT_scaffold177452 ....
XT_scaffold119099 ....
AcT2TS2 PERLKRVAAHIASKQLPTGGWQMYYGG.DGDLSTSVECYTALKMTGADPDSEFMQRARQFILSKGGIEKTRIFTKIWMAM
160 170 180 190 200 210 220 230
CAI8020358.1 FGQVPWRAAPAMPVEIMLLPRWFPFHLSKVSYWSRTTIVPLTILLALK.PRARNPRGIGIAELFATPPEREWHYFPRR..
CAI8030125.1 ................MLLPDWAPLSIYRFASWARGCIVPLSIVLSRRP.VRPVPPEHAIDELFPEGRSRTDYSVPSPPG
CAI8043087.1 ..........................MYRAVCCSRERSSSVISSSPLD.AVAAVPKEYSIDELLPDGRAGTDYSLPSPGG
XT_scaffold158991 ....
XT_scaffold44737 IGEWPWQKTPNLPPEVIFFPTWFPFNIYNFASWARATILPLTILSARQ.VVRPLPPDCRLDELFPEGRERMNYDLPTRSG
XT_scaffold35500 ....
XT_scaffold177452 ....
XT_scaffold119099 ....LLIVFAVR.RECRIPAERGIEELFCTPRDQINYRYAPPFQ
AcT2TS2 LGQWEWKGVPYLPPELVLLPNKLPLNIYSFASWTRATVVPMTIILSEH.PEWPLKDEHSVDELYINGKDKANFSLASPSG
240 250 260 270 280 290 300
CAI8020358.1 ......TATEWFFLTLDRVIALGERSTPRALRRRAIDKALDWCMTRLNGEDGLGGIFPPMANVLMAFDTLGYPPDHPARR
CAI8030125.1 .....FNLPRLVHLAEQAFENSYSRIPFNPLRKIAERKVIDWILDRQEEDGSWGGIQPPWVYSLIALSVCGFDNEHPTMS
CAI8043087.1 .....INLAKLFWWAEKKLLPLYNRLPVNPARKWAQRKSVQWILDHQEADGSWGGIQPPWVYSLMALSVCGFPNAHPVMQ
XT_scaffold158991 ....
XT_scaffold44737 .....VWQSFFFAAD.KFLHAYQNIGVLPGREVAIRQSVEWIIRHQDADGAWGGIQPPWIYSLMALNVEGYHLSHPVMA
XT_scaffold35500 ....MK
XT_scaffold177452 ....MALHVEGYPLSHPVVA
XT_scaffold119099 KDPDFISWRNFFVWVD.RILKVYEKYPLRSVRKKAMKRAKAWLLEHMQGDGGLGAIYPAMANSIFALMALGYPASHPLIQ
AcT2TS2 .....VGWEKMLYAADKLLKTAEKMILFNPFRSIAKNAVEKWILDHQEADGSWGGIQPPWVYSLIALKALGHSIDSPVIR
310 320 330 340 350 360 370 380
CAI8020358.1 TARRAIDLLATE......PVGDSQYVQPCVSPIWDTGLAAQSLMECGGE.AEQRAALDSLPWLTDLQILDVEGDWAETRP
CAI8030125.1 RGFEGLDG.FSI......QKGDGWTIQGCISPVWDTCLIVQGLAESGLP.PTDPMLESATRWLVDKQILVG.GDWQVQAP
CAI8043087.1 RGFDGLEA.FGI......QKGDHWTVQGCISPVWDTCLILQALTESGTS.TDDKSVASATRWLVNKQILTA.GDWQVQVP
XT_scaffold158991 ....MEAGAR............WLLEQEVQVV.GDWAEQRP
XT_scaffold44737 KGLSALDDHWSY......ERGGSLHIQASESPIWDTLLALLALTDCGFQLQQSEPMQQALRWVLSKEVDTV.GDWQVKVP
XT_scaffold35500 RGIQAIDD.LVI......EEEAQTYVQPCVSPIWDTCLSLSALSEAGLR.PDNEAVRPATDWLFDKQVFTR.GDWVNKAP
XT_scaffold177452 AGLAALDKHWSY......ERDGGLHIQASESPVWDTLLASLALLDCGHRLQQSEPMQTALTWILGQEVVTG.GDWQVKLP
XT_scaffold119099 KALREIEA.LEIDTPASADGPSTLHLQPCHSPIWDTALTINALVERGLP.VDHEALSHASYWLRSRQTQKV.GDWVVSSP
AcT2TS2 RGLDGFEN.FGV......EDKNTWRLQACVSPVWDTCLTLNALIESDIP.ASHPAIVRASDWLIDKQI.EKPGDWQIKAP
390 400 410 420 430 440 450
CAI8020358.1 DAPGGWAFQYRNDYYPDVDDTAVVGMALHRADPE.....AHREAIERAAAWIVAMQNENGGWGSFDAGNEHYHLNRIPF
CAI8030125.1 QLQPGGWAFEFHNNCYPDIDDTAEVVLALRLGKLPGAEDRSRADAISRALIWIHGMQNKDGGWASFDKDNNHGYVTALTF
CAI8043087.1 HAQPGGWAFEFQNDRYPDIDDTAEVVLALRQGRLPDEEDHLREQAIERAVAWISEMQNRDGGWASFDKNNHHTYLTALAF
XT_scaffold158991 HLAPGGWAFEFENNFYPDLDDTAVALALRRIHHK..DARQVNRALGRAVEWAQGMQSSDGGWAAFDADNCSPLVAELPF
XT_scaffold44737 TARPGGWAFEHENAAYPDVDDTAVALIVLAHLRAQNPGDTRFDVPIERAKAWVLAMQSDNGGWGAFDKNNDKDLLTKIPF
XT_scaffold35500 DLEGGGWAFQFENDFYPDVDDTSMVVMALLRAGAH..YHPDKKRRIAQAVNWTLGMQNSDGGWGAFDIDNHYEYLNNIPF
XT_scaffold177452 EVKPGGWAFERANAAYPDIDDTAVALIVLAQLRKQCPDDGRFDAPIARAVDWILAMQSSNGGWGAFDRDNDTQLLTRIPF
XT_scaffold119099 EATPGGWYFQFENELYPDVDDTAAVLTALAKQSPL..QPADRDEVIRRGLEWALAMQSSNGGWGAYDRDNDQLIFNKIPF
AcT2TS2 NVEPGGWAFEFENDLYPDTDDAAEIILALARCGSS..DQNVKKRRSIEKGVKWIMGMQSKNGGWGSFDKDNTSQIATRLPF
460 470 480 490 500 510
CAI8020358.1 ADHGALLDPATADVSARCVSFLCQI.....GY..AREHDTVAKGIDYLLADQEENGSWFGRWGTN..........SSTAP
CAI8030125.1 SDFGELLDPSSADVTAHVLEMYAKL.....GYKTD..QRFLQQAYRYLRREQEEDGSWFGRWGVNYVYGLGAVLPGLEAA
CAI8043087.1 SDFGEVVDPSSADVTAHVLEMYGRL.....GQRTDN..RALQDAYRYLRAEQEDDGSWFGRWGVNYVYGLGAVLPALEAL
XT_scaffold158991 CDFGEVIDPPSADVTAHVIEMLCELDADGQGLNVD..REAVDRGVRWLSDEQEDDGSWYGRWGANYIYGTGAAVPALVSA
XT_scaffold44737 CDFGEALDPPSVDVTAHVLEAFGVL.....GY..DSAHPALRRALDYVLAEQEPEGSWFGRWGVNHIYGTAAVLPALRAI
XT_scaffold35500 ADHGALVDPSTADLTGRCIEMLAML.....GY..DKTFPPIARAIEFLKQDQEPCGAWFGRWGVNYIYGTWAVLVGLGAL
XT_scaffold177452 CDFGEALDPPSVDVTAHVVEAFGLL.....GYDXXXXRRSSGNGPR........PG.LYPR..............RTRAR
XT_scaffold119099 ADHQSLLDPPTADLTGRCLEMLGAL.....GY..DRSHPAVAPALEFLRREQEVDGSWYGRWGVNYLYGTWCVVVGLQAI
AcT2TS2 FDFGETIDPPSVDVTAHIIEALGVL.....GVK.ERFKESIKVALQYIWKEQEEDGPWFGRWGINYIYGTAAVLPALAAV
520
CAI8020358.1 GRCSAH..
CAI8030125.1 GENMQ....QPYIRRAIQWLIDHQNPDGGWGESCASYIDSAQRGVGPSTASQTAWAIMALLAA.GYDDDQATHRGVSYLV
CAI8043087.1 GEDMK....QAYVRRAVEWLLAHQNEDGGWGESCASYVDASKRGVGPSTASQTAWAILALLST.DCADHPATHCGVEYLL
XT_scaffold158991 GVPTD....DHRVVRACRWLESVQNNDGGWGEDMRSYTDQEWRGRGES................................
XT_scaffold44737 GQDMS....AAYVHKAGRWLAAHQNEDGGWGETCASYMDDRLRGTGPSTASQTAWALLALLSIEGHHFDDAIHTGLDYLL
XT_scaffold35500 GEDPN....QPWLRKAVKWLKDIQNSDGGWGESCDTYNDPALGGSGASTASQTAWALLGLMAM.GETQSEAVEKGINYLV
XT_scaffold177452 GQLVRPLGGEPYLRHGCG...PAGAQGHWRR.................................HERDRSPTGRGV....
XT_scaffold119099 GEDMS....APWIRRAVAWVESKQNMDGGWGESCLSYADRAWGGKGESTPSQTAWALLTLLTA.GLSDSLSVVRGINFLL
AcT2TS2 DWDMT....DPRVKKAADWIEAHQNLDGGWGETPASYANPALRGKGESCASQTAWALIALISA.QRTNTSCVKKGINYLL

```
CAI8020358.1       ................................................................................
CAI8030125.1       DTQLAEGNWDEPYFTGTGFPGYLKGERVDRVPQPGERGFQGEELSAGFMINYHLYRNTWPLIALARYRQRTAQRVSRAKS
CAI8043087.1       RTQQPDGQWDQPYFTGTGFP.........................AGFMINYHLYRNTWPLLALGRYRNQMRQADGNAIQ
XT_scaffold158991  ................................................................................
XT_scaffold44737   ................................................................................
XT_scaffold35500   RNFDGAKGWEEAAFTGTGFP.........................RVFYLRYHGYSHYFPVWALGVYARYRQGLETRQDA
XT_scaffold177452  .....AGRPPEPGRR...LG.........................RDLRVVYG...........................
XT_scaffold119099  RRQLDNGTWCEPYHVGTGFP.........................RVFYLRYHGYSKYFPMWALAMYRNVRMHGQTRAQA
AcT2TS2            ATQTEDGTWNEPQYTATG..............................................................

CAI8020358.1       ............................
CAI8030125.1       QSNGHQAVQAEGRCSTILG.........
CAI8043087.1       IATSI.......................
XT_scaffold158991  ............................
XT_scaffold44737   ............................
XT_scaffold35500   LVREGPVDLGVLPVLDSKYYRSRFPTPA
XT_scaffold177452  ............................
XT_scaffold119099  LQDIAQSARQRQP...............
AcT2TS2            ............................
```

Figure S31. Multiple sequence alignment of AcT2TS2 with other sponge bacteria-type T2TSs.

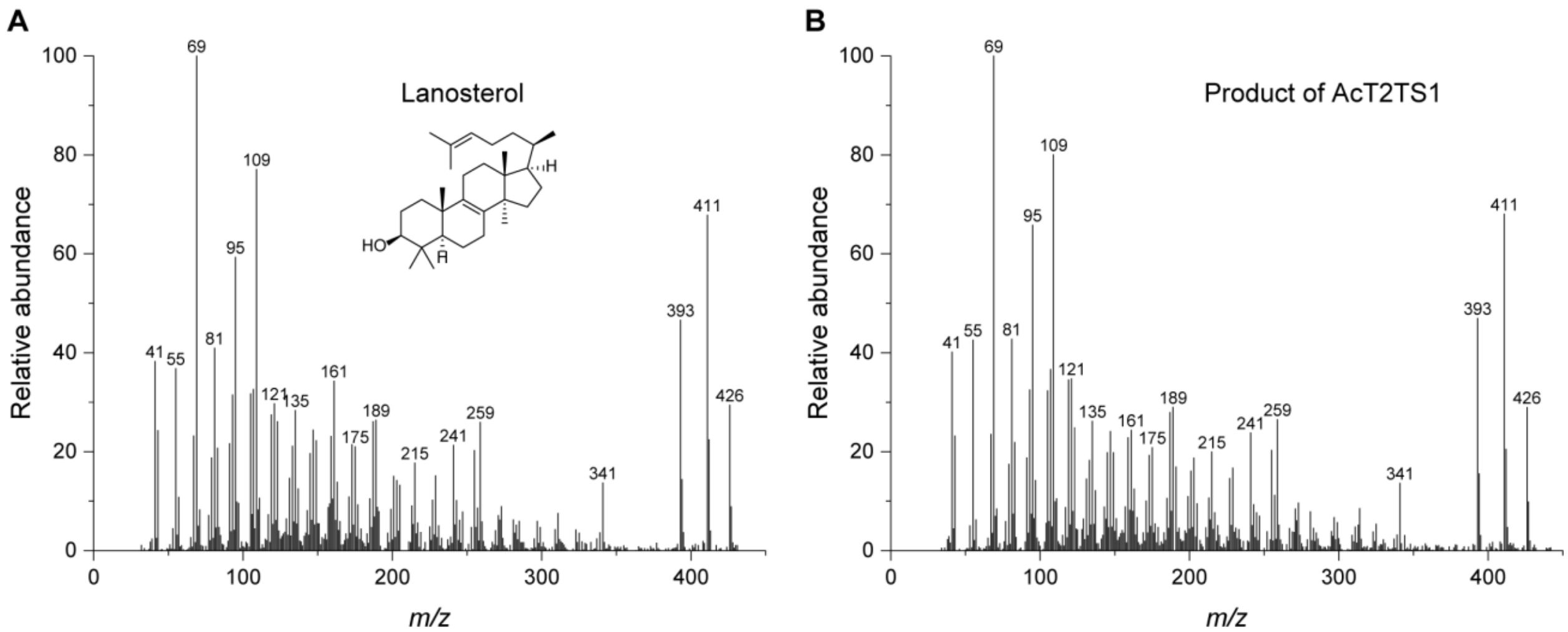

Figure S32. Comparative mass spectrometry analysis of the product of AcT2TS1 versus lanosterol standard.

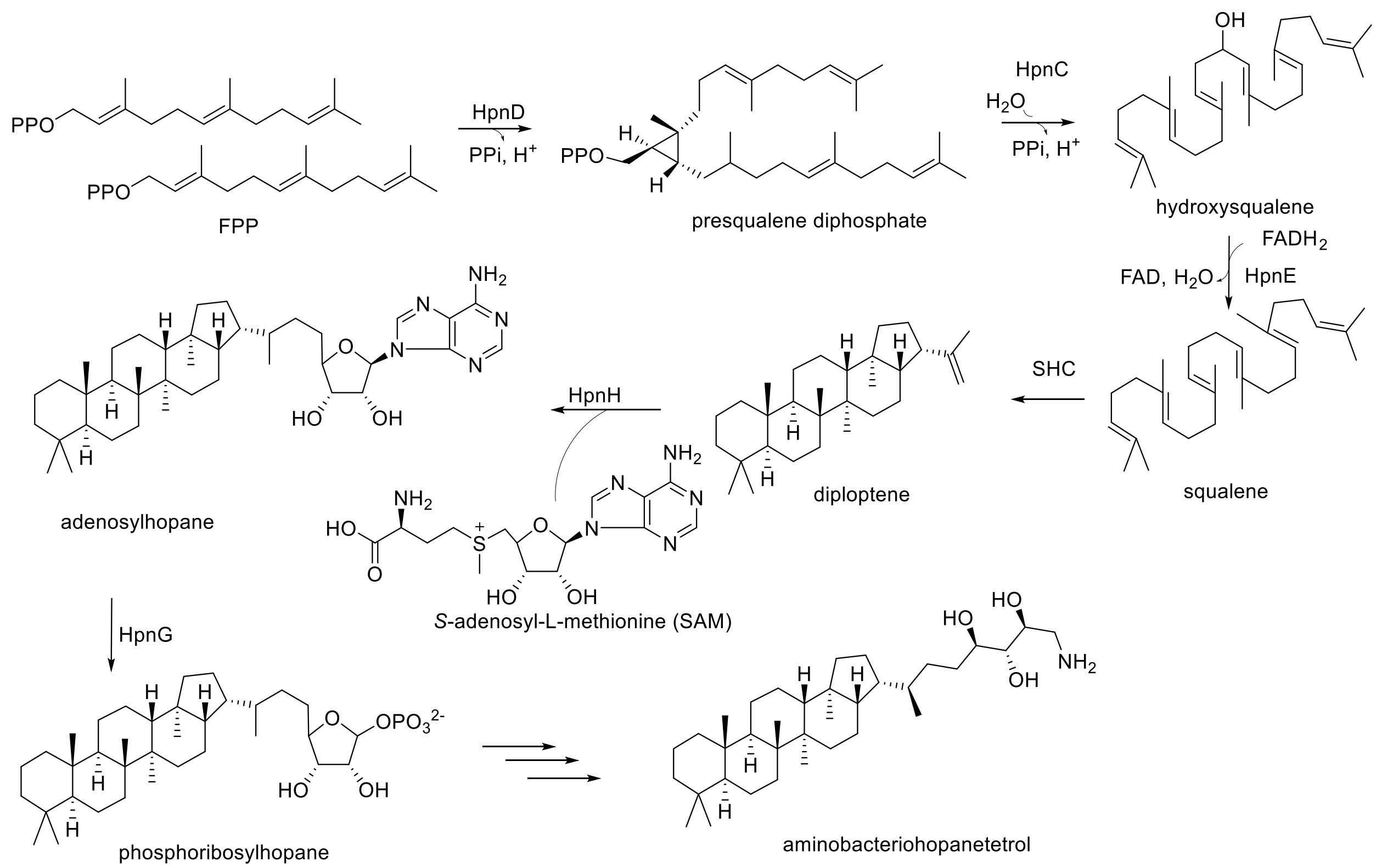

Figure S33. Hopanoid biosynthesis in prokaryotes.

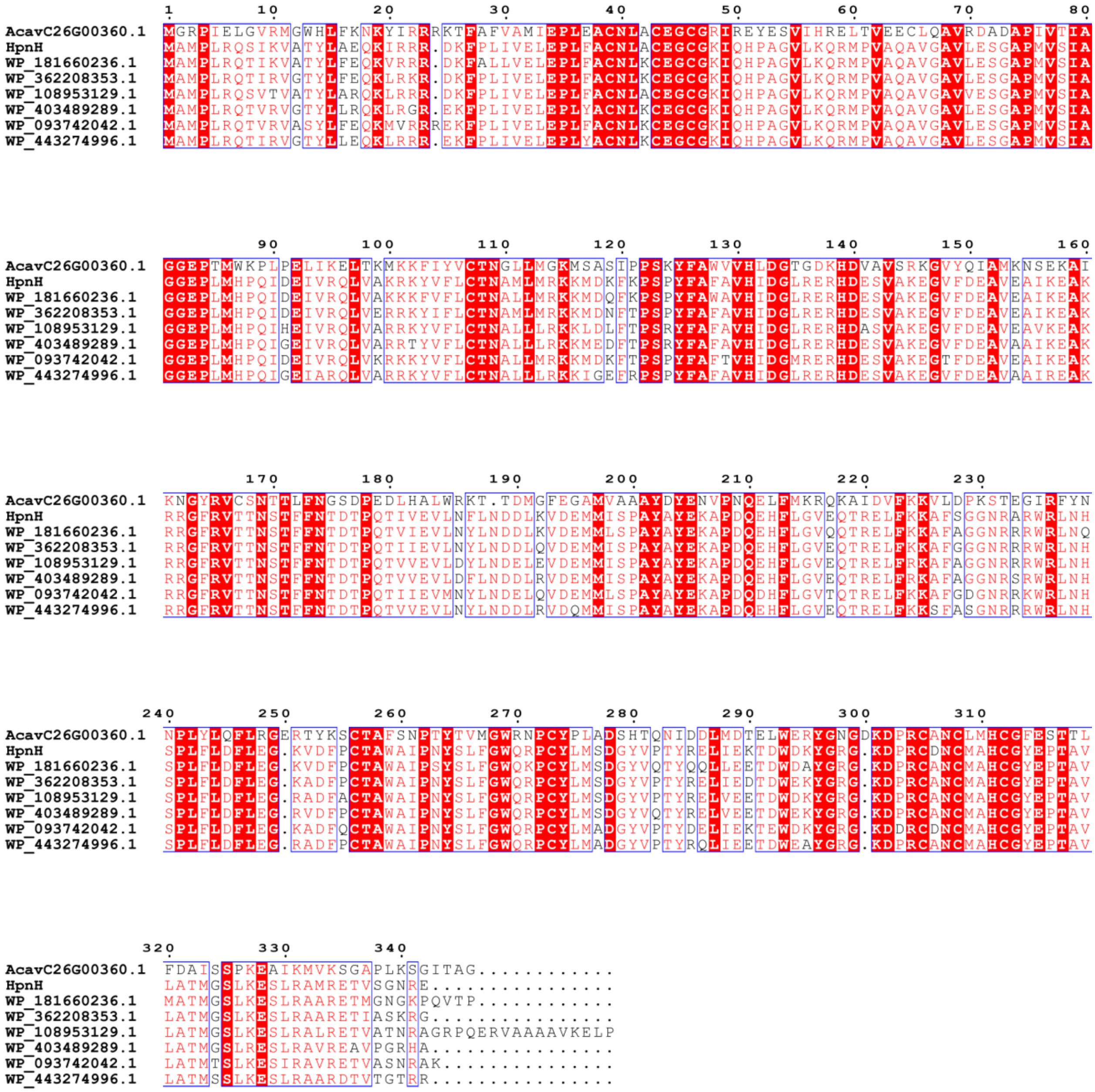

Figure S34. Multiple sequence alignment of AcavC26G0036 (36) with bacteria radical SAM enzymes.

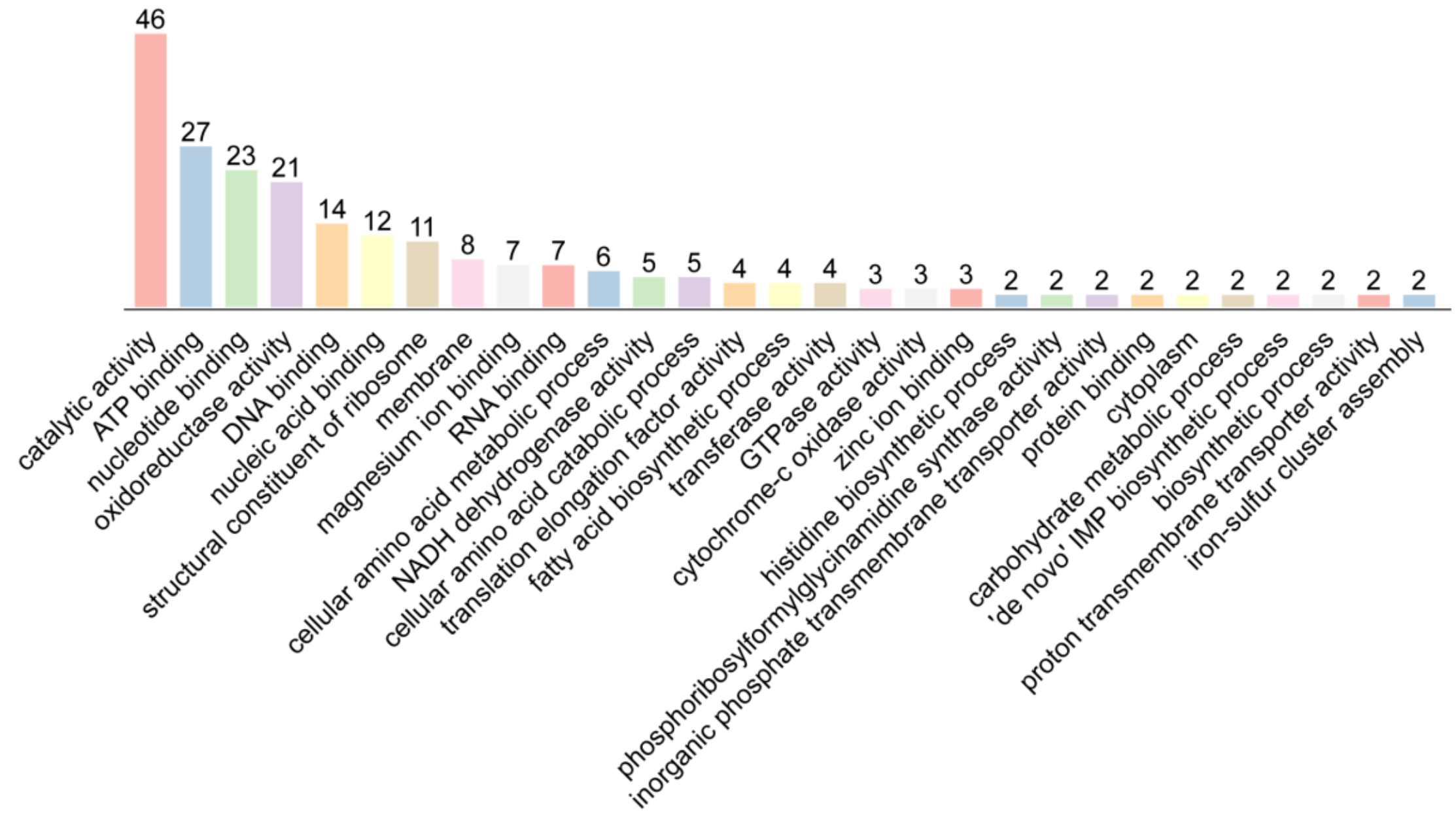

Figure S35. GO enrichment analysis results for genes on contig26.

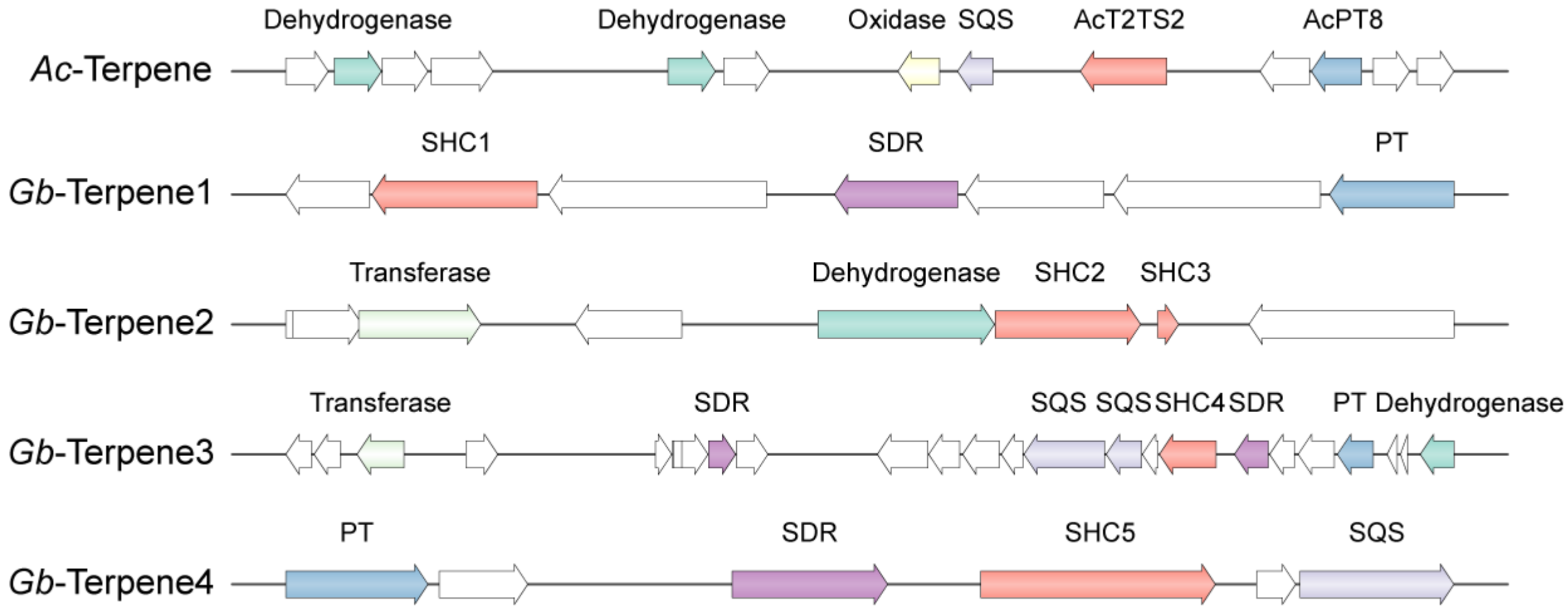

Figure S36. Terpenoid gene clusters in sponges.

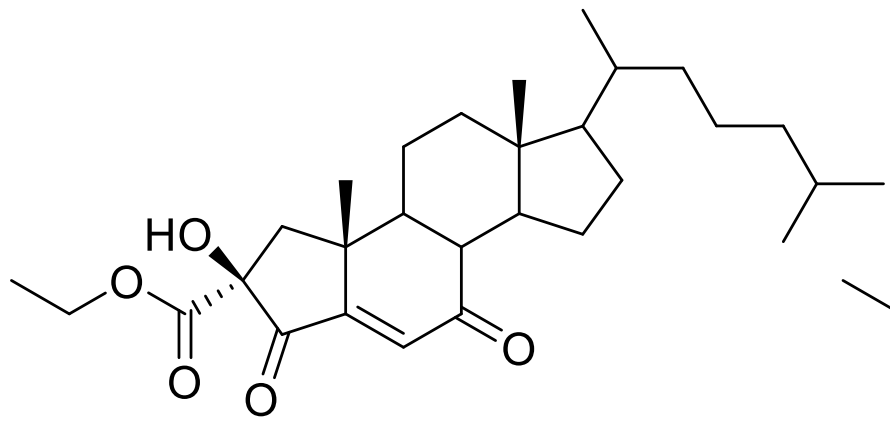

2β-Hydroxy-4,7-diketo-A-norcholest-5-en-2-oic acid

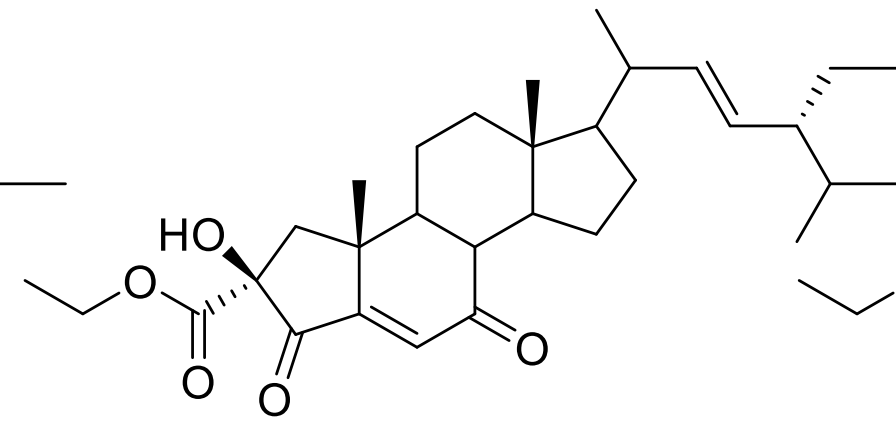

24*S*-Ethyl-2β-hydroxy-4,7-diketo-A-norcholest-5-en-2-oicacid

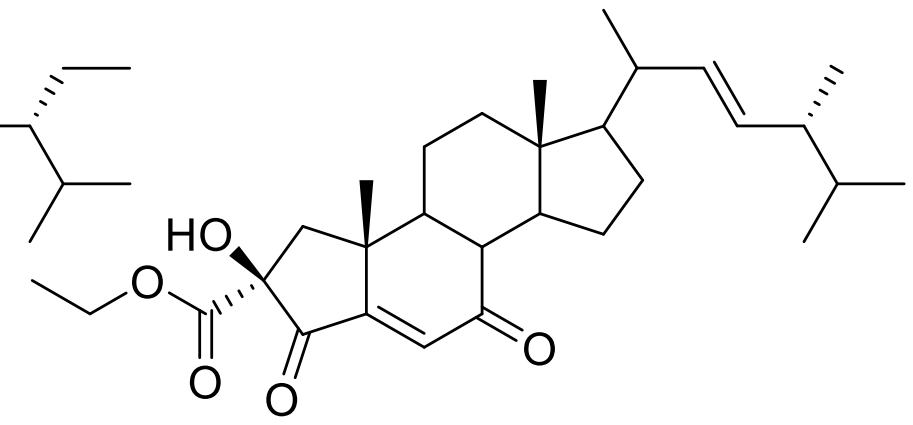

2β-Hydroxy-4,7-diketo-24*R*-methyl-A-norcholest-5,22(*E*)-diene-2-oic acid

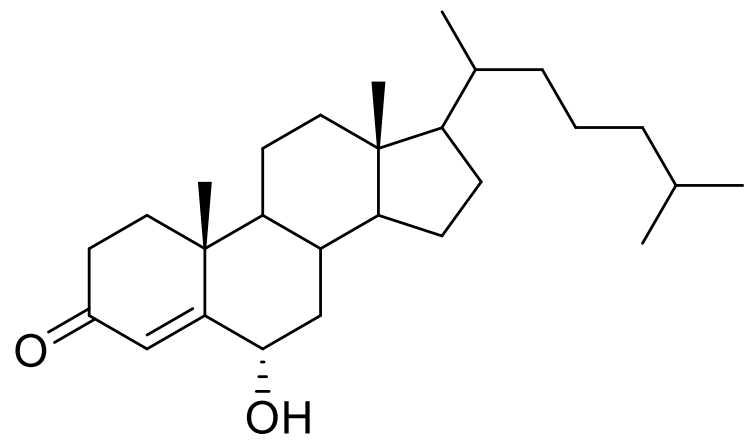

6α-Hydroxycholest-4-en-3-one

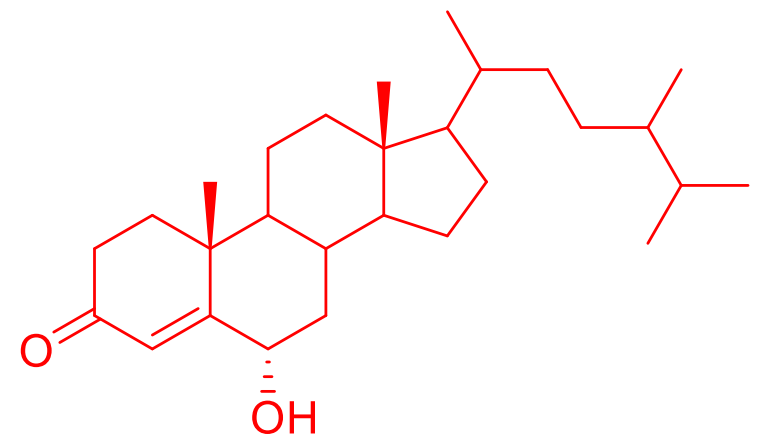

6α-Hydroxyergost-4-en-3-one

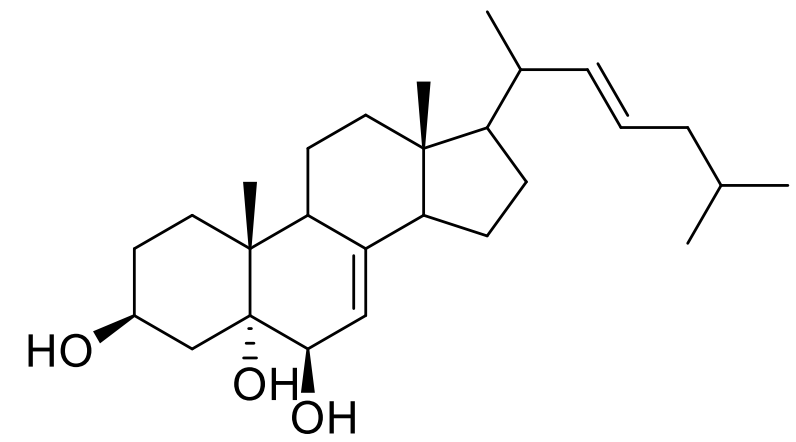

Cholest-7,22E-dien-3β,5α,6β-triol

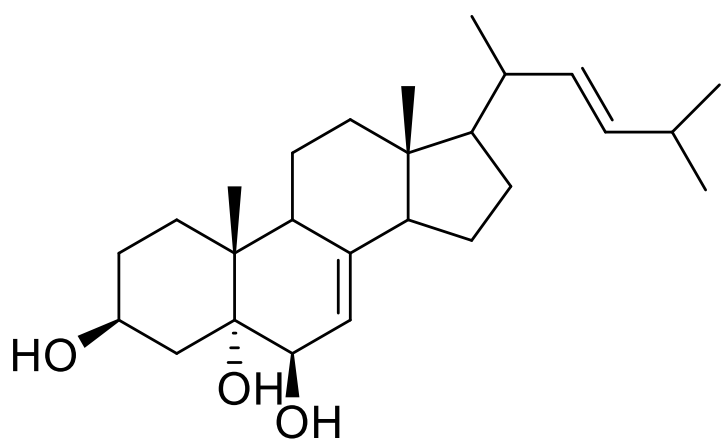

24-Norcholest-7,22*E*-dien-3β,5α,6β-triol

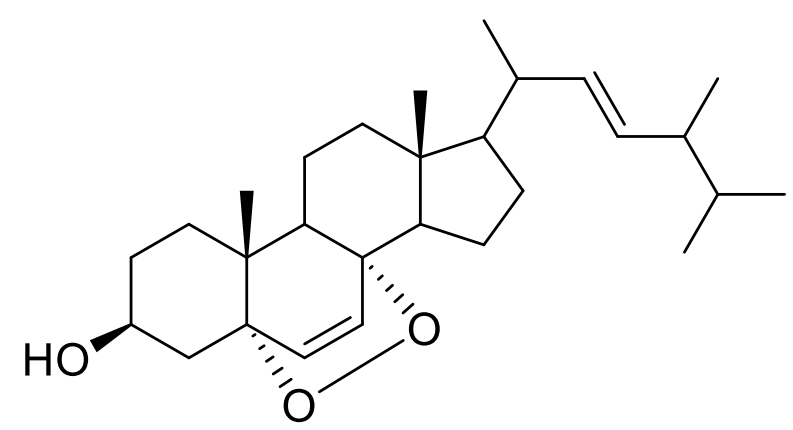

5α,8α-Epidioxy-(22*E*,24*R*)-erost-6,22-dien-3β-ol

Figure S37. Diverse Sterols in the *A. cavernosa* sponge. The red-labeled compound was shown in Figure 8.

Supplementary Tables

Table S1. Sequencing quality statistics of the *A. cavernosa* sponge.

	Q20 (%)	Q30 (%)	GC content (%)
Hi-C	99.55;98.68	98.22;95.96	42.76;42.75
RNA	99.57;98.17	98.24;94.24	50.4;50.39
Survey	98.88	96.38	42.96

Table S2. RNA-seq data from different body regions of *A. cavernosa* sponge.

Species	ID	Clean Reads	Clean Base (G)	Q30 (%)	GC (%)	Genome Mapping Ratio (%)	Gene Mapping Ratio (%)
A. cavernosa	A.Cav-1	24,880,313	7,464,093,900	93.76	49.93	92.68	31.07
	A.Cav-2	22,056,985	6,617,095,500	93.98	50.03	92.9	31.58
	A.Cav-3	22,755,480	6,826,644,000	96.22	50.38	94.96	34.29

Table S3. Hi-C based genome scaffolding length statistics.

	Contig		Scaffold	
	**Length (bp)**	**Numbers**	**Length (bp)**	**Numbers**
N10	6,300,525	4	17,430,531	2
N20	5,002,146	9	16,607,844	3
N30	4,178,830	15	10,337,125	5
N40	3,514,474	22	9,623,482	8
N50	2,695,861	31	9,154,159	11
N60	2,114,736	43	9,005,071	14
N70	1,475,824	57	8,417,352	17
N80	834,255	81	7,495,048	20
N90	287,941	133	6,761,309	24

Table S4. Summary of *A. cavernosa* chromosome level assembly.

Chromosome ID	Chromosome length (bp)	Contig number
Acav01	25,846,332	36
Acav02	17,430,531	46
Acav03	16,607,844	42
Acav04	13,513,098	12
Acav05	10,337,125	8
Acav06	9,696,594	9
Acav07	9,655,317	12
Acav08	9,623,482	5
Acav09	9,390,943	4
Acav10	9,197,274	6
Acav11	9,154,159	10
Acav12	9,115,651	9
Acav13	9,028,227	12
Acav14	9,005,071	7
Acav15	8,699,674	8
Acav16	8,575,783	8
Acav17	8,417,352	5
Acav18	8,199,063	3
Acav19	7,652,084	7
Acav20	7,495,048	5
Acav21	7,464,980	6
Acav22	7,163,147	4
Acav23	6,993,693	1
Acav24	6,761,309	4
Acav25	6,299,565	2
Total anchored length	251,200,346	
Unanchored length	15,666,061	
Non N length	266,866,407	
Total length	266,989,407	

Table S5. Various sponge genome assembly metrics.

Species	Assembly size (Mb)	Total scaffolds	Total contigs	Contig N50 (Mb)	Longest contig (Mb)	Pseudochromosomes	Gene number	BUSCO
Acanthella cavernosa	266.989	101	347	2.696	8.463	25	16495	91
Amphimedon queenslandica	164.263	13133	27634	0.012	0.218	-	19005	90.8
Corticium candelabrum	185.492	115	392	1.297	4.254	22	20131	89.5
Petrosia ficiformis	191.274	36	111	3.95	10.495	18	-	88.9
Halichondria panicea	131.457	39	219	1.456	4.027	17	17225	87.1
Oscarella lobularis	65.248	21	218	0.506	1.576	20	14436	86.3
Tethya minuta	86.067	244	377	0.698	4.329	-	22779	86
Ephydatia muelleri	322.621	1438	3624	0.212	1.717	23	39245	85.1
Dysidea avara	550.831	108	632	1.714	7.286	15	25265	83.8
Chondrosia reniformis	117.39	15	43	4.706	8.805	14	-	82.3
Geodia barretti	144.789	4535	4537	0.048	0.495	-	31884	82.1
Tethya wilhelma	126.188	557	13132	0.017	0.193	-	27965	81.3
Sycon ciliatum	539.575	618	2391	0.62	3.14	13	20262	79.7
Spongilla lacustris	248.733	71	1495	0.254	1.653	-	-	72.7
Aphrocallistes vastus	80.32	186	296	1.255	4.096	-	20038	71.3
Oopsacas minuta	61.461	365	1038	0.29	1.048	-	16413	69.2
Rhabdastrella globostellata	155.602	329	935	0.804	4.599	21	-	68.8
Xestospongia testudinaria	257.936	97640	215863	0.001	0.059	-	52886	67.8
Oscarella pearsei	57.775	67767	91138	0.003	0.096	-	29220	67.3
Stylissa carteri	418.921	97497	262423	0.002	0.081	-	46515	54.9

Table S6. Comparison of genome Benchmarking Universal Single-Copy Orthologs (BUSCO) of the *A. cavernosa* with other sponge genomes.

Species	Complete BUSCOs(%)	Single-copy BUSCOs(%)	Duplicated BUSCOs(%)	Fragmented BUSCOs(%)	Missing BUSCOs(%)
Acanthella cavernosa	91	87.8	3.1	1.7	7.4
Amphimedon queenslandica	90.8	84.6	6.2	2.3	6.9
Corticium candelabrum	89.5	88.5	1	2.5	8
Petrosia ficiformis	88.9	87	1.9	1.7	9.4
Halichondria panicea	87.1	85.4	1.7	1.6	11.3
Oscarella lobularis	86.3	85	1.3	2	11.7
Tethya minuta	86	82	4	2.6	11.4
Ephydatia muelleri	85.1	83.1	2	3.1	11.8
Dysidea avara	83.8	81.2	2.6	2.9	13.3
Chondrosia reniformis	82.3	81.3	1	2.5	15.2
Geodia barretti	82.1	79.1	3	4.5	13.4
Tethya wilhelma	81.3	79.4	1.9	5.8	12.9
Sycon ciliatum	79.7	75.9	3.8	2.5	17.8
Spongilla lacustris	72.7	71.7	1	3.7	23.6
Aphrocallistes vastus	71.3	70	1.3	1.8	26.9
Oopsacas minuta	69.2	68.6	0.6	2.2	28.6
Rhabdastrella globostellata	68.8	67.2	1.6	3.5	27.7
Xestospongia testudinaria	67.8	65.1	2.7	13.7	18.5
Oscarella pearsei	67.3	67.2	0.1	9.7	23
Stylissa carteri	54.9	48.5	6.4	26.5	18.6

Table S7. Assessing *A. cavernosa* genome and annotation completeness with BUSCO analysis.

BUSCO notation	Genome		Predicted CDS	
	Number	Percentage (%)	Number	Percentage (%)
Complete BUSCOs	868	91	839	87.9
Single-copy BUSCOs	838	87.8	823	86.3
Duplicated BUSCOSs	30	3.1	16	1.7
Fragemented BUSCOs	17	1.7	32	3.4
Missing BUSCOs	69	7.4	83	8.7
Total	954	100	954	100

Table S8. Statistics of repeated elements in the *A. cavernosa* genome.

	**Repbase TEs**		**TE protiens**		**De novo**		**Combined TEs**	
Type	Length (Bp)	% in genome	Length (Bp)	% in genome	Length (Bp)	% in genome	Length (Bp)	% in genome
DNA	4,568,460	1.711	116,567	0.044	2,366,911	0.887	6,866,946	2.572
LINE	1,568,773	0.588	173,896	0.065	11,400,078	4.270	12,689,965	4.753
SINE	81,396	0.030	0	0.000	0	0.000	81,396	0.030
LTR	3,064,849	1.148	2,100,580	0.787	101,104,104	37.868	102,077,291	38.233
Other	386	0.000	585	0.000	0	0.000	971	0.000
Unknown	83,995	0.031	0	0.000	40,124,838	15.029	40,191,702	15.054
Total	8,557,187	3.205	2,400,273	0.899	144,670,244	54.186	146,186,236	54.754

Table S9. Prediction of protein-coding genes in the *A. cavernosa* genome.

Method	Software	Species	Total genes predicted	Average gene length (bp)	Average CDS length (bp)	Average exon number per gene	Average exon length (bp)	Average intron length (bp)
De novo	Augustus		17,158	4,746.51	1,241.98	4.66	266.32	956.63
	SNAP		22,818	18259.44	1083.49	5.24	206.9	4053.92
Homolog		*Amphimedon queenslandica*	2,295	7540.55	1000.94	5.77	173.58	1372.01
		Geodia barretti	7,053	5037.09	833.68	3.16	263.73	1945.07
		Halichondria panicea	5,175	9933.5	1253.91	5.15	243.67	2093.54
		Ephydatia muelleri	8,701	5553.59	1130.23	3.59	314.55	1705.79
		Tethya minuta	4,584	5525.53	823.01	4.94	166.59	1193.4
		Tethya wilhelma	11,648	5196.54	918.38	3.1	296.14	2036.12
		Xestospongia testudinaria	19,274	1786.83	602.37	1.69	355.95	1710.96
RNA-seq			27,594	10750.16	1892.56	9.47	199.81	1045.56
Integration			16,495	8745.47	1400.04	6.89	202.92	1245.08

Table S10. Functional annotation of the predicted genes for *A. cavernosa*.

	Database	Number	Percentage (%)
Total		16,495	100%
Annotated	NR	14,934	90.53%
	SwissProt	8,108	49.15%
	KEGG	9,973	60.46%
	TrEMBL	14,942	90.58%
	Interpro	12,508	75.83%
	GO	8,723	52.88%
Overall		15,322	92.89%

Table S11. Comparison of gene BUSCO of the *A. cavernosa* with other sponge genomes.

Species	Complete BUSCOs	single-copy BUSCOs	duplicated BUSCOs	Fragmented BUSCOs	Missing BUSCOs
Corticium candelabrum	92.80%	91.80%	0.90%	2.80%	4.40%
Dysidea avara	91.90%	89.50%	2.40%	1.30%	6.80%
Halichondria panicea	91.20%	89.40%	1.80%	1.30%	7.50%
Amphimedon queenslandica	90.10%	84.20%	6.00%	2.90%	6.90%
Oscarella lobularis	89.10%	87.80%	1.30%	1.20%	9.70%
Acanthella cavernosa	87.9%	86.3%	1.7%	3.4%	8.7%
Sycon ciliatum	86.90%	82.90%	4.00%	1.90%	11.20%
Tethya wilhelma	83.50%	81.00%	2.50%	7.00%	9.40%
Aphrocallistes vastus	80.60%	78.80%	1.80%	2.10%	17.30%
Geodia barretti	74.20%	71.70%	2.50%	9.90%	15.90%
Ephydatia muelleri	70.10%	57.80%	12.40%	7.20%	22.60%
Xestospongia testudinaria	63.20%	59.60%	3.60%	12.90%	23.90%
Tethya minuta	57.90%	52.10%	5.80%	19.50%	22.60%
Stylissa carteri	42.80%	38.90%	3.90%	28.70%	28.50%

Table S12. Genomic resource used for analysis in this study.

Species	links
Amphimedon queenslandica	https://www.ncbi.nlm.nih.gov/assembly/GCF_000090795.2
Geodia barretti	https://www.ncbi.nlm.nih.gov/datasets/genome/GCA_030341885.1/
Tethya minuta	https://github.com/PalMuc/2Tethya_genomes
Ephydatia muelleri	https://www.ncbi.nlm.nih.gov/assembly/GCA_013339895.1
Halichondria panicea	https://www.ncbi.nlm.nih.gov/datasets/genome/GCF_963675165.1/
Tethya wilhelma	https://github.com/PalMuc/2Tethya_genomes
Xestospongia testudinaria	http://xt.reefgenomics.org/download/
Corticium candelabrum	https://www.ncbi.nlm.nih.gov/datasets/genome/GCF_963422355.1/
Oscarella lobularis	https://ftp.ncbi.nlm.nih.gov/genomes/all/GCF/947/507/565/GCF_947507565.1_ooOscLobu1.1/
Dysidea avara	https://www.ncbi.nlm.nih.gov/datasets/genome/GCF_963678975.1/
Sycon ciliatum	https://www.ncbi.nlm.nih.gov/datasets/genome/GCF_964019385.1/
Stylissa carteri	http://sc.reefgenomics.org/download/
Aphrocallistes vastus	https://github.com/PalMuc/Aphrocallistes_vastus_genome
Chondrosia reniformis	https://www.ncbi.nlm.nih.gov/datasets/genome/GCA_947172415.1/
Spongilla lacustris	https://www.ncbi.nlm.nih.gov/datasets/genome/GCA_949361645.1/
Oopsacas minuta	https://www.ncbi.nlm.nih.gov/datasets/genome/GCA_024704765.1/
Oscarella pearsei	https://figshare.com/articles/dataset/Oscarella_pearsei_assemblies/7107638
Rhabdastrella globostellata	https://www.ncbi.nlm.nih.gov/datasets/genome/GCA_964261875.1/
Petrosia ficiformis	https://www.ncbi.nlm.nih.gov/datasets/genome/GCA_947044365.1/

Table S13. Subcellular localizations of MVA and MEP genes in *A. cavernosa*.

Species	Pathway	Gene ID	Name	Location
	MEP	AcavC48G00110.1	AcDXS	noTP
	MEP	AcavC26G03080.1	AcDXR1	noTP
	MEP	AcavC27G00380.1	AcDXR2	noTP
	MEP	AcavC28G00920.1	AcDXR3	noTP
	MEP	AcavC34G00290.1	AcDXR4	noTP
	MEP	AcavC28G00150.1	AcMCT	noTP
	MEP	AcavC26G02290.1	AcMDS1	noTP
	MEP	AcavC27G01400.1	AcMDS2	noTP
	MEP	AcavC28G00140.1	AcMDS3	noTP
	MEP	AcavC43G00390.1	AcMDS4	noTP
	MEP	AcavC26G03070.1	AcHDS1	noTP
	MEP	AcavC27G01330.1	AcHDS2	noTP
	MEP	AcavC28G01610.1	AcHDS3	noTP
	MEP	AcavC26G00220.1	AcHDR1	noTP
A. cavernosa	MEP	AcavC28G01710.1	AcHDR2	noTP
	MEP	AcavC51G00100.1	AcHDR3	noTP
	MVA	Acav05G00740.1	AcAACT1	MitoTP
	MVA	Acav11G04920.1	AcAACT2	MitoTP
	MVA	Acav16G04440.1	AcAACT3	MitoTP
	MVA	Acav18G05350.1	AcAACT4	noTP
	MVA	Acav22G00810.1	AcAACT5	noTP
	MVA	AcavC27G01050.1	AcAACT6	noTP
	MVA	AcavC28G01070.1	AcAACT7	noTP
	MVA	AcavC28G01080.1	AcAACT8	noTP
	MVA	AcavC28G01380.1	AcAACT9	noTP
	MVA	AcavC37G00020.1	AcAACT10	noTP
	MVA	Acav18G00690.1	AcHMGS	noTP
	MVA	Acav21G01970.1	AcHMGR1	noTP
	MVA	Acav21G01990.1	AcHMGR2	noTP

	MVA	Acav25G00930.1	AcHMGR3	noTP
	MVA	AcavC30G00320.1	AcHMGR4	noTP
	MVA	Acav17G04430.1	AcMVK	noTP
	MVA	Acav25G00450.1	AcPMK1	noTP
	MVA	Acav25G01020.1	AcPMK2	noTP
	MVA	Acav24G03000.1	AcMVD	noTP
	MVA	Acav12G03550.1	AcIDI1	noTP
	MVA	Acav24G04960.1	AcIDI2	noTP

MEP: methylerythritol phosphate pathway; MVA: mevalonate pathway; noTP: no targeting signal; MitoTP: mitochondrial targeting signal.

Table S14. Curated TPS database of marine animals.

IDs	Type	IDs	Type
Paramuricea_biscaya_TPS-1	T1TS	EcTPS6	T1TS
D_gigantea_TPS-1	T1TS	CiTC-1	T1TS
D_gigantea_TPS-2	T1TS	DgTC-2	T1TS
E_rubra-TPS-1	T1TS	TmTC-1	T1TS
E_rubra-TPS-2_7S5L_A	T1TS	uBuTS-1_undescribed_Bubarida	T1TS
B.asbestinum_TPS-1	T1TS	uBuTS-5-Sponge	T1TS
B.asbestinum_TPS-2	T1TS	uBuTS-6-Sponge	T1TS
Xenia_TPS-1_trans	T1TS	uBuTS-7-Sponge	T1TS
Heliopora_coerulea_TPS-1	T1TS	uBuTS-10-Sponge	T1TS
Heliopora_coerulea_TPS-2	T1TS	uBuTS-11-Sponge	T1TS
Heliopora_coerulea_TPS-3	T1TS	uBuTS-13-Sponge	T1TS
Renilla_reniformis_TPS-1	T1TS	PhTS-1-Sponge	T1TS
Tubipora_musica_TPS-2	T1TS	AgTS-1-Sponge	T1TS
XM_065325624.1_prot_XP_065181696.1_1	T1TS	StTS-1-Sponge	T1TS
XM_065335917.1_prot_XP_065191989.1_1	T1TS	AgTS-2-Sponge	T1TS
OP977975.1_prot_WDE20676.1_1	T1TS	A0A1X7VQB7	T2TS
OP977976.1_prot_WDE20677.1_1	T1TS	A0AA35RE98	T2TS
OP977977.1_prot_WDE20678.1_1	T1TS	A0AA35RFS7	T2TS
OP977978.1_prot_WDE20679.1_1	T1TS	A0AA35R6Z0	T2TS
OP977979.1_prot_WDE20680.1_1	T1TS	A0AA35RFS0	T2TS
OP977980.1_prot_WDE20681.1_1	T1TS	A0AA35RGV4	T2TS
OP977981.1_prot_WDE20682.1_1	T1TS	A0AA35S0J2	T2TS
OP977982.1_prot_WDE20683.1_1	T1TS	A0AA35SHD1	T2TS
OP977983.1_prot_WDE20684.1_1	T1TS	A0AA35T990	T2TS
A0A6S7GFJ4	T1TS	AmphiLSS-Sponge	T2TS
A0A6S7GM34	T1TS	SaccoLSS	T2TS
A0A6S7HXL7	T1TS	HelobLSS	T2TS
A0A6S7IQD1	T1TS	SpLSS	T2TS
A0A6S7IRN7	T1TS	EpLSS	T2TS
A0A6S7K0M9	T1TS	AfLSS	T2TS
A0A7D9ETX6	T1TS	ArLSS	T2TS
A0A7D9JFW3	T1TS	LspLSS	T2TS
A0A7D9M5R7	T1TS	PmLSS	T2TS

A0A8T9VTI8	T1TS	ApLSS	T2TS
A0A8T9VVV6	T1TS	EsLSS	T2TS
A0A8T9VYE5	T1TS	HspLSS	T2TS
A0A8T9VYK6	T1TS	AjLDS-b	T2TS
A0A8T9VYQ6	T1TS	AjLDS-a	T2TS
A0A8T9VYX1	T1TS	PpLDS	T2TS
A0A8T9W2X1	T1TS	ScLDS	T2TS
PcTS-4	T1TS	AjPS-b	T2TS
PcTS-7	T1TS	AjPS-a	T2TS
PcTS-11	T1TS	PpPS	T2TS
EcTPS1	T1TS	ScPS	T2TS

Table S15. ^{1}H and ^{13}C NMR data for (+)-*β*-cubebene in $CDCl_3$.

No.	**(+)-*β*-cubebene**	
	δ_H, mult (*J* in Hz)	δ_C, type
1	0.83, m	28.7, CH
2	1.39, m	36.0, CH
3	-	155.5, C
4	2.12, m	29.2, CH_2
	2.04, m	
5	1.98, m	30.9, CH_2
	1.55, m	
6	-	38.2, CH
7	1.79, m	31.2, CH
8	1.61, m	31.7, CH_2
	0.57, m	
9	1.40, m	26.4, CH_2
	0.86, m	
10	1.11, m	44.2, CH
11	1.59, m	33.6, CH
12	0.89, d (6.8)	19.6, CH_3
13	0.92, d (6.7)	20.2, CH_3
14	0.95, d (6.5)	19.0, CH_3
15	4.76, s	101.0, CH_2
	4.57, s	

Table S16. Percent identity matrix of AcT1TS3, AcT1TS4, AcT1TS5 and *β*-cubebene synthase from *M. grandiflora* (MgBCS).

**Seq**	MgBCS	AcT1TS5	AcT1TS3	AcT1TS4
MgBCS	100	21.9	21.53	21.53
AcT1TS5		100	99.09	99.39
AcT1TS3			100	99.7
AcT1TS4				100

Table S17. RMSD values from structural alignment of TPS proteins between the *A. cavernosa* sponge and those from plants or bacteria.

**Structure1**	**Structure2**	**RMSD**	**Method**
AcT1TS3	MgBCS	4.726	cealign
AcT1TS4	MgBCS	4.728	cealign
AcT1TS5	MgBCS	4.699	cealign
AcT1TS10	SlABS	4.422	super
AcT1TS10	PaABS	4.878	cealign
AcUbiA1	AciTS	2.121	align
AcUbiA1	SpuTS	2.480	align
AcUbiA1	AduTS	2.680	align
AcUbiA1	HarTS	2.331	align
AcUbiA1	AriTS	2.904	align

Table S18. Percent identity matrix of AcT1TS10 with *α*-bisabolene synthase from *Picea abies* (PaABS) and *Solanum lycopersicum* (SlABS).

**Seq**	AcT1TS10	SlABS	PaABS
AcT1TS10	100	18.43	22.35
SlABS		100	28.77
PaABS			100

Table S19. Primers used in this study.

Name	Sequence (5' to 3')	Expression host
AcPT1-F	AGCAAATGGGTCGCGGATCCATGGCCGCCAAAGATGAAGC	*E. coli*
AcPT1-R	TCGAGTGCGGCCGCAAGCTTTCACTTGCTTGCAGGTGACG	*E. coli*
AcPT4-F	AGCAAATGGGTCGCGGATCCATGAGTGAACCGCCCCCAGC	*E. coli*
AcPT4-R	TCGAGTGCGGCCGCAAGCTTTCAGTTCTTCCTCTTGTATA	*E. coli*
AcPT16-F	AGCAAATGGGTCGCGGATCCATGAATGAGGCCGCCTTGAG	*E. coli*
AcPT16-R	TCGAGTGCGGCCGCAAGCTTTTACAAACTCCTTTTTACAA	*E. coli*
AcT1TS1-F	AGCAAATGGGTCGCGGATCCATGTCACTCAGTGTGTTTGC	*E. coli*
AcT1TS1-R	TCGAGTGCGGCCGCAAGCTTTCACACAGAGTAGCGTTTTG	*E. coli*
AcT1TS2-F	AGCAAATGGGTCGCGGATCCATGCATTATGCAGACTCTAC	*E. coli*
AcT1TS2-R	TCGAGTGCGGCCGCAAGCTTGTGTGGTGTAATTGGAAGCC	*E. coli*
AcT1TS3-F	AGCAAATGGGTCGCGGATCCATGTCACTCAGTGTATTTAA	*E. coli*
AcT1TS3-R	TCGAGTGCGGCCGCAAGCTTTCATAAAGTATAACGCCTTG	*E. coli*
AcT1TS4-F	AGCAAATGGGTCGCGGATCCATGTCACTCAGTGTATTTAA	*E. coli*
AcT1TS4-R	TCGAGTGCGGCCGCAAGCTTTCATAAAGTATAACGCCTTG	*E. coli*
AcT1TS5-F	AGCAAATGGGTCGCGGATCCATGTCACTCAGTGTATTTAA	*E. coli*
AcT1TS5-R	TCGAGTGCGGCCGCAAGCTTTCATAAAGTATAACGCCTTG	*E. coli*
AcT1TS7-F	AGCAAATGGGTCGCGGATCCATGTCACTCAGTATGTTTGC	*E. coli*
AcT1TS7-R	TCGAGTGCGGCCGCAAGCTTCTAATTATAACGTTTTGAAT	*E. coli*
AcT1TS8-F	AGCAAATGGGTCGCGGATCCATGTCACTCAGTGTGTTTGC	*E. coli*
AcT1TS8-R	TCGAGTGCGGCCGCAAGCTTTCACAGGGTATAACGTTTTG	*E. coli*
AcT1TS9-F	AGCAAATGGGTCGCGGATCCATGTCACTCAGCAAGTTTAC	*E. coli*
AcT1TS9-R	TCGAGTGCGGCCGCAAGCTTTTAGCATGAAATGATTGCAG	*E. coli*
AcT1TS10-F	AGCAAATGGGTCGCGGATCCATGTCACTCAGTGTGTTCAC	*E. coli*
AcT1TS10-R	TCGAGTGCGGCCGCAAGCTTTTAACTTGCATGGAATCCAG	*E. coli*
AcT1TS6-F	GGAGAAAAAACCCCGGATCCATGAAGATTTCTGTCCCTGCAGT	*S. cerevisiae*
AcT1TS6-R	CGGTACCAAGCTTACTCGAGATAAGATTCTGCATGGTACT	*S. cerevisiae*
AcUbiA1-F	AGCAAATGGGTCGCGGATCCATGAGGATGATGAAGATCACTG	*E. coli*
AcUbiA1-R	TCGAGTGCGGCCGCAAGCTTTTACAAATATGCAATAAGATTCA	*E. coli*
AcUbiA2-F	AGCAAATGGGTCGCGGATCCATGGATAGCAGCAAGCGACTTC	*E. coli*
AcUbiA2-R	TCGAGTGCGGCCGCAAGCTTTCAGCTGACTGCAACCACATCTG	*E. coli*
AcUbiA3-F	AGCAAATGGGTCGCGGATCCATGGAGCTTCCCACTAGAGAAG	*E. coli*
AcUbiA3-R	TCGAGTGCGGCCGCAAGCTTTCATGTAACAGAAAGAAAGGGAA	*E. coli*
AcUbiA4-F	AGCAAATGGGTCGCGGATCCATGAGTGAGGCACAGAGGAGGA	*E. coli*
AcUbiA4-R	TCGAGTGCGGCCGCAAGCTTCTATGTGACAAATGAGGCCAAGT	*E. coli*
AcUbiA5-F	AGCAAATGGGTCGCGGATCCATGACCTCAGCTGACCTCAGTG	*E. coli*
AcUbiA5-R	TCGAGTGCGGCCGCAAGCTTCTATATCACCGCTGAATCTATCA	*E. coli*

AcUbiA6-F	AGCAAATGGGTCGCGGATCCATGTATGCTGCAAGCAAGCTAA	*E. coli*
AcUbiA6-R	TCGAGTGCGGCCGCAAGCTTTTAGAACCTAAAATGAAATGCAG	*E. coli*
AcUbiA7/8/10-F	AGCAAATGGGTCGCGGATCCATGATGCTCGCCGCCCGCGATT	*E. coli*
AcUbiA7/8/10-R	TCGAGTGCGGCCGCAAGCTTTCATTCGCTGAGGATGGCGCGCA	*E. coli*
AcUbiA9-F	AGCAAATGGGTCGCGGATCCATGCGTTCAGCCGGTTGTGCGA	*E. coli*
AcUbiA9-R	TCGAGTGCGGCCGCAAGCTTTTAGTAGGATCGGTTCAACAGAA	*E. coli*
tHMGR-F	ATTTTTGAAAATTCGAATTCATGAATCCGGAACCTATTGTTACC	*S. cerevisiae*
tHMGR-R	GAATTGTTAATTAAGAGCTCATGTTGTTGTTGTTGTCGTTGT	*S. cerevisiae*
AcT2TS1-F	GGAGAAAAAACCCCGGATCCATGTCCGAAGAAGGTAACGG	*S. cerevisiae*
AcT2TS1-R	CGGTACCAAGCTTACTCGAGTTAAGAAGTGATAGAAGTAGATG	*S. cerevisiae*
AcT2TS2-F	GGAGAAAAAACCCCGGATCCATGAACGAGGAAAAGCAAAA	*S. cerevisiae*
AcT2TS2-R	CGGTACCAAGCTTACTCGAGTTAACCAGTAGCAGTGTATTGAG	*S. cerevisiae*
